# Supplementary material for: Incident allergic diseases in post-COVID-19 condition: multinational cohort studies from South Korea, Japan and the UK
Source: Nat Commun. 2024 Apr 2;15:2830. doi: 10.1038/s41467-024-47176-w (PMC10987608; doi:10.1038/s41467-024-47176-w)
Supplement: Supplementary file 1 — Supplementary Information [file 41467_2024_47176_MOESM1_ESM.pdf]

Supplementary Material

Original Article

Incident allergic diseases in post-COVID-19 condition: multinational cohort studies from South Korea, Japan and the UK

Running head: Allergic diseases and long COVID

Jiyeon Oh,[1¶] Myeongcheol Lee,[2,3¶] Minji Kim,[2,3¶] Hyeon Jin Kim,[2,3¶] Seung Won Lee,[4] Sang Youl Rhee,[2,5] Ai Koyanagi,[6] Lee Smith,[7] Min Seo Kim,[8] Hayeon Lee,[9*] Jinseok Lee,[9,10*] Dong Keon Yon,[2,3,11*§]

¶ These authors contributed equally: JO, ML, MK, HJK.

* These authors jointly supervised the work: HL, JL, DKY.

§ DKY is a senior author.

Corresponding authors

Dong Keon Yon, MD, PhD, FACAAI, FAAAAI (lead contact)

Department of Pediatrics, Kyung Hee University College of Medicine 23 Kyungheedae-ro, Dongdaemun-gu, Seoul 02447, South Korea

Tel: +82-2-6935-2476

Fax: +82-504-478-0201

Email: yonkkang@gmail.com

Contents of Supplementary appendix

**Supplementary Material**	
**Figure S1**	Density plot and box plot for before matching and after matching in patients with COVID-19 and non-COVID-19 in main cohort (South Korea)
**Figure S2**	Density plot and box plot for before matching and after matching in patients with COVID-19 and non-COVID-19 in replication cohort A (Japan)
**Figure S3**	Density plot and box plot for before matching and after matching in patients with COVID-19 and non-COVID-19 in replication cohort B (UK)
**Figure S4**	Study flow of cohorts
**Table S1**	Definitions of diseases.
**Table S2**	Representative population.
**Table S3**	Baseline characteristics for the full unmatched cohorts of South Korea, Japan and the UK.
**Table S4**	Baseline characteristics for 1:5 propensity score-matched cohort in replication cohort A (Japan, n=2,541,021).
**Table S5**	Baseline characteristics for 1:5 propensity score-matched cohort in replication cohort B (UK, n=325,843).
**Table S6**	Statistical analyses and justification.
**Table S7**	The propensity-score-matched subgroup analysis of HR (95% CI) of asthma following COVID-19 diagnosis stratified by COVID-19 severity, SARS-CoV-2 strain type, and number of vaccinations in main cohort (South Korea).
**Table S8**	The propensity-score-matched subgroup analysis of HR (95% CI) of allergic rhinitis following COVID-19 diagnosis stratified by COVID-19 severity, SARS-CoV-2 strain type, and number of vaccinations in main cohort (South Korea).
**Table S9**	The propensity-score-matched subgroup analysis of HR (95% CI) of atopic dermatitis following COVID-19 diagnosis stratified by COVID-19 severity, SARS-CoV-2 strain type, and number of vaccinations in main cohort (South Korea).
**Table S10**	The propensity-score-matched subgroup analysis of HR (95% CI) of atopic dermatitis following COVID-19 diagnosis stratified by COVID-19 severity, SARS-CoV-2 strain type, and number of vaccinations in main cohort (South Korea).
**Table S11**	Stratification analysis for the long-term sequelae risk of incident allergic diseases following COVID-19 diagnosis in the propensity score-matched main cohort (South Korea).

**Table S12**	Stratification analysis for the long-term sequelae risk of incident asthma following COVID-19 diagnosis in the propensity score-matched main cohort (South Korea)
**Table S13**	Stratification analysis for the long-term sequelae risk of incident allergic rhinitis following COVID-19 diagnosis in the propensity score-matched main cohort (South Korea)
**Table S14**	Stratification analysis for the long-term sequelae risk of incident atopic dermatitis following COVID-19 diagnosis in the propensity score-matched main cohort (South Korea)
**Table S15**	Stratification analysis for the long-term sequelae risk of incident food allergy following COVID-19 diagnosis in the propensity score-matched main cohort (South Korea)
**Table S16**	Stratification analysis for the long-term sequelae risk of incident allergic diseases following COVID-19 diagnosis in the propensity score-matched replication cohort A (Japan)
**Table S17**	Stratification analysis for the long-term sequelae risk of incident asthma following COVID-19 diagnosis in the propensity score-matched replication cohort A (Japan)
**Table S18**	Stratification analysis for the long-term sequelae risk of incident allergic rhinitis following COVID-19 diagnosis in the propensity score-matched replication cohort A (Japan)
**Table S19**	Stratification analysis for the long-term sequelae risk of incident atopic dermatitis following COVID-19 diagnosis in the propensity score-matched replication cohort A (Japan)
**Table S20**	Stratification analysis for the long-term sequelae risk of incident food allergy following COVID-19 diagnosis in the propensity score-matched replication cohort A (Japan)
**Table S21**	The HR with 95% CI for the long-term sequelae risk of incident allergic diseases following mild COVID-19 diagnosis in the propensity score-matched main cohort (South Korea), replication cohort A (Japan), and replication cohort B (UK).
**Table S22**	The HR with 95% CI for the long-term sequelae risk of incident allergic diseases following moderate to severe COVID-19 diagnosis in the propensity score-matched main cohort (South Korea), replication cohort A (Japan), and replication cohort B (UK).
**Table S23**	The HR with 95% CI for the long-term sequelae risk of incident allergic diseases following COVID-19 diagnosis of patients in the propensity score-matched cohorts in main cohort (South Korea), replication cohort A (Japan), and replication cohort B (UK), presenting the *P* value with a two-sided test from Table 2.
**Table S24**	Time attenuation effect on the development of allergic diseases after SARS-CoV-2 infection (model 2; adjusted HR with 95% CI), presenting the *P* value with a two-sided test from Table 3.
**Table S25**	Propensity-score-matched subgroup analysis of HR (95% CI) of allergic diseases following COVID-19 diagnosis stratified by COVID-19 severity, SARS-CoV-2 strain type, and number of vaccinations in main cohort (South Korea), presenting the *P* value with a two-sided test from Table 4.

**Table S26**	The HR with 95% CI for the long-term sequelae risk of incident allergic disease in negative control analysis using non-COVID-19 disease (tympanic membrane perforation) in the propensity score-matched main cohort (South Korea), replication cohort A (Japan), and replication cohort B (UK).
**Table S27**	The HR with 95% CI for the long-term sequelae risk of incident asthma without dyspnea following COVID-19 diagnosis of patients in the propensity score-matched main cohort (South Korea), replication cohort A (Japan), and replication cohort B (UK).
**Table S28**	The HR with 95% CI for the long-term sequelae risk of incident asthma with EDHO following COVID-19 diagnosis of patients in the propensity score-matched main cohort (South Korea).
**Table S29**	The HR with 95% CI for the long-term sequelae risk of incident asthma phenotype (allergic asthma and non-allergic asthma) following COVID-19 diagnosis in the propensity score-matched main cohort (South Korea), replication cohort A (Japan), and replication cohort B (UK).
**Table S30**	The HR with 95% CI for the long-term sequelae risk of incident allergic disease by vaccine dose following COVID-19 diagnosis in the propensity score-matched main cohort (South Korea).
**Table S31**	The HR with 95% CI for the long-term sequelae risk of incident allergic disease by mRNA vaccine dose over time following COVID-19 diagnosis in the propensity score-matched main cohort (South Korea).
**Supplement Material**	Explanation of replication cohorts (Japan and the UK).
**Supplement References**	

Figure S1. Density plot and box plot for before matching and after matching in patients with COVID-19 and non- COVID-19 in main cohort (South Korea: unmatched cohort, n=10,027,506; matched cohort, n=836,164)

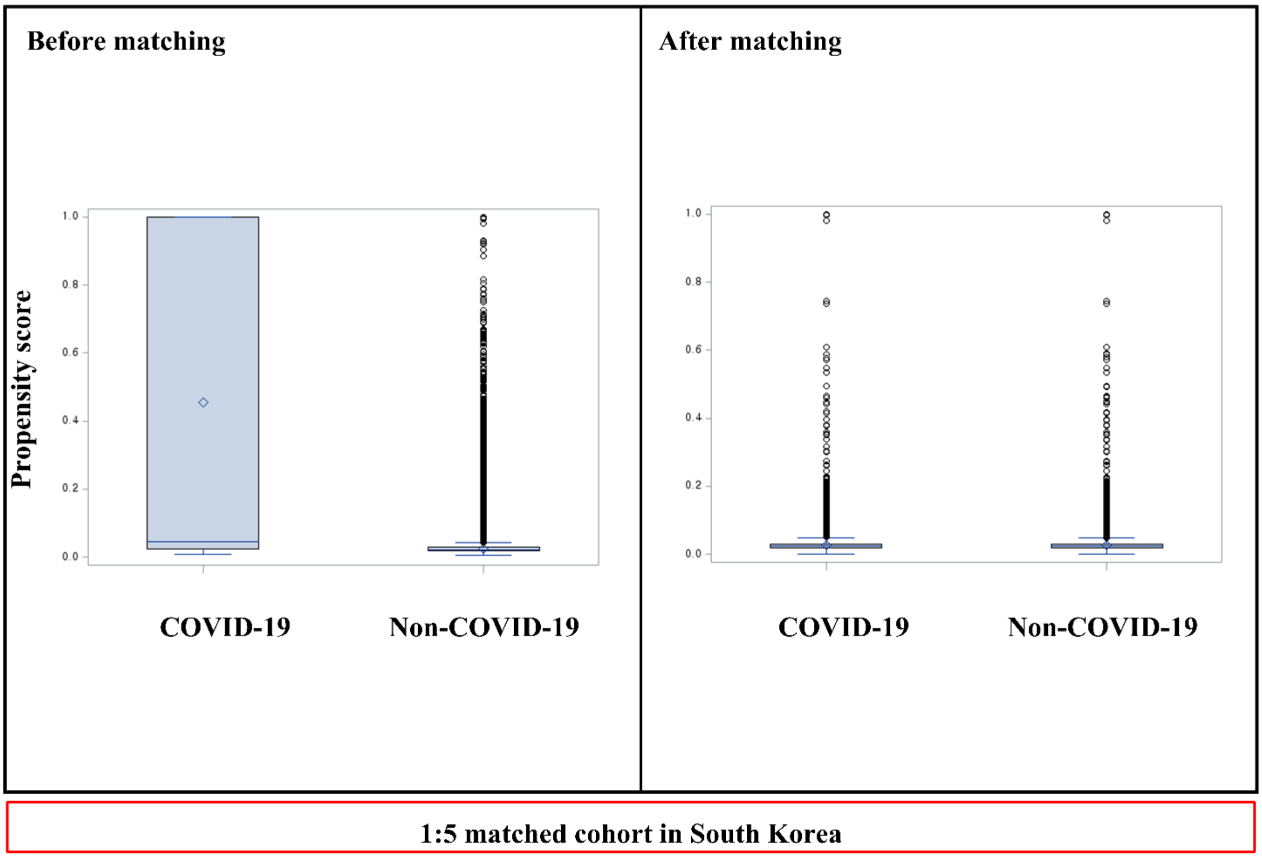

Figure S2. Density plot and box plot for before matching and after matching in patients with COVID-19 and non- COVID-19 in replication cohort A (Japan: unmatched cohort, n=12,218,680; matched cohort, n=2,541,021).

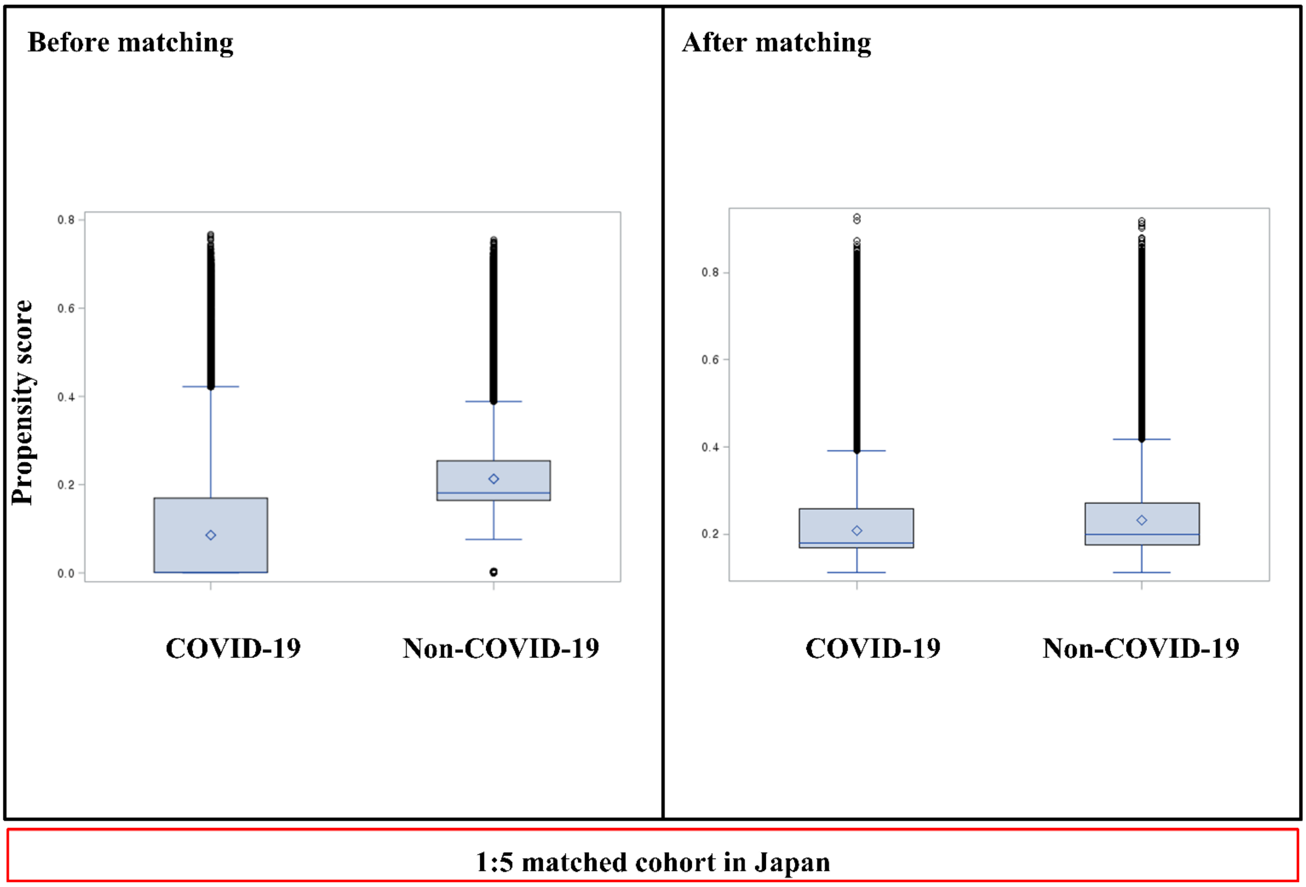

Figure S3. Density plot and box plot for before matching and after matching in patients with COVID-19 and non- COVID-19 in replication cohort B (UK: unmatched cohort, n=468,617; matched cohort, n=325,843)

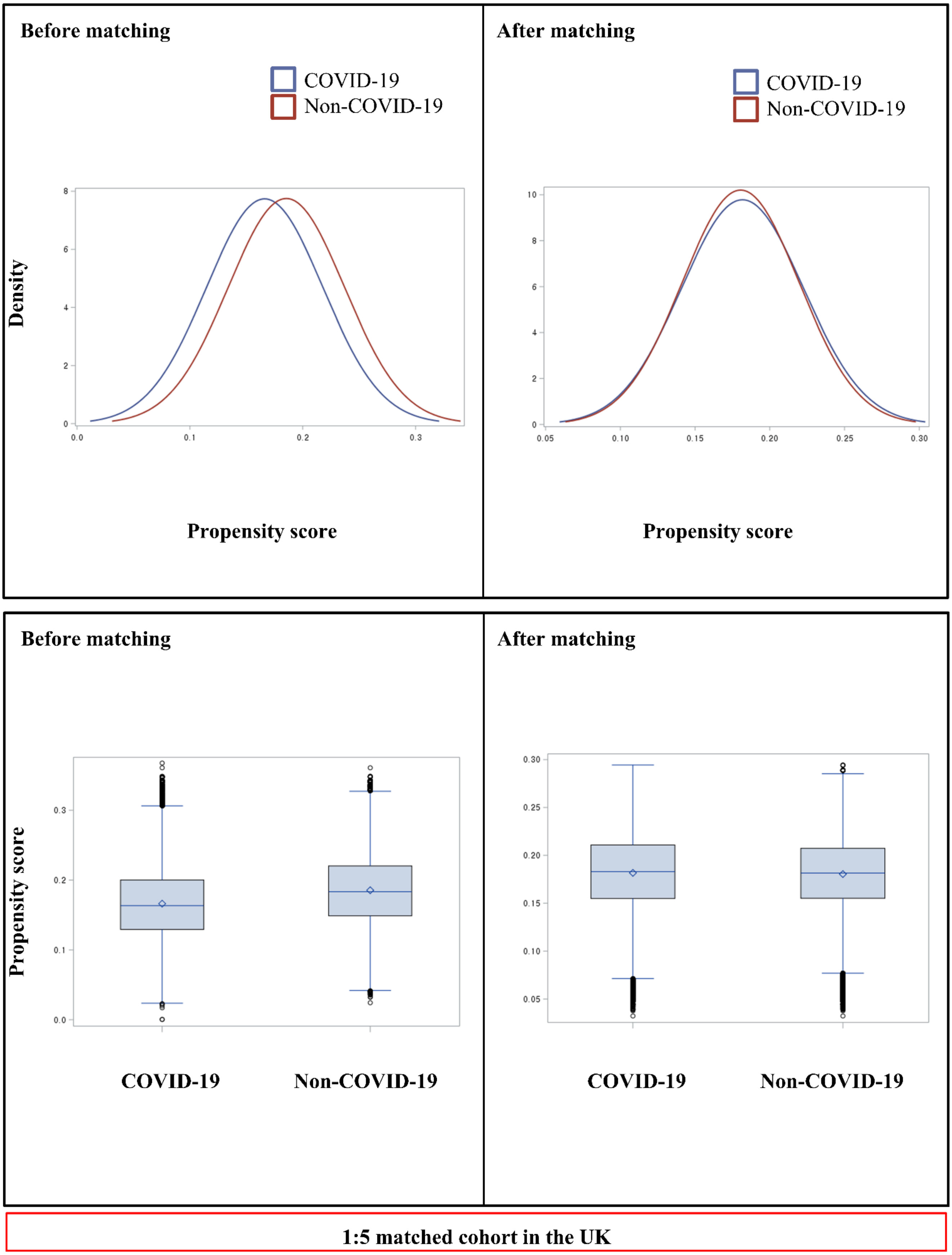

Figure S4. Study flow of cohorts

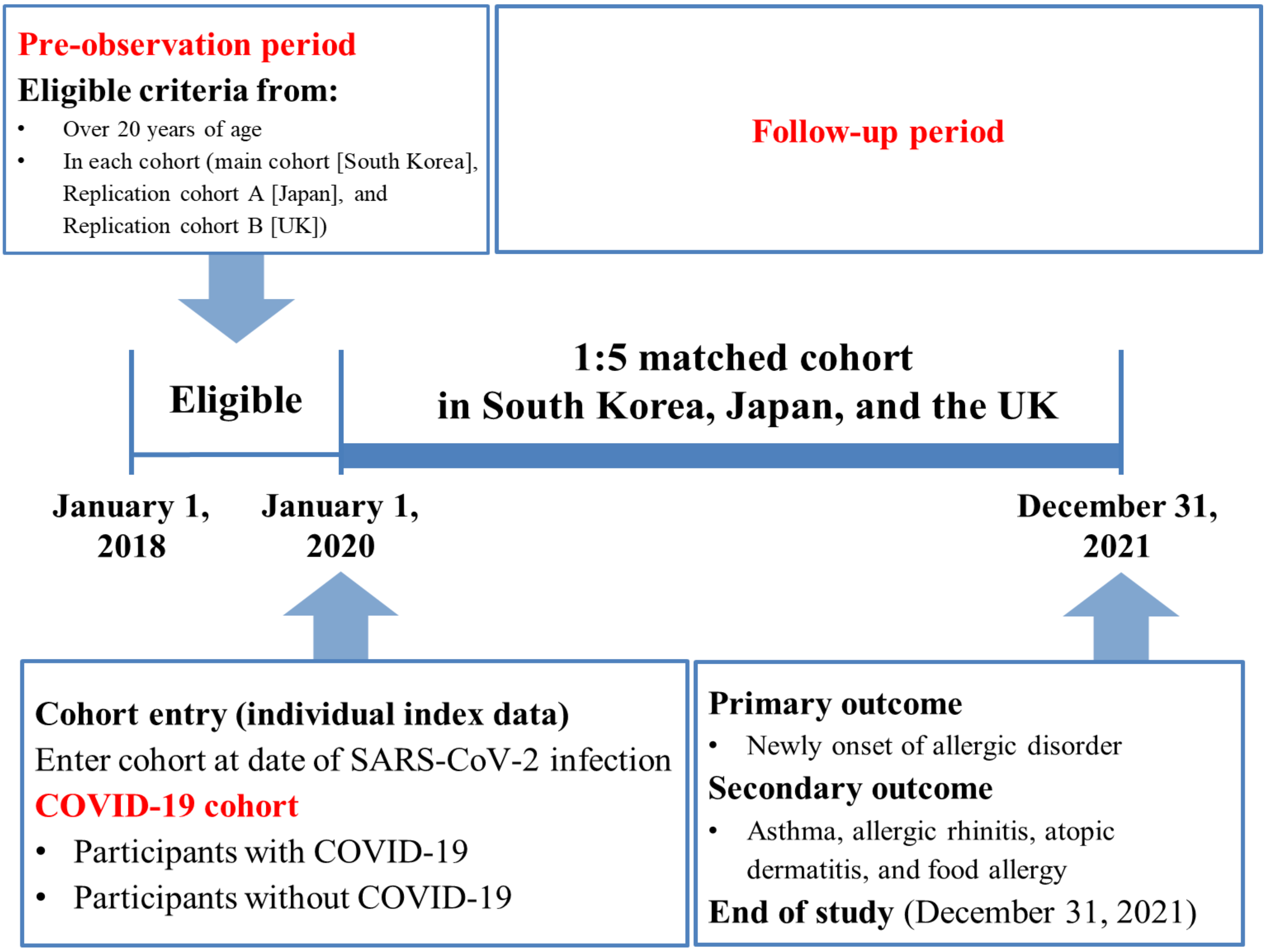

Table S1. Definitions of diseases.

Diseases	ICD-10 codes
Asthma	J45 or J46 with ≥2 claims and the use of asthma related medications (inhaled corticosteroid and systemic corticosteroids, long- and short-acting β2-agonists, and/or leukotriene antagonists)
Allergic asthma	Asthma with at least one of the following conditions: allergic rhinitis, atopic dermatitis, or food allergy.
Non-allergic asthma	Asthma occurring without any of the following conditions: allergic rhinitis, atopic dermatitis, and food allergy.
Allergic rhinitis	J30.1, J30.2, J30.3, or J30.4 with ≥2 claims and the use of allergic rhinitis related medications (antihistamines, corticosteroid nasal sprays, leukotriene antagonists, and/or mast cell stabilizers).
Atopic dermatitis	L20 with ≥2 claims and the use of atopic dermatitis related medications (topical and systemic corticosteroids, calcineurin inhibitors, and systemic immunosuppressants [azathioprine, cyclosporine, mycophenolate mofetil, and/or methotrexate]).
Food allergy	K52.2, K52.3, K52.8, K52.9, L27.2, L23.6, T78.0, T78.1, or Z91.0 with ≥2 claims
History of cardiovascular disease	I00-I02, I05-I09, I50, I51, I52, or I20-I45
History of chronic kidney disease	E10.2, E11.2, E13.2, E14.2, I12.0, M20.0, M31.3, M31.9, M32.1B, N02-N08, N11, N12, N14, N15.8, N15.9, N16.0, N16.2, N16.3, N16.4, N16.8, N18, N19, N26, Q61.2, Q61.3, or Q61.5 with ≥2 claims
History of chronic obstructive pulmonary disease	J44.0, J44.1, or J44.9 with ≥2 claims
Dyspnea	R06.0 with ≥1 claims
Tympanic membrane perforation	H72 with ≥2 claims

ICD-10, International Classification of Diseases, 10th edition.

Table S2. Representative population

Category	
Disease	▪ Exposure: SARS-CoV-2 infection<br>▪ Outcome: allergic diseases<br>1. Allergic diseases are defined as asthma, allergic rhinitis, atopic dermatitis and food allergy.<br>2. We used appropriate ICD-10 codes and related medication for the definition of each disease.
Special considerations related to	
Sex	Women are more likely to have incident allergic diseases than men.
Age	The relationship between age and allergic diseases varies by the subtype of disease. While the incidence of asthma is the highest in the middle-aged, that of food allergy diminished with increasing age.
Socioeconomic status	The incidence rate of allergic diseases is observed to be higher in people of high socioeconomic status.
Ethnicity and country	The allergic diseases affect all races and ethnicities across the world. The incident rate varies widely among ethnicities.
Geography of urban-rural interaction	Environmental factors such as exposure to dust, pollens, and microbial substances are known to influence allergy significantly.
Other considerations	The COVID-19 vaccine, which primes the immune system to respond to infections, may also affect incident allergic diseases.
Overall representativeness of this study	We used the Korean nationwide cohort comprising over 10 million individuals to assess the risks of allergic diseases after the first month of SARS-CoV-2 infection compared to contemporary controls. The Japanese and the UK cohorts were used to enhance the robustness of the main findings. This is the largest analysis to estimate a

	comprehensive set of allergic diseases incidence following SARS-CoV-2 infection. Also, we showed the protective effect of COVID-19 vaccine against incident allergic diseases.

ICD-10, International Classification of Diseases, 10th edition; SARS-CoV-2, severe acute respiratory syndrome coronavirus 2.

Table S3. Baseline characteristics for the full unmatched cohorts of South Korea, Japan, and the UK.

Characteristics	South Korea (n =10,027,506)	Japan (n= 12,218,680)	UK (n= 468,617)
Total, n	10,027,506	12,218,680	468,617
Age, years, mean (SD)	48.40 (13.40)	43.50 (14.70)	71.18 (8.09)
Age, years, n (%)			
20-39	2,756,102 (27.50)	5,298,703 (43.40)	NA
40-59	4,799,784 (47.90)	4,935,219 (40.40)	50,872 (10.86)
≥ 60	2,471,620 (24.70)	1,984,758 (16.20)	417,745 (89.14)
Sex, n (%)			
Male	5,026,885 (50.10)	6,334,565 (51.80)	209,472 (44.70)
Female	5,000,621 (49.90)	5,884,115 (48.20)	259,145 (55.30)
Region of residence, n (%)			
Rural	4,460,562 (44.48)	NA	65,206 (13.90)
Urban	5,566,944 (55.52)	NA	403,411 (86.10)

SD, standard deviation

Table S4. Baseline characteristics for 1:5 propensity score-matched cohort in the replication cohort A (Japan).

Covariates	1:5 matched cohort (n= 2,541,021)			
	Total	COVID-19 (n=542,497)	Non-COVID-19 (n= 1,998,524)	SMD
Age, years, mean (SD)	46.91 (11.41)	44.95 (11.96)	47.44 (11.20)	0.215
Age, years, n (%)				0.221
20–39	677,584 (26.67)	187,828 (34.62)	489,756 (24.51)	
40–59	1,527,989 (60.13)	289,616 (53.39)	1,238,373 (61.96)	
≥ 60	335,448 (13.20)	65,053 (11.99)	270,395 (13.53)	
Sex, n (%)				0.030
Male	1,712,733 (67.40)	359,753 (66.31)	1,352,980 (67.70)	
Female	828,288 (32.60)	182,744 (33.69)	645,544 (32.30)	
Charlson comorbidity index, n (%)				0.180
0	2,497,253 (98.28)	521,111 (96.06)	1,976,142 (98.88)	
1	13,724 (0.54)	6,583 (1.21)	7,141 (0.36)	
≥2	30,044 (1.18)	14,803 (2.73)	15,241 (0.76)	
History of cardiovascular disease, n (%)	237,077 (9.33)	72,945 (13.45)	164,132 (8.21)	0.169
History of chronic kidney disease, n (%)	111,533 (4.39)	28,843 (5.32)	82,690 (4.14)	0.056
History of chronic obstructive pulmonary disease, n (%)	5,964 (0.23)	2,407 (0.44)	3,557 (0.18)	0.048
History of medication use for diabetes, n (%)	67,783 (2.67)	14,605 (2.69)	53,178 (2.66)	0.002
History of medication use for hyperlipidemia, n (%)	155,190 (6.11)	31,890 (5.88)	123,300 (6.17)	0.012
History of medication use for hypertension, n (%)	204,823 (8.06)	43,409 (8.00)	161,414 (8.08)	0.003
Body mass index, kg/m^2, n (%)				0.034
Underweight (<18.5)	189,039 (7.44)	42,662 (7.86)	146,377 (7.32)	
Normal (18.5–23.0)	1,103,232 (43.42)	238,313 (43.93)	864,919 (43.28)	
Overweight (23.0–25.0)	500,160 (19.68)	103,239 (19.03)	396,921 (19.86)	

Obese (≥25.0)	736,406 (28.98)	156,101 (28.77)	580,305 (29.04)	
Unknown	12,184 (0.48)	2,182 (0.40)	10,002 (0.50)	
Blood pressure, n (%)				0.056
SBP <140 mmHg and DBP <90 mmHg	2,130,467 (83.84)	463,355 (85.41)	1,667,112 (83.42)	
SBP ≥140 mmHg or DBP ≥90 mmHg	397,463 (15.64)	76,778 (14.15)	320,685 (16.05)	
Unknown	13,091 (0.52)	2,364 (0.44)	10,727 (0.54)	
Fasting blood glucose, mg/dL, n (%)				0.055
<100	1,710,497 (67.32)	372,569 (68.68)	1,337,928 (66.95)	
≥100	616,724 (24.27)	121,909 (22.47)	494,815 (24.76)	
Unknown	213,800 (8.41)	48,019 (8.85)	165,781 (8.30)	
Serum total cholesterol, mg/dL, n (%)				0.084
<200	1,111,656 (43.75)	254,222 (46.86)	857,434 (42.90)	
200−240	960,246 (37.79)	195,032 (35.95)	765,214 (38.29)	
≥240	440,673 (17.34)	86,712 (15.98)	353,961 (17.71)	
Unknown	28,446 (1.12)	6,531 (1.20)	21,915 (1.10)	
Glomerular filtration rate, mL/min/1.73 m^2, n (%)				0.026
<60	63,408 (2.48)	15,244 (2.81)	48,164 (2.41)	
60−90	1,080,930 (42.54)	231,158 (42.61)	849,772 (42.52)	
≥90	1,362,670 (53.64)	288,663 (53.21)	1,074,007 (53.74)	
Unknown	34,013 (1.34)	7,432 (1.37)	26,581 (1.33)	
Smoking status, n (%)				0.023
Current smoker	675,763 (26.59)	140,478 (25.89)	535,285 (26.78)	
Non-smoker	1,783,816 (70.20)	383,597 (70.71)	1,400,219 (70.06)	
Unknown	81,442 (3.21)	18,422 (3.40)	63,020 (3.15)	
Alcoholic drinks, days per week, n (%)				0.044
<1	587,680 (23.13)	118,476 (21.84)	469,204 (23.48)	
1–2	826,498 (32.53)	182,406 (33.62)	644,092 (32.23)	
3–4	982,194 (38.65)	207,717 (38.29)	774,477 (38.75)	

≥5	144,649 (5.69)	33,898 (6.25)	110,751 (5.54)	
Unknown	587,680 (23.13)	118,476 (21.84)	469,204 (23.48)	
Aerobic physical activity, n (%)				0.043
Sufficient	546,224 (21.50)	110,731 (20.41)	435,493 (21.79)	
Insufficient	1,821,694 (71.69)	391,479 (72.16)	1,430,215 (71.56)	
Unknown	173,103 (6.81)	40,287 (7.43)	132,816 (6.65)	

DBP, diastolic blood pressure; SBP, systolic blood pressure; SD, standard deviation; SMD, standardized mean difference.

Table S5. Baseline characteristics for 1:5 propensity score-matched cohort in the replication cohort B (UK).

Covariates	1:5 matched cohort (n=325,843)			
	Total	COVID-19 (n=76,894)	Non-COVID-19 (n=248,949)	SMD
Age, years, mean (SD)	71.08 (7.87)	71.33 (7.58)	71.00 (7.95)	0.048
Age, years, n (%)				0.048
40–59	30,577 (9.38)	6,726 (8.75)	23,851 (9.58)	
≥ 60	295,266 (90.62)	70,168 (91.25)	225,098 (90.42)	
Sex, n (%)				0.043
Male	186,007 (57.08)	31,764 (41.31)	108,072 (43.41)	
Female	139,836 (42.92)	45,130 (58.69)	140,877 (56.59)	
Region of residence				0.014
Urban	277,445 (85.15)	65,179 (84.76)	212,266 (85.26)	
Rural	48,398 (14.85)	11,715 (15.24)	36,683 (14.74)	
Household income, n (%)				0.167
Level1 (<₤18,000)	53,572 (16.44)	10,311 (13.41)	43,261 (17.38)	
Level2 (₤18,000-₤30,999)	70,524 (21.64)	16,209 (21.08)	54,315 (21.82)	
Level3 (₤31,000-₤51,999)	76,821 (23.58)	19,028 (24.75)	57,793 (23.21)	
Level4 (₤52,000-₤100,000)	62,610 (19.21)	16,916 (22.00)	45,694 (18.35)	
Level5 (>₤100,000)	17,314 (5.31)	5,177 (6.73)	12,137 (4.88)	
Unknown	45,002 (13.81)	9,253 (12.03)	35,749 (14.36)	
Townsend deprivation index, mean (SD)	-1.43 (3.02)	-1.73 (2.83)	-1.35 (3.06)	0.026
Townsend deprivation index tertile				0.020
T1 (last deprived)	118,547 (36.38)	28,477 (37.03)	90,070 (36.18)	

T2	112,207 (34.44)	26,375 (34.30)	85,832 (34.48)	
T3 (most deprived)	94,822 (29.10)	21,973 (28.58)	72,849 (29.26)	
Unknown	267 (0.08)	69 (0.09)	198 (0.08)	
Charlson comorbidity index, n (%)				0.064
0	311,545 (95.61)	72,715 (94.57)	238,830 (95.94)	
1	6,762 (2.08)	2,008 (2.61)	4,754 (1.91)	
≥2	7,536 (2.31)	2,171 (2.82)	5,365 (2.16)	
Race, n (%)				<0.001
White	313,685 (96.27)	74,072 (96.33)	239,613 (96.25)	
Mixed	2,495 (0.77)	599 (0.78)	1,896 (0.76)	
Asian	3,874 (1.19)	915 (1.19)	2,959 (1.19)	
Black	2,710 (0.83)	589 (0.77)	2,121 (0.85)	
Others	1,957 (0.60)	449 (0.58)	1,508 (0.61)	
Unknown	1,122 (0.34)	270 (0.35)	852 (0.34)	
Education levels, years, n (%)				0.012
≤10	176 (0.05)	44 (0.06)	132 (0.05)	
11-12	9,090 (2.79)	2,261 (2.94)	6,829 (2.74)	
>12	314,338 (96.47)	74,063 (96.32)	240,275 (96.52)	
Unknown	2,239 (0.69)	526 (0.68)	1,713 (0.69)	
History of cardiovascular disease, n (%)	32,179 (9.88)	8,264 (10.75)	23,915 (9.61)	0.038
History of chronic kidney disease, n (%)	17,274 (5.30)	4,849 (6.31)	12,425 (4.99)	0.057
History of chronic obstructive pulmonary disease, n (%)	6,330 (1.94)	2,051 (2.67)	4,279 (1.72)	0.065
History of medication use for diabetes, n (%)	586 (0.18)	148 (0.19)	438 (0.18)	0.004
History of medication use for hyperlipidemia, n (%)	10,609 (3.26)	2,551 (3.32)	8,058 (3.24)	0.005
History of medication use for hypertension, n (%)	30,180 (9.26)	7,164 (9.32)	23,016 (9.25)	0.003
Body mass index, kg/m^2, n (%)				0.031
Normal (<25.0)	121,657 (37.34)	29,580 (38.47)	92,077 (36.99)	
Overweight (25.0–30.0)	140,093 (42.99)	32,341 (42.06)	107,752 (43.28)	
Obese (≥30.0)	63,108 (19.37)	14,740 (19.17)	48,368 (19.43)	
Unknown	985 (0.30)	233 (0.30)	752 (0.30)	

Blood pressure, n (%)				<0.001
SBP <140 mmHg and DBP <90 mmHg	167 (0.05)	42 (0.05)	125 (0.05)	
SBP ≥140 mmHg or DBP ≥90 mmHg	308,228 (94.59)	72,857 (94.75)	235,371 (94.55)	
Unknown	17,448 (5.35)	3,995 (5.20)	13,453 (5.40)	
Fasting blood glucose, mg/dL, n (%)				0.010
<100	241,863 (74.23)	56,841 (73.92)	185,022 (74.32)	
≥100	40,329 (12.38)	9688 (12.60)	30,641 (12.31)	
Unknown	43,651 (13.4)	10,365 (13.48)	33,286 (13.37)	
Smoking status, n (%)				<0.001
Current smoker	187,176 (57.44)	44,105 (57.36)	143,071 (57.47)	
Non-smoker	137,877 (42.31)	32,598 (42.39)	105,279 (42.29)	
Unknown	790 (0.24)	191 (0.25)	599 (0.24)	
Alcoholic drinks, days per week, n (%)				0.019
Every day	50,535 (15.51)	11,813 (15.36)	38,722 (15.55)	
Sometimes	120,935 (37.11)	28,101 (36.55)	92,834 (37.29)	
Rarely	154,024 (47.27)	36,901 (47.99)	117,123 (47.05)	
Unknown	349 (0.11)	79 (0.10)	270 (0.11)	
Aerobic physical activity, n (%)				0.013
Low	94,783 (29.09)	22,269 (28.96)	72,514 (29.13)	
Moderate	113,035 (23.07)	27,018 (35.14)	86,017 (34.55)	
High	108,836 (34.69)	25,414 (33.05)	83,422 (33.51)	
Unknown	9189 (33.4)	2193 (2.85)	6996 (2.81)	

DBP, diastolic blood pressure; SBP, systolic blood pressure; SD, standard deviation; SMD, standardized mean difference.

Table S6. Statistical analysis and justification.

Sensitivity analysis (1 to 5)	Cohort	Justification
1. Incident allergic diseases and its subtypes (asthma, allergic rhinitis, atopic dermatitis, and food allergy) after SARS-CoV-2 versus contemporary control	1. Main cohort after 1:5 propensity score matching<br>2. Replication cohort A after 1:5 propensity score matching<br>3. Replication cohort B after 1:5 propensity score matching	- Main results (South Korea) with replications A (Japan) and B (UK)<br>- To investigate the association of subsequent all-cause allergic diseases following COVID-19<br><br>- **Model (main cohort):** adjusted for age (20–39, 40–59, and ≥60 years); sex; household income (low income, middle income, and high income); region of residence (urban and rural); Charlson comorbidity index (0, 1, and ≥2); BMI (underweight [<18.5 kg/m^2], normal [18.5–23.0 kg/m^2], overweight [23.0–25.0 kg/m^2], obese [≥25.0 kg/m^2], and unknown); blood pressure (systolic blood pressure <140 mmHg and diastolic blood pressure <90 mmHg, systolic blood pressure ≥140 mmHg or diastolic blood pressure ≥90 mmHg, and unknown); fasting blood glucose (<100, ≥100 mg/dL, and unknown); serum total cholesterol (<200, 200–240, ≥240 mg/dL, and unknown); glomerular filtration rate (<60, 60−90, ≥90 mL/min/1.73 m^2, and unknown); smoking status (non-, ex-, current smoker, and unknown); alcoholic drinks (<1, 1–2, 3−4, ≥5 days per week, and unknown); aerobic physical activity (sufficient, insufficient, and unknown); previous history of cardiovascular disease, chronic kidney disease, and chronic obstructive pulmonary disease; history of medication use for diabetes mellitus, dyslipidemia, and hypertension; and missing indicators (BMI missing indicator [yes or no], blood pressure missing indicator [yes or no], fasting blood glucose missing indicator [yes or no], serum total cholesterol missing indicator [yes or no], glomerular filtration rate missing indicator [yes or no], smoking status missing indicator [yes or no], alcoholic drinks missing indicator [yes or no], and aerobic physical activity missing indicator [yes or no]).<br>- **Model (replication cohort A):** adjusted for age (20–39, 40–59, and ≥60 years); sex; Charlson comorbidity index (0, 1, and ≥ 2); BMI (underweight [<18.5 kg/m^2], normal [18.5–23.0 kg/m^2], overweight [23.0–25.0 kg/m^2], obese [≥25.0 kg/m^2], and unknown); blood pressure (systolic blood pressure <140 mmHg and diastolic blood pressure <90 mmHg, systolic blood pressure ≥140 mmHg or diastolic blood pressure ≥90 mmHg, and

unknown); fasting blood glucose (<100, ≥100 mg/dL, and unknown); serum total cholesterol (<200, 200–240, ≥240 mg/dL, and unknown); glomerular filtration rate (<60, 60–90, ≥90 mL/min/1.73 m^2, and unknown); smoking status (non- and current smoker, and unknown); alcoholic drinks (<1, 1–2, 3–4, ≥5 days per week, and unknown); aerobic physical activity (sufficient, insufficient, and unknown); previous history of cardiovascular disease, chronic kidney disease, and chronic obstructive pulmonary disease; history of medication use for diabetes mellitus, dyslipidemia, and hypertension; and missing indicators (BMI missing indicator [yes or no], blood pressure missing indicator [yes or no], fasting blood glucose missing indicator [yes or no], serum total cholesterol missing indicator [yes or no], glomerular filtration rate missing indicator [yes or no], smoking status missing indicator [yes or no], alcoholic drinks missing indicator [yes or no], and aerobic physical activity missing indicator [yes or no]).

- **Model (replication cohort B):** adjusted for age (20–39, 40–59, and ≥ 60 years); sex; household income (<£18,000, £18,000–£30,999, £31,000–£51,999, £52,000–£100,000, >£100,000), and unknown); region of residence (urban and rural); townsend deprivation index (T1 [last deprived], T2, T3 [most deprived], and unknown); race (white, mixed, Asian, black, others, and unknown); Charlson comorbidity index (0, 1, and ≥ 2); BMI (normal [<25.0 kg/m^2], overweight [25.0–30.0 kg/m^2], obese [≥ 30.0 kg/m^2], and unknown); education levels (≤10, 11-12, >12, and unknown); blood pressure (systolic blood pressure < 140 mmHg and diastolic blood pressure < 90 mmHg, systolic blood pressure ≥ 140 mmHg or diastolic blood pressure ≥ 90 mmHg, and unknown); fasting blood glucose (<100, ≥100 mg/dL, and unknown); smoking status (non- and current smoker, and unknown); alcohol consumption (every day, sometimes, rarely days per week, and unknown); aerobic physical activity (low, moderate, high, and unknown); previous history of cardiovascular disease, chronic kidney disease, and chronic obstructive pulmonary disease; history of medication use for diabetes mellitus, dyslipidemia, and hypertension; and missing indicators (household income missing indicator [yes or no], townsend deprivation index missing indicator [yes or no], race missing indicator [yes or no], education levels [yes or no], obesity missing indicator [yes or no], blood pressure missing indicator [yes or no], fasting blood glucose missing indicator [yes or no], serum total cholesterol missing indicator [yes or no], glomerular filtration rate missing indicator [yes or no], smoking status missing indicator [yes or no],

		alcoholic drinks missing indicator [yes or no], and aerobic physical activity missing indicator [yes or no]).
2. Attenuation time effect of allergic diseases development after COVID-19	1. Main cohort after 1:5 propensity score matching<br>2. Replication cohort A after 1:5 propensity score matching	- We investigated the time attenuation effect of allergic diseases development after SARS-CoV-2 infection (<3, 3 to 6, and ≥6 months).
3. Subgroup analysis of **allergic diseases** following COVID-19 diagnosis stratified by vaccination, COVID-19 severity, and SARS-CoV-2 strain type	1. Main cohort after 1:5 propensity score matching	- We performed the subgroup analysis of **allergic diseases** following COVID-19 diagnosis stratified by vaccination (once and twice or more), COVID-19 severity (mild and moderate-to-severe), and SARS-CoV-2 strain type (original and delta).
**4.** Subgroup analysis of **asthma** following COVID-19 diagnosis stratified by vaccination, COVID-19 severity, and SARS-CoV-2 strain type	1. Main cohort after 1:5 propensity score matching	- We performed the subgroup analysis of **asthma** following COVID-19 diagnosis stratified by vaccination (once and twice or more), COVID-19 severity (mild and moderate-to-severe), and SARS-CoV-2 strain type (original and delta).
5. Subgroup analysis of **allergic rhinitis** following COVID-19 diagnosis stratified by vaccination, COVID-19 severity, and SARS-CoV-2 strain type	1. Main cohort after 1:5 propensity score matching	- We performed the subgroup analysis of **allergic rhinitis** following COVID-19 diagnosis stratified by vaccination (once and twice or more), COVID-19 severity (mild and moderate-to-severe), and SARS-CoV-2 strain type (original and delta).
6. Subgroup analysis of **atopic dermatitis** following COVID-19 diagnosis stratified by vaccination, COVID-19 severity, and SARS-CoV-2 strain type	1. Main cohort after 1:5 propensity score matching	- We performed the subgroup analysis of **atopic dermatitis** following COVID-19 diagnosis stratified by vaccination (once and twice or more), COVID-19 severity (mild and moderate-to-severe), and SARS-CoV-2 strain type (original and delta).
7. Subgroup analysis of **food allergy** following COVID-19 diagnosis stratified by vaccination, COVID-19 severity, and SARS-CoV-2 strain type	1. Main cohort after 1:5 propensity score matching	- We performed the subgroup analysis of **food allergy** following COVID-19 diagnosis stratified by vaccination (once and twice or more), COVID-19 severity (mild and moderate-to-severe), and SARS-CoV-2 strain type (original and delta).
8. Stratification analysis of the risk of incident **allergic diseases**	1. Main cohort after 1:5 propensity score matching<br>2. Replication cohort A after 1:5 propensity score matching	- To investigate unexpected mediated effect, we performed the stratification analysis of **allergic diseases** following COVID-19 diagnosis.<br>- **Stratification analysis (main cohort):** Age (20–39, 40–59, and ≥60 years); sex; household income (low income, middle income, and high income); region of residence (urban and rural); Charlson comorbidity index (0, 1, and ≥2); BMI (underweight [<18.5

		kg/m^2], normal [18.5–23.0 kg/m^2], overweight [23.0–25.0 kg/m^2], and obese [≥25.0 kg/m^2]); smoking status (non-, ex-, and current smoker); alcoholic drinks (<1, 1–2, 3–4, and ≥5 days per week); and aerobic physical activity (sufficient and insufficient).<br>- **Stratification analysis (replication cohort A):** age (20–39, 40–59, and ≥60 years); sex; Charlson comorbidity index (0, 1, and ≥ 2); BMI (underweight [<18.5 kg/m^2], normal [18.5–23.0 kg/m^2], overweight [23.0–25.0 kg/m^2], and obese [≥25.0 kg/m^2]); alcoholic drinks (drinks; <1, 1–2, 3–4, and ≥5 days per week); and aerobic physical activity (sufficient and insufficient).
9. Stratification analysis of the risk of incident **asthma, allergic rhinitis, atopic dermatitis, and food allergy**	1. Main cohort after 1:5 propensity score matching<br>2. Replication cohort A after 1:5 propensity score matching	- To investigate unexpected mediated effect, we performed the stratification analysis of **asthma, allergic rhinitis, atopic dermatitis, and food allergy** following COVID-19 diagnosis.<br>- **Stratification analysis (main cohort):** Age (20–39, 40–59, and ≥60 years); sex; household income (low income, middle income, and high income); region of residence (urban and rural); Charlson comorbidity index (0, 1, and ≥2); BMI (underweight [<18.5 kg/m^2], normal [18.5–23.0 kg/m^2], overweight [23.0–25.0 kg/m^2], and obese [≥25.0 kg/m^2]); smoking status (non-, ex-, and current smoker); alcoholic drinks (<1, 1–2, 3–4, and ≥5 days per week); and aerobic physical activity (sufficient and insufficient).<br>- **Stratification analysis (replication cohort A):** age (20–39, 40–59, and ≥60 years); sex; Charlson comorbidity index (0, 1, and ≥ 2); BMI (underweight [<18.5 kg/m^2], normal [18.5–23.0 kg/m^2], overweight [23.0–25.0 kg/m^2], and obese [≥25.0 kg/m^2]); alcoholic drinks (drinks; <1, 1–2, 3–4, and ≥5 days per week); and aerobic physical activity (sufficient and insufficient).
10. **Negative control analysis** of the risk of incident tympanic membrane perforation disease.	1. Main cohort after 1:5 propensity score matching<br>2. Replication cohort A after 1:5 propensity score matching<br>3. Replication cohort B after 1:5 propensity score matching	- To verify the validity of our findings and identify potential misclassification bias, we conducted a negative control analysis of **tympanic membrane perforation disease** following the COVID-19 diagnosis.
11. Sensitivity analysis of the risk of incident **asthma without dyspnea**	1. Main cohort after 1:5 propensity score matching	- To reduce misclassification bias due to dyspnea, we performed an analysis excluding symptoms of dyspnea in asthma cases following the COVID-19 diagnosis.

	2. Replication cohort A after 1:5 propensity score matching<br>3. Replication cohort B after 1:5 propensity score matching	
12. Sensitivity analysis of the risk of incident **asthma with only EDHO**	1. Main cohort after 1:5 propensity score matching	- We conducted analyses on cases diagnosed with asthma following the COVID-19 diagnosis, considering those with a history of emergency department visits or hospitalization.
13. Sensitivity analysis of the risk of incident **asthma phenotype (allergic and non-allergic asthma)**	1. Main cohort after 1:5 propensity score matching<br>2. Replication cohort A after 1:5 propensity score matching<br>3. Replication cohort B after 1:5 propensity score matching	- In order to precisely classify patients with asthma following the COVID-19 diagnosis, allergic asthma and non-allergic asthma were compared as distinct groups.<br>- **Allergic asthma:** asthma with at least one of the following conditions: allergic rhinitis, atopic dermatitis, or food allergy.<br>- **Non-allergic asthma:** asthma occurring without any of the following conditions: allergic rhinitis, atopic dermatitis, and food allergy.
13. Sensitivity analysis of the risk of incident allergic diseases following **COVID-19 severity status (mild and moderate to severe)**	1. Main cohort after 1:5 propensity score matching<br>2. Replication cohort A after 1:5 propensity score matching<br>3. Replication cohort B after 1:5 propensity score matching	- To investigate the association of subsequent all-cause allergic diseases following COVID-19 by severity status.
14. Sensitivity analysis of the risk of incident **allergic disease by vaccine dose and time attenuation effect**	1. Main cohort after 1:5 propensity score matching	- For understanding the long-term immune protection provided by the COVID-19 vaccine and its effectiveness extent, we performed vaccine dose analysis and time attenuation analysis.<br>- **Vaccine dose analysis:** we aimed to investigate the onset of allergic diseases in relation to SARS-CoV-2 infection status among individuals with the same number of vaccine doses **(without vaccination, vaccination 1 time, and vaccination ≥2 times)**.<br>- **Time attenuation analysis:** to examine the potential impact over time, we investigated the time attenuation effect of the vaccine at **30 days after the vaccination**.

BMI, body mass index; CCI, Charlson comorbidity index; EDHO, history of emergency department visits or hospitalizations; SARS-CoV-2, severe acute respiratory syndrome coronavirus 2.

Table S7. The propensity-score-matched subgroup analysis of HR (95% CI) of **asthma** following COVID-19 diagnosis stratified by COVID-19 severity, SARS-CoV-2 strain type, and number of vaccinations in the main cohort (South Korea; n=836,164).

Factors	Group	Exposure	Events/total number (%)	HR (95% CI)	
				Model 1 [a]	Model 2 [b]
COVID-19 severity	Total	Non-infected control	230/688,340 (0.03)	1.00 (reference)	1.00 (reference)
		Mild COVID-19	80/127,641 (0.06)	**1.99 (1.55 to 2.57)**	**2.08 (1.61 to 2.68)**
		Moderate to severe COVID-19	30/20,183 (0.15)	**3.60 (2.45 to 5.29)**	**2.91 (1.97 to 4.31)**
Strain type (original)	Total	Non-infected control at the same index date‡	182/220,904 (0.08)	1.00 (reference)	1.00 (reference)
		Original SARS-CoV-2 infection	79/46,900 (0.17)	**2.08 (1.59 to 2.70)**	**2.07 (1.59 to 2.69)**
Strain type (delta)	Total	Non-infected control at the same index date‡	48/467,436 (0.01)	1.00 (reference)	1.00 (reference)
		Delta SARS-CoV-2 infection	31/100,924 (17.76)	**3.01 (1.91 to 4.72)**	**2.97 (1.89 to 4.66)**
Number of SARS-CoV-2 vaccinations	Patients with COVID-19	Non-infected control	230/688,340 (0.03)	1.00 (reference)	1.00 (reference)
		Without vaccination	85/68,456 (0.12)	**2.30 (1.79 to 2.95)**	**2.27 (1.77 to 2.91)**
		Vaccination 1 time	13/14,125 (0.09)	**2.87 (1.64 to 5.04)**	**2.95 (1.68 to 5.18)**
		Vaccination ≥2 times	12/65,243 (0.02)	1.73 (0.95 to 3.12)	1.73 (0.96 to 3.14)

HR, hazard ratio; CI, confidence interval; SARS-CoV-2, severe acute respiratory syndrome coronavirus

The data in bold indicate significant differences (*P* < 0.05).

[a] **Model 1**: Adjusted for age (20–39, 40–59, and ≥60 years) and sex.

[b] **Model 2**: Adjusted for age (20–39, 40–59, and ≥60 years); sex; household income (low income, middle income, and high income); region of residence (urban and rural); Charlson comorbidity index (0, 1, and ≥2); BMI (underweight [<18.5 kg/m^2], normal [18.5–23.0 kg/m^2], overweight [23.0–25.0 kg/m^2], obese [≥25.0 kg/m^2], and unknown); blood pressure (systolic blood pressure <140 mmHg and diastolic blood pressure <90 mmHg, systolic blood pressure ≥140 mmHg or diastolic blood pressure ≥90 mmHg, and unknown); fasting blood glucose (<100, ≥100 mg/dL, and unknown); serum total cholesterol (<200, 200–240, ≥240

mg/dL, and unknown); glomerular filtration rate (<60, 60−90, ≥90 mL/min/1.73 m^2, and unknown); smoking status (non-, ex-, current smoker, and unknown); alcoholic drinks (<1, 1–2, 3–4, ≥5 days per week, and unknown); aerobic physical activity (sufficient, insufficient, and unknown); previous history of cardiovascular disease, chronic kidney disease, and chronic obstructive pulmonary disease; history of medication use for diabetes mellitus, dyslipidemia, and hypertension; and missing indicators (BMI missing indicator [yes or no], blood pressure missing indicator [yes or no], fasting blood glucose missing indicator [yes or no], serum total cholesterol missing indicator [yes or no], glomerular filtration rate missing indicator [yes or no], smoking status missing indicator [yes or no], alcoholic drinks missing indicator [yes or no], and aerobic physical activity missing indicator [yes or no]).

‡ Comparators defined only 1:5 matched comparators in each patient group at the same index date to reduce immortal bias.

Table S8. The propensity-score-matched subgroup analysis of HR (95% CI) of **allergic rhinitis** following COVID-19 diagnosis stratified by COVID-19 severity, SARS-CoV-2 strain type, and number of vaccinations in the main cohort (South Korea; n=836,164).

Factors	Group	Exposure	Events/total number (%)	HR (95% CI)	
				Model 1 [a]	Model 2 [b]
COVID-19 severity	Total	Non-infected control	4,066/688,340 (0.59)	1.00 (reference)	1.00 (reference)
		Mild COVID-19	847/127,641 (0.66)	**1.17 (1.09 to 1.26)**	**1.18 (1.10 to 1.27)**
		Moderate to severe COVID-19	207/20,183 (1.03)	**1.54 (1.34 to 1.77)**	**1.50 (1.30 to 1.73)**
Strain type (original)	Total	Non-infected control at the same index date‡	182/220,904 (82.49)	1.00 (reference)	1.00 (reference)
		Original SARS-CoV-2 infection	79/46,900 (0.17)	**2.08 (1.59 to 2.70)**	**2.07 (1.59 to 2.69)**
Strain type (delta)	Total	Non-infected control at the same index date‡	48/467,436 (0.01)	1.00 (reference)	1.00 (reference)
		Delta SARS-CoV-2 infection	31/100,924 (0.03)	**3.01 (1.91 to 4.72)**	**2.97 (1.89 to 4.66)**
Number of SARS-CoV-2 vaccinations	Patients with COVID-19	Non-infected control	4,066/688,340 (0.59)	1.00 (reference)	1.00 (reference)
		Without vaccination	842/68,456 (1.23)	**1.28 (1.18 to 1.38)**	**1.28 (1.19 to 1.38)**
		Vaccination 1 time	107/14,125 (0.76)	**1.43 (1.18 to 1.74)**	**1.44 (1.18 to 1.74)**
		Vaccination ≥2 times	105/65,243 (0.16)	0.86 (0.71 to 1.04)	0.86 (0.70 to 1.04)

BMI, body mass index; CI, confidence interval; HR, hazard ratio; SARS-CoV-2, severe acute respiratory syndrome coronavirus.

The data in bold indicate significant differences (*P* < 0.05).

[a] **Model 1**: Adjusted for age (20–39, 40–59, and ≥60 years) and sex.

[b] **Model 2**: Adjusted for age (20–39, 40–59, and ≥60 years); sex; household income (low income, middle income, and high income); region of residence (urban and rural); Charlson comorbidity index (0, 1, and ≥2); BMI (underweight [<18.5 kg/m^2], normal [18.5–23.0 kg/m^2], overweight [23.0–25.0 kg/m^2], obese [≥25.0 kg/m^2], and unknown); blood pressure (systolic blood pressure <140 mmHg and diastolic blood pressure <90 mmHg, systolic blood pressure ≥140 mmHg or diastolic blood pressure ≥90 mmHg, and unknown); fasting blood glucose (<100, ≥100 mg/dL, and unknown); serum total cholesterol (<200, 200–240, ≥240

mg/dL, and unknown); glomerular filtration rate (<60, 60−90, ≥90 mL/min/1.73 m^2, and unknown); smoking status (non-, ex-, current smoker, and unknown); alcoholic drinks (<1, 1–2, 3–4, ≥5 days per week, and unknown); aerobic physical activity (sufficient, insufficient, and unknown); previous history of cardiovascular disease, chronic kidney disease, and chronic obstructive pulmonary disease; history of medication use for diabetes mellitus, dyslipidemia, and hypertension; and missing indicators (BMI missing indicator [yes or no], blood pressure missing indicator [yes or no], fasting blood glucose missing indicator [yes or no], serum total cholesterol missing indicator [yes or no], glomerular filtration rate missing indicator [yes or no], smoking status missing indicator [yes or no], alcoholic drinks missing indicator [yes or no], and aerobic physical activity missing indicator [yes or no]).

‡ Comparators defined only 1:5 matched comparators in each patient group at the same index date to reduce immortal bias.

Table S9. The propensity-score-matched subgroup analysis of HR (95% CI) of **atopic dermatitis** following COVID-19 diagnosis stratified by COVID-19 severity, SARS-CoV-2 strain type, and number of vaccinations in the main cohort (South Korea; n=836,164).

Factors	Group	Exposure	Events/total number (%)	HR (95% CI)	
				Model 1 [a]	Model 2 [b]
COVID-19 severity	Total	Non-infected control	610/688,340 (0.09)	1.00 (reference)	1.00 (reference)
		Mild COVID-19	125/127,641 (0.10)	1.14 (0.94 to 1.39)	1.16 (0.96 to 1.40)
		Moderate to severe COVID-19	23/20,183 (0.11)	1.18 (0.78 to 1.80)	1.08 (0.71 to 1.65)
Strain type (original)	Total	Non-infected control at the same index date‡	478/220,904 (0.22)	1.00 (reference)	1.00 (reference)
		Original SARS-CoV-2 infection	122/46,900 (0.26)	**1.22 (1.00 to 1.48)**	1.21 (0.99 to 1.48)
Strain type (delta)	Total	Non-infected control at the same index date‡	132/467,436 (0.03)	1.00 (reference)	1.00 (reference)
		Delta SARS-CoV-2 infection	26/100,924 (0.03)	0.92 (0.60 to 1.40)	0.92 (0.60 to 1.40)
Number of SARS-CoV-2 vaccinations	Patients with COVID-19	Non-infected control	610/688,340 (0.09)	1.00 (reference)	1.00 (reference)
		Without vaccination	124/68,456 (0.18)	**1.22 (1.00 to 1.48)**	**1.21 (1.00 to 1.47)**
		Vaccination 1 time	13/14,125 (0.09)	1.26 (0.73 to 2.18)	1.28 (0.74 to 2.21)
		Vaccination ≥2 times	11/65,243 (0.02)	0.67 (0.36 to 1.22)	0.66 (0.36 to 1.20)

BMI, body mass index; CI, confidence interval; HR, hazard ratio; SARS-CoV-2, severe acute respiratory syndrome coronavirus.

The data in bold indicate significant differences ($P < 0.05$).

[a] **Model 1**: Adjusted for age (20–39, 40–59, and ≥60 years) and sex.

[b] **Model 2**: Adjusted for age (20–39, 40–59, and ≥60 years); sex; household income (low income, middle income, and high income); region of residence (urban and rural); Charlson comorbidity index (0, 1, and ≥2); BMI (underweight [<18.5 kg/m^2], normal [18.5–23.0 kg/m^2], overweight [23.0–25.0 kg/m^2], obese [≥25.0 kg/m^2], and unknown); blood pressure (systolic blood pressure <140 mmHg and diastolic blood pressure <90 mmHg, systolic blood pressure ≥140 mmHg or diastolic blood pressure ≥90 mmHg, and unknown); fasting blood glucose (<100, ≥100 mg/dL, and unknown); serum total cholesterol (<200, 200–240, ≥240

mg/dL, and unknown); glomerular filtration rate (<60, 60−90, ≥90 mL/min/1.73 m^2, and unknown); smoking status (non-, ex-, current smoker, and unknown); alcoholic drinks (<1, 1–2, 3–4, ≥5 days per week, and unknown); aerobic physical activity (sufficient, insufficient, and unknown); previous history of cardiovascular disease, chronic kidney disease, and chronic obstructive pulmonary disease; history of medication use for diabetes mellitus, dyslipidemia, and hypertension; and missing indicators (BMI missing indicator [yes or no], blood pressure missing indicator [yes or no], fasting blood glucose missing indicator [yes or no], serum total cholesterol missing indicator [yes or no], glomerular filtration rate missing indicator [yes or no], smoking status missing indicator [yes or no], alcoholic drinks missing indicator [yes or no], and aerobic physical activity missing indicator [yes or no]).

‡ Comparators defined only 1:5 matched comparators in each patient group at the same index date to reduce immortal bias.

Table S10. The propensity-score-matched subgroup analysis of HR (95% CI) of **food allergy** following COVID-19 diagnosis stratified by COVID-19 severity, SARS-CoV-2 strain type, and number of vaccinations in the main cohort (South Korea; n=836,164).

Factors	Group	Exposure	Events/total number (%)	HR (95% CI)	
				Model 1 [a]	Model 2 [b]
COVID-19 severity	Total	Non-infected control	853/688,340 (0.12)	1.00 (reference)	1.00 (reference)
		Mild COVID-19	111/127,641 (0.09)	**0.75 (0.62 to 0.92)**	**0.76 (0.62 to 0.93)**
		Moderate to severe COVID-19	41/20,183 (0.20)	1.28 (0.93 to 1.75)	1.21 (0.88 to 1.67)
Strain type (original)	Total	Non-infected control at the same index date‡	649/220,904 (0.29)	1.00 (reference)	1.00 (reference)
		Original SARS-CoV-2 infection	120/46,900 (0.26)	0.88 (0.73 to 1.07)	0.88 (0.73 to 1.07)
Strain type (delta)	Total	Non-infected control at the same index date‡	204/467,436 (0.04)	1.00 (reference)	1.00 (reference)
		Delta SARS-CoV-2 infection	32/100,924 (0.03)	0.73 (0.51 to 1.06)	0.73 (0.50 to 1.06)
Number of SARS-CoV-2 vaccinations	Patients with COVID-19	Non-infected control	853/688,340 (0.12)	1.00 (reference)	1.00 (reference)
		Without vaccination	125/68,456 (0.18)	0.91 (0.75 to 1.10)	0.91 (0.75 to 1.10)
		Vaccination 1 time	17/14,125 (0.12)	1.06 (0.65 to 1.71)	1.06 (0.65 to 1.71)
		Vaccination ≥2 times	10/65,243 (0.02)	**0.38 (0.20 to 0.71)**	**0.38 (0.20 to 0.72)**

BMI, body mass index; CI, confidence interval; HR, hazard ratio; SARS-CoV-2, severe acute respiratory syndrome coronavirus.

The data in bold indicate significant differences ($P < 0.05$).

[a] **Model 1**: Adjusted for age (20–39, 40–59, and ≥60 years) and sex.

[b] **Model 2**: Adjusted for age (20–39, 40–59, and ≥60 years), sex, household income (low income, middle income, and high income), region of residence (urban and rural), Charlson comorbidity index (0, 1, and ≥2), obesity (underweight [<18.5 kg/m^2], normal [18.5–23.0 kg/m^2], overweight [23.0–25.0 kg/m^2], obese [≥25.0 kg/m^2], and unknown), blood pressure (systolic blood pressure <140 mmHg and diastolic blood pressure <90 mmHg, systolic blood pressure ≥140 mmHg or diastolic blood pressure ≥90 mmHg, and unknown), fasting blood glucose (<100, ≥100 mg/dL, and unknown), serum total cholesterol (<200, 200–240, ≥240

mg/dL, and unknown), glomerular filtration rate (<60, 60–90, ≥90 mL/min/1.73 m^2, and unknown), smoking status (non-, ex-, current smoker, and unknown), alcoholic drinks (<1, 1–2, 3–4, ≥5 days per week, and unknown), aerobic physical activity (sufficient, insufficient, and unknown), previous history of cardiovascular disease, chronic kidney disease, and chronic obstructive pulmonary disease, history of medication use for diabetes mellitus, dyslipidemia, and hypertension, and missing indicators (obesity missing indicator [yes or no], blood pressure missing indicator [yes or no], fasting blood glucose missing indicator [yes or no], serum total cholesterol missing indicator [yes or no], glomerular filtration rate missing indicator [yes or no], smoking status missing indicator [yes or no], alcoholic drinks missing indicator [yes or no], and aerobic physical activity missing indicator [yes or no]).

‡ Comparators defined only 1:5 matched comparators in each patient group at the same index date to reduce immortal bias.

Table S11. Stratification analysis for the long-term sequelae risk of **incident allergic diseases** following COVID-19 diagnosis in the propensity score-matched main cohort (South Korea; n=836,164).

Parameter	Exposure	n (%)	Incidence rate*	HR (95% CI)	
				Model 1 [a]	Model 2 [b]
**Total**					
	None	688,340 (82.32)	24.0	1.00 (reference)	1.00 (reference)
	Patients with COVID-19	147,824 (17.68)	28.7	**1.20 (1.13 to 1.27)**	**1.20 (1.13 to 1.27)**
**Male**					
	None	306,165 (82.10)	28.1	1.00 (reference)	1.00 (reference)
	Patients with COVID-19	66,749 (17.90)	33.1	**1.18 (1.09 to 1.28)**	**1.18 (1.09 to 1.28)**
**Female**					
	None	299,203 (82.51)	21.0	1.00 (reference)	1.00 (reference)
	Patients with COVID-19	63,421 (17.49)	25.7	**1.22 (1.12 to 1.34)**	**1.22 (1.12 to 1.34)**
**Age 20–39 years**					
	None	173,290 (82.39)	23.5	1.00 (reference)	1.00 (reference)
	Patients with COVID-19	37,030 (17.61)	26.1	1.11 (0.99 to 1.25)	1.11 (0.99 to 1.25)
**Age 40–59 years**					
	None	299,203 (82.51)	21.0	1.00 (reference)	1.00 (reference)
	Patients with COVID-19	63,421 (17.49)	25.7	**1.22 (1.12 to 1.34)**	**1.22 (1.12 to 1.34)**
**Age ≥60 years**					
	None	215,847 (82.00)	29.3	1.00 (reference)	1.00 (reference)
	Patients with COVID-19	47,373 (18.00)	36.1	**1.23 (1.11 to 1.36)**	**1.23 (1.11 to 1.36)**
**Low income**					
	None	298,798 (82.41)	24.6	1.00 (reference)	1.00 (reference)
	Patients with COVID-19	63,769 (17.59)	28.9	**1.17 (1.07 to 1.28)**	**1.17 (1.07 to 1.28)**

**Middle income**					
	None	262,108 (82.38)	23.7	1.00 (reference)	1.00 (reference)
	Patients with COVID-19	56,080 (17.62)	29.7	**1.26 (1.14 to 1.38)**	**1.25 (1.14 to 1.38)**
**High income**					
	None	127,434 (82.00)	23.2	1.00 (reference)	1.00 (reference)
	Patients with COVID-19	27,975 (18.00)	26.3	1.13 (0.98 to 1.30)	1.13 (0.98 to 1.30)
**CCI, 0 score**					
	None	597,115 (82.47)	22.8	1.00 (reference)	1.00 (reference)
	Patients with COVID-19	126,952 (17.53)	28.0	**1.23 (1.15 to 1.31)**	**1.23 (1.15 to 1.31)**
**CCI, 1 scores**					
	None	63,117 (82.15)	31.6	1.00 (reference)	1.00 (reference)
	Patients with COVID-19	13,712 (17.85)	31.6	1.00 (0.85 to 1.19)	1.01 (0.86 to 1.20)
**CCI, ≥2 scores**					
	None	28,108 (79.70)	30.1	1.00 (reference)	1.00 (reference)
	Patients with COVID-19	7,160 (20.30)	33.9	1.13 (0.88 to 1.45)	1.13 (0.88 to 1.45)
**BMI, <18.5 kg/m^2**					
	None	17,997 (81.42)	23.5	1.00 (reference)	1.00 (reference)
	Patients with COVID-19	4,108 (18.58)	31.5	1.34 (0.95 to 1.89)	1.34 (0.95 to 1.89)
**BMI, 18.5-23.0 kg/m^2**					
	None	220,919 (82.24)	24.3	1.00 (reference)	1.00 (reference)
	Patients with COVID-19	47,707 (17.76)	27.9	**1.15 (1.03 to 1.27)**	**1.15 (1.03 to 1.27)**
**BMI, 23.0-25.0 kg/m^2**					
	None	159,861 (82.35)	24.0	1.00 (reference)	1.00 (reference)
	Patients with COVID-19	34,255 (17.65)	29.3	**1.22 (1.08 to 1.37)**	**1.22 (1.08 to 1.38)**

**BMI, ≥25.0 kg/m^2**					
	None	289,452 (82.43)	23.8	1.00 (reference)	1.00 (reference)
	Patients with COVID-19	61,718 (17.57)	28.7	**1.21 (1.11 to 1.33)**	**1.21 (1.11 to 1.33)**
**Drinker**					
	None	293,797 (82.42)	22.2	1.00 (reference)	1.00 (reference)
	Patients with COVID-19	62,665 (17.58)	25.6	**1.16 (1.05 to 1.27)**	**1.15 (1.05 to 1.27)**
**Non-drinker**					
	None	394,032 (82.24)	25.3	1.00 (reference)	1.00 (reference)
	Patients with COVID-19	85,120 (17.76)	31.0	**1.22 (1.14 to 1.32)**	**1.22 (1.14 to 1.32)**
**Sufficient physical activity**					
	None	340,507 (82.39)	23.6	1.00 (reference)	1.00 (reference)
	Patients with COVID-19	72,760 (17.61)	27.8	**1.18 (1.08 to 1.28)**	**1.18 (1.08 to 1.28)**
**Insufficient physical activity**					
	None	347,204 (82.24)	24.4	1.00 (reference)	1.00 (reference)
	Patients with COVID-19	74,978 (17.76)	29.6	**1.21 (1.12 to 1.31)**	**1.21 (1.12 to 1.31)**
**Smoker (current and ex-)**					
	None	251,296 (82.46)	21.3	1.00 (reference)	1.00 (reference)
	Patients with COVID-19	53,445 (17.54)	23.3	1.10 (0.99 to 1.22)	1.10 (0.99 to 1.22)
**Non-smoker**					
	None	436,584 (82.23)	25.5	1.00 (reference)	1.00 (reference)
	Patients with COVID-19	94,341 (17.77)	31.7	**1.24 (1.16 to 1.33)**	**1.24 (1.16 to 1.33)**
**Rural residence**					
	None	368,924 (82.35)	25.3	1.00 (reference)	1.00 (reference)
	Patients with COVID-19	79,097 (17.65)	29.9	**1.18 (1.09 to 1.28)**	**1.18 (1.09 to 1.28)**

**Urban residence**					
	None	319,416 (82.29)	22.6	1.00 (reference)	1.00 (reference)
	Patients with COVID-19	68,727 (17.71)	27.4	**1.21 (1.11 to 1.32)**	**1.21 (1.11 to 1.32)**
**Strain type (original)**‡					
	None	220,904 (82.49)	25.3	1.00 (reference)	1.00 (reference)
	Patients with COVID-19	46,900 (17.51)	30.4	**1.20 (1.12 to 1.29)**	**1.20 (1.12 to 1.29)**
**Strain type (delta)**‡					
	None	467,436 (82.24)	20.9	1.00 (reference)	1.00 (reference)
	Patients with COVID-19	100,924 (17.76)	24.7	**1.18 (1.05 to 1.32)**	**1.18 (1.05 to 1.32)**

BMI, body mass index; CCI, Charlson comorbidity index; CI, confidence interval; HR, hazard ratio.

* Incidence rate is expressed as per 1,000 person-years.

‡ We considered the original SARS-CoV-2 if the initial infection was before July 31, 2021, and the delta variant was from August 1, 2021.

The data in bold indicate significant differences (*P* < 0.05).

[a] **Model 1**: Adjusted for age (20–39, 40–59, and ≥60 years) and sex.

[b] **Model 2**: Adjusted for age (20–39, 40–59, and ≥60 years); sex; household income (low income, middle income, and high income); region of residence (urban and rural); Charlson comorbidity index (0, 1, and ≥2); BMI (underweight [<18.5 kg/m^2], normal [18.5–23.0 kg/m^2], overweight [23.0–25.0 kg/m^2], obese [≥25.0 kg/m^2], and unknown); blood pressure (systolic blood pressure <140 mmHg and diastolic blood pressure <90 mmHg, systolic blood pressure ≥140 mmHg or diastolic blood pressure ≥90 mmHg, and unknown); fasting blood glucose (<100, ≥100 mg/dL, and unknown); serum total cholesterol (<200, 200–240, ≥240 mg/dL, and unknown); glomerular filtration rate (<60, 60−90, ≥90 mL/min/1.73 m^2, and unknown); smoking status (non-, ex-, current smoker, and unknown); alcoholic drinks (<1, 1–2, 3–4, ≥5 days per week, and unknown); aerobic physical activity (sufficient, insufficient, and unknown); previous history of cardiovascular disease, chronic kidney disease, and chronic obstructive pulmonary disease; history of medication use for diabetes mellitus, dyslipidemia, and hypertension; and missing indicators (BMI missing indicator [yes or no], blood pressure missing indicator [yes or no], fasting blood glucose missing indicator

[yes or no], serum total cholesterol missing indicator [yes or no], glomerular filtration rate missing indicator [yes or no], smoking status missing indicator [yes or no], alcoholic drinks missing indicator [yes or no], and aerobic physical activity missing indicator [yes or no]).

Table S12. Stratification analysis for the long-term sequelae risk of **incident asthma** following COVID-19 diagnosis in the propensity score-matched main cohort (South Korea; n=836,164).

Parameter	Exposure	n (%)	Incidence rate*	HR (95% CI)	
				Model 1 [a]	Model 2 [b]
**Total**					
	None	688,340 (82.32)	1.0	1.00 (reference)	1.00 (reference)
	Patients with COVID-19	147,824 (17.68)	2.2	**2.27 (1.81 to 2.85)**	**2.25 (1.80 to 2.83)**
**Male**					
	None	382,175 (82.50)	0.8	1.00 (reference)	1.00 (reference)
	Patients with COVID-19	81,075 (17.50)	1.9	**2.25 (1.62 to 3.14)**	**2.25 (1.61 to 3.14)**
**Female**					
	None	306,165 (82.10)	1.1	1.0 (reference)	1.0 (reference)
	Patients with COVID-19	66,749 (17.90)	2.6	**2.29 (1.67 to 3.12)**	**2.26 (1.66 to 3.09)**
**Age 20–39 years**					
	None	173,290 (82.39)	0.8	1.00 (reference)	1.00 (reference)
	Patients with COVID-19	37,030 (17.61)	2.3	**2.88 (1.83 to 4.54)**	**2.87 (1.82 to 4.53)**
**Age 40–59 years**					
	None	299,203 (82.51)	0.7	1.00 (reference)	1.00 (reference)
	Patients with COVID-19	63,421 (17.49)	1.8	**2.60 (1.79 to 3.79)**	**2.61 (1.79 to 3.80)**
**Age ≥60 years**					
	None	215,847 (82.00)	1.6	1.00 (reference)	1.00 (reference)

	Patients with COVID-19	47,373 (18.00)	2.7	**1.75 (1.20 to 2.53)**	**1.70 (1.17 to 2.46)**
**Low income**					
	None	298,798 (82.41)	1.0	1.00 (reference)	1.00 (reference)
	Patients with COVID-19	63,769 (17.59)	2.0	**2.02 (1.42 to 2.88)**	**2.03 (1.43 to 2.89)**
**Middle income**					
	None	262,108 (82.38)	1.0	1.00 (reference)	1.00 (reference)
	Patients with COVID-19	56,080 (17.62)	2.2	**2.32 (1.61 to 3.36)**	**2.27 (1.57 to 3.29)**
**High income**					
	None	127,434 (82.00)	0.9	1.00 (reference)	1.00 (reference)
	Patients with COVID-19	27,975 (18.00)	2.5	**2.77 (1.67 to 4.60)**	**2.67 (1.61 to 4.43)**
**CCI, 0 score**					
	None	597,115 (82.47)	0.7	1.00 (reference)	1.00 (reference)
	Patients with COVID-19	126,952 (17.53)	2.1	**2.82 (2.17 to 3.67)**	**2.82 (2.16 to 3.66)**
**CCI, 1 scores**					
	None	63,117 (82.15)	2.2	1.00 (reference)	1.00 (reference)
	Patients with COVID-19	13,712 (17.85)	2.8	1.29 (0.73 to 2.28)	1.33 (0.75 to 2.36)
**CCI, ≥2 scores**					
	None	28,108 (79.70)	2.7	1.00 (reference)	1.00 (reference)
	Patients with COVID-19	7,160 (20.30)	3.0	1.13 (0.49 to 2.60)	1.03 (0.45 to 2.39)
**BMI, <18.5 kg/m^2**					

	None	17,997 (81.42)	1.0	1.00 (reference)	1.00 (reference)
	Patients with COVID-19	4,108 (18.58)	1.5	1.46 (0.29 to 7.23)	1.57 (0.31 to 8.04)
**BMI, 18.5-23.0 kg/m^2**					
	None	220,919 (82.24)	0.9	1.00 (reference)	1.00 (reference)
	Patients with COVID-19	47,707 (17.76)	1.5	**1.62 (1.02 to 2.58)**	**1.60 (1.01 to 2.54)**
**BMI, 23.0-25.0 kg/m^2**					
	None	159,861 (82.35)	1.1	1.00 (reference)	1.00 (reference)
	Patients with COVID-19	34,255 (17.65)	2.2	**2.06 (1.30 to 3.26)**	**2.03 (1.28 to 3.22)**
**BMI, ≥25.0 kg/m^2**					
	None	289,452 (82.43)	0.9	1.00 (reference)	1.00 (reference)
	Patients with COVID-19	61,718 (17.57)	2.8	**2.95 (2.13 to 4.09)**	**2.94 (2.12 to 4.08)**
**Drinker**					
	None	293,797 (82.42)	0.8	1.00 (reference)	1.00 (reference)
	Patients with COVID-19	62,665 (17.58)	2.3	**3.03 (2.12 to 4.32)**	**3.03 (2.13 to 4.33)**
**Non-drinker**					
	None	394,032 (82.24)	1.1	1.00 (reference)	1.00 (reference)
	Patients with COVID-19	85,120 (17.76)	2.1	**1.88 (1.39 to 2.53)**	**1.87 (1.38 to 2.52)**
**Sufficient physical activity**					
	None	340,507 (82.39)	0.8	1.00 (reference)	1.00 (reference)

	Patients with COVID-19	72,760 (17.61)	1.8	**2.19 (1.54 to 3.12)**	**2.21 (1.55 to 3.14)**
**Insufficient physical activity**					
	None	347,204 (82.24)	1.1	1.00 (reference)	1.00 (reference)
	Patients with COVID-19	74,978 (17.76)	2.6	**2.34 (1.74 to 3.16)**	**2.31 (1.71 to 3.11)**
**Smoker (current and ex-)**					
	None	251,296 (82.46)	0.9	1.00 (reference)	1.00 (reference)
	Patients with COVID-19	53,445 (17.54)	1.6	**1.78 (1.15 to 2.74)**	**1.78 (1.15 to 2.75)**
**Non-smoker**					
	None	436,584 (82.23)	1.0	1.00 (reference)	1.00 (reference)
	Patients with COVID-19	94,341 (17.77)	2.6	**2.50 (1.92 to 3.27)**	**2.48 (1.90 to 3.24)**
**Rural residence**					
	None	368,924 (82.35)	1.0	1.00 (reference)	1.00 (reference)
	Patients with COVID-19	79,097 (17.65)	2.1	**2.04 (1.49 to 2.80)**	**2.03 (1.48 to 2.79)**
**Urban residence**					
	None	319,416 (82.29)	0.9	1.00 (reference)	1.00 (reference)
	Patients with COVID-19	68,727 (17.71)	2.3	**2.56 (1.85 to 3.56)**	**2.53 (1.82 to 3.51)**
**Strain type (original)**[‡]					
	None	220,904 (82.49)	1.1	1.00 (reference)	1.00 (reference)

	Patients with COVID-19	46,900 (17.51)	2.3	**2.08 (1.59 to 2.70)**	**2.07 (1.59 to 2.69)**
**Strain type (delta)**[‡]					
	None	467,436 (82.24)	0.7	1.00 (reference)	1.00 (reference)
	Patients with COVID-19	100,924 (17.76)	2.1	**3.01 (1.91 to 4.72)**	**2.97 (1.89 to 4.66)**

BMI, body mass index; CCI, Charlson comorbidity index; CI, confidence interval; HR, hazard ratio.

* Incidence rate is expressed as per 1,000 person-years.

[‡] We considered the original SARS-CoV-2 if the initial infection was before July 31, 2021, and the delta variant was from August 1, 2021.

The data in bold indicate significant differences ($P < 0.05$).

[a] **Model 1**: Adjusted for age (20–39, 40–59, and ≥60 years) and sex.

[b] **Model 2**: Adjusted for age (20–39, 40–59, and ≥60 years); sex; household income (low income, middle income, and high income); region of residence (urban and rural); Charlson comorbidity index (0, 1, and ≥2); BMI (underweight [<18.5 kg/m^2], normal [18.5–23.0 kg/m^2], overweight [23.0–25.0 kg/m^2], obese [≥25.0 kg/m^2], and unknown); blood pressure (systolic blood pressure <140 mmHg and diastolic blood pressure <90 mmHg, systolic blood pressure ≥140 mmHg or diastolic blood pressure ≥90 mmHg, and unknown); fasting blood glucose (<100, ≥100 mg/dL, and unknown); serum total cholesterol (<200, 200–240, ≥240 mg/dL, and unknown); glomerular filtration rate (<60, 60−90, ≥90 mL/min/1.73 m^2, and unknown); smoking status (non-, ex-, current smoker, and unknown); alcoholic drinks (<1, 1–2, 3–4, ≥5 days per week, and unknown); aerobic physical activity (sufficient, insufficient, and unknown); previous history of cardiovascular disease, chronic kidney disease, and chronic obstructive pulmonary disease; history of medication use for diabetes mellitus, dyslipidemia, and hypertension; and missing indicators (BMI missing indicator [yes or no], blood pressure missing indicator [yes or no], fasting blood glucose missing indicator [yes or no], serum total cholesterol missing indicator [yes or no], glomerular filtration rate missing indicator [yes or no], smoking status missing indicator [yes or no], alcoholic drinks missing indicator [yes or no], and aerobic physical activity missing indicator [yes or no]).

Table S13. Stratification analysis for the long-term sequelae risk of **incident allergic rhinitis** following COVID-19 diagnosis in the propensity score-matched main cohort (South Korea; n=836,164).

Parameter	Exposure	n (%)	Incidence rate*	HR (95% CI)	
				Model 1 [a]	Model 2 [b]
**Total**					
	None	688,340 (82.32)	17.3	1.00 (reference)	1.00 (reference)
	Patients with COVID-19	147,824 (17.68)	21.3	**1.23 (1.15 to 1.32)**	**1.23 (1.15 to 1.32)**
**Male**					
	None	382,175 (82.50)	14.9	1.00 (reference)	1.00 (reference)
	Patients with COVID-19	81,075 (17.50)	18.9	**1.27 (1.16 to 1.41)**	**1.27 (1.16 to 1.40)**
**Female**					
	None	173,290 (82.39)	17.9	1.00 (reference)	1.00 (reference)
	Patients with COVID-19	37,030 (17.61)	20.2	1.13 (0.98 to 1.29)	1.13 (0.98 to 1.29)
**Age 20–39 years**					
	None	299,203 (82.51)	15.0	1.00 (reference)	1.00 (reference)
	Patients with COVID-19	63,421 (17.49)	19.5	**1.30 (1.17 to 1.44)**	**1.30 (1.17 to 1.45)**
**Age 40–59 years**					
	None	299,203 (82.51)	15.0	1.00 (reference)	1.00 (reference)
	Patients with COVID-19	63,421 (17.49)	19.5	**1.30 (1.17 to 1.44)**	**1.30 (1.17 to 1.45)**
**Age ≥60 years**					
	None	215,847 (82.00)	20.5	1.00 (reference)	1.00 (reference)
	Patients with COVID-19	47,373 (18.00)	25.2	**1.23 (1.09 to 1.38)**	**1.23 (1.10 to 1.39)**
**Low income**					
	None	298,798 (82.41)	17.6	1.00 (reference)	1.00 (reference)

	Patients with COVID-19	63,769 (17.59)	21.3	**1.21 (1.09 to 1.34)**	**1.21 (1.09 to 1.34)**
**Middle income**					
	None	262,108 (82.38)	17.2	1.00 (reference)	1.00 (reference)
	Patients with COVID-19	56,080 (17.62)	23.0	**1.34 (1.20 to 1.49)**	**1.34 (1.20 to 1.49)**
**High income**					
	None	127,434 (82.00)	16.5	1.00 (reference)	1.00 (reference)
	Patients with COVID-19	27,975 (18.00)	17.6	1.06 (0.90 to 1.26)	1.07 (0.90 to 1.26)
**CCI, 0 score**					
	None	597,115 (82.47)	16.7	1.00 (reference)	1.00 (reference)
	Patients with COVID-19	126,952 (17.53)	21.1	**1.26 (1.17 to 1.36)**	**1.26 (1.17 to 1.36)**
**CCI, 1 scores**					
	None	63,117 (82.15)	21.0	1.00 (reference)	1.00 (reference)
	Patients with COVID-19	13,712 (17.85)	21.8	1.04 (0.85 to 1.28)	1.06 (0.86 to 1.29)
**CCI, ≥2 scores**					
	None	28,108 (79.70)	19.9	1.00 (reference)	1.00 (reference)
	Patients with COVID-19	7,160 (20.30)	23.0	1.15 (0.85 to 1.56)	1.17 (0.86 to 1.59)
**BMI, <18.5 kg/m^2**					
	None	17,997 (81.42)	17.4	1.00 (reference)	1.00 (reference)
	Patients with COVID-19	4,108 (18.58)	21.7	1.24 (0.83 to 1.88)	1.24 (0.82 to 1.87)
**BMI, 18.5-23.0 kg/m^2**					
	None	220,919 (82.24)	17.8	1.00 (reference)	1.00 (reference)
	Patients with COVID-19	47,707 (17.76)	21.1	**1.18 (1.05 to 1.33)**	**1.18 (1.04 to 1.33)**
**BMI, 23.0-25.0 kg/m^2**					
	None	159,861 (82.35)	17.1	1.00 (reference)	1.00 (reference)

	Patients with COVID-19	34,255 (17.65)	22.0	**1.29 (1.12 to 1.48)**	**1.29 (1.12 to 1.48)**
**BMI, ≥25.0 kg/m²**					
	None	289,452 (82.43)	16.9	1.00 (reference)	1.00 (reference)
	Patients with COVID-19	61,718 (17.57)	21.0	**1.24 (1.12 to 1.38)**	**1.24 (1.12 to 1.38)**
**Drinker**					
	None	293,797 (82.42)	16.1	1.00 (reference)	1.00 (reference)
	Patients with COVID-19	62,665 (17.58)	19.2	**1.19 (1.07 to 1.33)**	**1.19 (1.07 to 1.33)**
**Non-drinker**					
	None	394,032 (82.24)	18.1	1.00 (reference)	1.00 (reference)
	Patients with COVID-19	85,120 (17.76)	22.8	**1.26 (1.15 to 1.37)**	**1.26 (1.15 to 1.37)**
**Sufficient physical activity**					
	None	340,507 (82.39)	16.9	1.00 (reference)	1.00 (reference)
	Patients with COVID-19	72,760 (17.61)	20.8	**1.23 (1.12 to 1.36)**	**1.23 (1.12 to 1.36)**
**Insufficient physical activity**					
	None	347,204 (82.24)	17.6	1.00 (reference)	1.00 (reference)
	Patients with COVID-19	74,978 (17.76)	21.7	**1.23 (1.12 to 1.35)**	**1.23 (1.12 to 1.35)**
**Smoker (current and ex-)**					
	None	251,296 (82.46)	15.5	1.00 (reference)	1.00 (reference)
	Patients with COVID-19	53,445 (17.54)	17.7	**1.14 (1.01 to 1.29)**	**1.14 (1.01 to 1.29)**
**Non-smoker**					
	None	436,584 (82.23)	18.3	1.00 (reference)	1.00 (reference)
	Patients with COVID-19	94,341 (17.77)	23.3	**1.27 (1.17 to 1.38)**	**1.27 (1.17 to 1.38)**

**Rural residence**					
	None	368,924 (82.35)	18.2	1.00 (reference)	1.00 (reference)
	Patients with COVID-19	79,097 (17.65)	21.8	**1.20 (1.09 to 1.32)**	**1.20 (1.09 to 1.32)**
**Urban residence**					
	None	319,416 (82.29)	16.3	1.00 (reference)	1.00 (reference)
	Patients with COVID-19	68,727 (17.71)	20.7	**1.27 (1.15 to 1.40)**	**1.27 (1.15 to 1.40)**
**Strain type (original)**[‡]					
	None	220,904 (82.49)	17.9	1.00 (reference)	1.00 (reference)
	Patients with COVID-19	46,900 (17.51)	22.2	**1.24 (1.14 to 1.34)**	**1.24 (1.14 to 1.34)**
**Strain type (delta)**[‡]					
	None	467,436 (82.24)	15.7	1.00 (reference)	1.00 (reference)
	Patients with COVID-19	100,924 (17.76)	19.2	**1.22 (1.07 to 1.39)**	**1.22 (1.07 to 1.39)**

BMI, body mass index; CCI, Charlson comorbidity index; CI, confidence interval; HR, hazard ratio.

* Incidence rate is expressed as per 1,000 person-years.

‡ We considered the original SARS-CoV-2 if the initial infection was before July 31, 2021, and the delta variant was from August 1, 2021.

The data in bold indicate significant differences ($P < 0.05$).

[a] **Model 1**: Adjusted for age (20–39, 40–59, and ≥60 years) and sex.

[b] **Model 2**: Adjusted for age (20–39, 40–59, and ≥60 years); sex; household income (low income, middle income, and high income); region of residence (urban and rural); Charlson comorbidity index (0, 1, and ≥2); BMI (underweight [<18.5 kg/m^2], normal [18.5–23.0 kg/m^2], overweight [23.0–25.0 kg/m^2], obese [≥25.0 kg/m^2], and unknown); blood pressure (systolic blood pressure <140 mmHg and diastolic blood pressure <90 mmHg, systolic blood pressure ≥140 mmHg or diastolic blood pressure ≥90 mmHg, and unknown); fasting blood glucose (<100, ≥100 mg/dL, and unknown); serum total cholesterol (<200, 200–240, ≥240 mg/dL, and unknown); glomerular filtration rate (<60, 60−90, ≥90 mL/min/1.73 m^2, and unknown); smoking status (non-, ex-, current smoker, and unknown);

alcoholic drinks (<1, 1–2, 3–4, ≥5 days per week, and unknown); aerobic physical activity (sufficient, insufficient, and unknown); previous history of cardiovascular disease, chronic kidney disease, and chronic obstructive pulmonary disease; history of medication use for diabetes mellitus, dyslipidemia, and hypertension; and missing indicators (BMI missing indicator [yes or no], blood pressure missing indicator [yes or no], fasting blood glucose missing indicator [yes or no], serum total cholesterol missing indicator [yes or no], glomerular filtration rate missing indicator [yes or no], smoking status missing indicator [yes or no], alcoholic drinks missing indicator [yes or no], and aerobic physical activity missing indicator [yes or no]).

Table S14. Stratification analysis for the long-term sequelae risk of **incident atopic dermatitis** following COVID-19 diagnosis in the propensity score-matched main cohort (South Korea; n=836,164).

Parameter	Exposure	n (%)	Incidence rate*	HR (95% CI)	
				Model 1 [a]	Model 2 [b]
**Total**					
	None	688,340 (82.32)	2.6	1.00 (reference)	1.00 (reference)
	Patients with COVID-19	147,824 (17.68)	3.0	1.15 (0.96 to 1.37)	1.15 (0.96 to 1.37)
**Male**					
	None	382,175 (82.50)	2.2	1.00 (reference)	1.00 (reference)
	Patients with COVID-19	81,075 (17.50)	2.2	1.02 (0.77 to 1.34)	1.01 (0.77 to 1.33)
**Female**					
	None	306,165 (82.10)	3.0	1.00 (reference)	1.00 (reference)
	Patients with COVID-19	66,749 (17.90)	3.8	**1.27 (1.00 to 1.61)**	**1.27 (1.01 to 1.61)**
**Age 20–39 years**					
	None	173,290 (82.39)	3.2	1.00 (reference)	1.00 (reference)
	Patients with COVID-19	37,030 (17.61)	3.0	0.95 (0.67 to 1.33)	0.95 (0.67 to 1.34)
**Age 40–59 years**					
	None	299,203 (82.51)	2.1	1.00 (reference)	1.00 (reference)
	Patients with COVID-19	63,421 (17.49)	2.4	1.13 (0.85 to 1.52)	1.13 (0.85 to 1.52)
**Age ≥60 years**					
	None	215,847 (82.00)	2.7	1.00 (reference)	1.00 (reference)
	Patients with COVID-19	47,373 (18.00)	3.8	**1.39 (1.03 to 1.89)**	**1.38 (1.02 to 1.88)**
**Low income**					
	None	298,798 (82.41)	2.7	1.00 (reference)	1.00 (reference)

	Patients with COVID-19	63,769 (17.59)	2.7	1.01 (0.76 to 1.34)	1.01 (0.76 to 1.34)
**Middle income**					
	None	262,108 (82.38)	2.3	1.00 (reference)	1.00 (reference)
	Patients with COVID-19	56,080 (17.62)	2.4	1.06 (0.77 to 1.46)	1.06 (0.77 to 1.46)
**High income**					
	None	127,434 (82.00)	2.9	1.00 (reference)	1.00 (reference)
	Patients with COVID-19	27,975 (18.00)	4.6	**1.57 (1.12 to 2.21)**	**1.57 (1.11 to 2.21)**
**CCI, 0 score**					
	None	597,115 (82.47)	2.5	1.00 (reference)	1.00 (reference)
	Patients with COVID-19	126,952 (17.53)	2.9	1.16 (0.95 to 1.41)	1.16 (0.95 to 1.41)
**CCI, 1 scores**					
	None	63,117 (82.15)	3.1	1.00 (reference)	1.00 (reference)
	Patients with COVID-19	13,712 (17.85)	3.6	1.17 (0.71 to 1.93)	1.17 (0.71 to 1.94)
**CCI, ≥2 scores**					
	None	28,108 (79.70)	3.5	1.00 (reference)	1.00 (reference)
	Patients with COVID-19	7,160 (20.30)	3.4	0.98 (0.45 to 2.11)	0.96 (0.44 to 2.08)
**BMI, <18.5 kg/m^2**					
	None	17,997 (81.42)	2.3	1.00 (reference)	1.00 (reference)
	Patients with COVID-19	4,108 (18.58)	3.0	1.32 (0.44 to 4.02)	1.34 (0.44 to 4.09)
**BMI, 18.5-23.0 kg/m^2**					
	None	220,919 (82.24)	2.8	1.00 (reference)	1.00 (reference)
	Patients with COVID-19	47,707 (17.76)	3.3	1.18 (0.87 to 1.60)	1.18 (0.87 to 1.59)
**BMI, 23.0-25.0 kg/m^2**					
	None	159,861 (82.35)	2.4	1.00 (reference)	1.00 (reference)

	Patients with COVID-19	34,255 (17.65)	2.8	1.14 (0.78 to 1.68)	1.15 (0.78 to 1.69)
**BMI, ≥25.0 kg/m^2**					
	None	289,452 (82.43)	2.5	1.00 (reference)	1.00 (reference)
	Patients with COVID-19	61,718 (17.57)	2.9	1.12 (0.85 to 1.49)	1.11 (0.84 to 1.47)
**Drinker**					
	None	293,797 (82.42)	2.4	1.00 (reference)	1.00 (reference)
	Patients with COVID-19	62,665 (17.58)	2.6	1.08 (0.81 to 1.45)	1.08 (0.80 to 1.44)
**Non-drinker**					
	None	394,032 (82.24)	2.7	1.00 (reference)	1.00 (reference)
	Patients with COVID-19	85,120 (17.76)	3.3	1.19 (0.95 to 1.50)	1.19 (0.95 to 1.50)
**Sufficient physical activity**					
	None	340,507 (82.39)	2.6	1.00 (reference)	1.00 (reference)
	Patients with COVID-19	72,760 (17.61)	2.6	0.98 (0.75 to 1.29)	0.98 (0.75 to 1.29)
**Insufficient physical activity**					
	None	347,204 (82.24)	2.5	1.00 (reference)	1.00 (reference)
	Patients with COVID-19	74,978 (17.76)	3.3	**1.32 (1.03 to 1.68)**	**1.31 (1.03 to 1.67)**
**Smoker (current and ex-)**					
	None	251,296 (82.46)	2.1	1.00 (reference)	1.00 (reference)
	Patients with COVID-19	53,445 (17.54)	2.1	1.00 (0.71 to 1.42)	1.00 (0.71 to 1.42)
**Non-smoker**					
	None	436,584 (82.23)	2.8	1.00 (reference)	1.00 (reference)
	Patients with COVID-19	94,341 (17.77)	3.4	1.21 (0.98 to 1.49)	1.21 (0.98 to 1.49)

**Rural residence**					
	None	368,924 (82.35)	2.9	1.00 (reference)	1.00 (reference)
	Patients with COVID-19	79,097 (17.65)	3.4	1.18 (0.93 to 1.48)	1.17 (0.93 to 1.48)
**Urban residence**					
	None	319,416 (82.29)	2.2	1.00 (reference)	1.00 (reference)
	Patients with COVID-19	68,727 (17.71)	2.5	1.11 (0.84 to 1.48)	1.11 (0.84 to 1.47)
**Strain type (original)**‡					
	None	220,904 (82.49)	2.9	1.00 (reference)	1.00 (reference)
	Patients with COVID-19	46,900 (17.51)	3.5	1.22 (1.00 to 1.48)	1.21 (0.99 to 1.48)
**Strain type (delta)**‡					
	None	467,436 (82.24)	1.9	1.00 (reference)	1.00 (reference)
	Patients with COVID-19	100,924 (17.76)	1.7	0.92 (0.60 to 1.40)	0.92 (0.60 to 1.40)

BMI, body mass index; CCI, Charlson comorbidity index; CI, confidence interval; HR, hazard ratio.

* Incidence rate is expressed as per 1,000 person-years.

‡ We considered the original SARS-CoV-2 if the initial infection was before July 31, 2021, and the delta variant was from August 1, 2021.

The data in bold indicate significant differences (P < 0.05).

[a] **Model 1**: Adjusted for age (20–39, 40–59, and ≥60 years) and sex.

[b] **Model 2**: Adjusted for age (20–39, 40–59, and ≥60 years); sex; household income (low income, middle income, and high income); region of residence (urban and rural); Charlson comorbidity index (0, 1, and ≥2); BMI (underweight [<18.5 kg/m^2], normal [18.5–23.0 kg/m^2], overweight [23.0–25.0 kg/m^2], obese [≥25.0 kg/m^2], and unknown); blood pressure (systolic blood pressure <140 mmHg and diastolic blood pressure <90 mmHg, systolic blood pressure ≥140 mmHg or diastolic blood pressure ≥90 mmHg, and unknown); fasting blood glucose (<100, ≥100 mg/dL, and unknown); serum total cholesterol (<200, 200–240, ≥240 mg/dL, and unknown); glomerular filtration rate (<60, 60−90, ≥90 mL/min/1.73 m^2, and unknown); smoking status (non-, ex-, current smoker, and unknown);

alcoholic drinks (<1, 1–2, 3–4, ≥5 days per week, and unknown); aerobic physical activity (sufficient, insufficient, and unknown); previous history of cardiovascular disease, chronic kidney disease, and chronic obstructive pulmonary disease; history of medication use for diabetes mellitus, dyslipidemia, and hypertension; and missing indicators (BMI missing indicator [yes or no], blood pressure missing indicator [yes or no], fasting blood glucose missing indicator [yes or no], serum total cholesterol missing indicator [yes or no], glomerular filtration rate missing indicator [yes or no], smoking status missing indicator [yes or no], alcoholic drinks missing indicator [yes or no], and aerobic physical activity missing indicator [yes or no]).

Table S15. Stratification analysis for the long-term sequelae risk of **incident food allergy** following COVID-19 diagnosis in the propensity score-matched main cohort (South Korea; n=836,164).

Parameter	Exposure	n (%)	Incidence rate*	HR (95% CI)	
				Model 1 [a]	Model 2 [b]
**Total**					
	None	688,340 (82.32)	3.6	1.00 (reference)	1.00 (reference)
	Patients with COVID-19	147,824 (17.68)	3.0	0.85 (0.71 to 1.01)	0.85 (0.71 to 1.00)
**Male**					
	None	382,175 (82.50)	3.2	1.00 (reference)	1.00 (reference)
	Patients with COVID-19	81,075 (17.50)	2.5	0.80 (0.62 to 1.04)	0.80 (0.62 to 1.04)
**Female**					
	None	306,165 (82.10)	4.1	1.00 (reference)	1.00 (reference)
	Patients with COVID-19	66,749 (17.90)	3.7	0.88 (0.70 to 1.12)	0.88 (0.70 to 1.12)
**Age 20–39 years**					
	None	173,290 (82.39)	2.1	1.00 (reference)	1.00 (reference)
	Patients with COVID-19	37,030 (17.61)	1.6	0.77 (0.49 to 1.22)	0.77 (0.49 to 1.22)
**Age 40–59 years**					
	None	299,203 (82.51)	3.4	1.00 (reference)	1.00 (reference)
	Patients with COVID-19	63,421 (17.49)	2.6	**0.75 (0.57 to 0.99)**	**0.75 (0.57 to 0.98)**
**Age ≥60 years**					
	None	215,847 (82.00)	5.3	1.00 (reference)	1.00 (reference)
	Patients with COVID-19	47,373 (18.00)	5.2	0.97 (0.76 to 1.26)	0.98 (0.76 to 1.26)
**Low income**					
	None	298,798 (82.41)	3.8	1.00 (reference)	1.00 (reference)

	Patients with COVID-19	63,769 (17.59)	3.5	0.93 (0.73 to 1.20)	0.93 (0.73 to 1.19)
**Middle income**					
	None	262,108 (82.38)	3.5	1.00 (reference)	1.00 (reference)
	Patients with COVID-19	56,080 (17.62)	2.8	0.78 (0.58 to 1.05)	0.78 (0.58 to 1.04)
**High income**					
	None	127,434 (82.00)	3.3	1.00 (reference)	1.00 (reference)
	Patients with COVID-19	27,975 (18.00)	2.5	0.76 (0.49 to 1.16)	0.75 (0.48 to 1.15)
**CCI, 0 score**					
	None	597,115 (82.47)	3.3	1.00 (reference)	1.00 (reference)
	Patients with COVID-19	126,952 (17.53)	2.9	0.88 (0.72 to 1.06)	0.88 (0.72 to 1.06)
**CCI, 1 scores**					
	None	63,117 (82.15)	5.9	1.00 (reference)	1.00 (reference)
	Patients with COVID-19	13,712 (17.85)	3.8	0.65 (0.41 to 1.04)	0.65 (0.41 to 1.04)
**CCI, ≥2 scores**					
	None	28,108 (79.70)	4.8	1.00 (reference)	1.00 (reference)
	Patients with COVID-19	7,160 (20.30)	4.7	0.99 (0.52 to 1.91)	0.95 (0.49 to 1.84)
**BMI, <18.5 kg/m^2**					
	None	17,997 (81.42)	2.9	1.00 (reference)	1.00 (reference)
	Patients with COVID-19	4,108 (18.58)	5.9	2.01 (0.87 to 4.62)	2.14 (0.92 to 4.99)
**BMI, 18.5-23.0 kg/m^2**					
	None	220,919 (82.24)	3.3	1.00 (reference)	1.00 (reference)
	Patients with COVID-19	47,707 (17.76)	2.9	0.89 (0.65 to 1.21)	0.88 (0.65 to 1.20)
**BMI, 23.0-25.0 kg/m^2**					
	None	159,861 (82.35)	3.9	1.00 (reference)	1.00 (reference)

	Patients with COVID-19	34,255 (17.65)	3.0	0.77 (0.54 to 1.10)	0.77 (0.54 to 1.10)
**BMI, ≥25.0 kg/m^2**					
	None	289,452 (82.43)	3.7	1.00 (reference)	1.00 (reference)
	Patients with COVID-19	61,718 (17.57)	2.9	0.80 (0.61 to 1.05)	0.80 (0.61 to 1.05)
**Drinker**					
	None	293,797 (82.42)	3.2	1.00 (reference)	1.00 (reference)
	Patients with COVID-19	62,665 (17.58)	2.5	0.78 (0.58 to 1.04)	0.77 (0.58 to 1.03)
**Non-drinker**					
	None	394,032 (82.24)	3.9	1.00 (reference)	1.00 (reference)
	Patients with COVID-19	85,120 (17.76)	3.4	0.89 (0.72 to 1.10)	0.89 (0.71 to 1.10)
**Sufficient physical activity**					
	None	340,507 (82.39)	3.6	1.00 (reference)	1.00 (reference)
	Patients with COVID-19	72,760 (17.61)	3.2	0.87 (0.68 to 1.11)	0.87 (0.68 to 1.11)
**Insufficient physical activity**					
	None	347,204 (82.24)	3.6	1.00 (reference)	1.00 (reference)
	Patients with COVID-19	74,978 (17.76)	2.9	0.82 (0.64 to 1.06)	0.82 (0.64 to 1.05)
**Smoker (current and ex-)**					
	None	251,296 (82.46)	3.3	1.00 (reference)	1.00 (reference)
	Patients with COVID-19	53,445 (17.54)	2.5	0.78 (0.57 to 1.06)	0.78 (0.57 to 1.06)
**Non-smoker**					
	None	436,584 (82.23)	3.8	1.00 (reference)	1.00 (reference)
	Patients with COVID-19	94,341 (17.77)	3.3	0.88 (0.72 to 1.08)	0.88 (0.72 to 1.08)
**Rural residence**					

	None	368,924 (82.35)	3.7	1.00 (reference)	1.00 (reference)
	Patients with COVID-19	79,097 (17.65)	3.4	0.92 (0.73 to 1.16)	0.92 (0.73 to 1.15)
**Urban residence**					
	None	319,416 (82.29)	3.5	1.00 (reference)	1.00 (reference)
	Patients with COVID-19	68,727 (17.71)	2.7	**0.76 (0.58 to 0.99)**	**0.76 (0.58 to 0.99)**
**Strain type (original)**					
	None	220,904 (82.49)	3.9	1.00 (reference)	1.00 (reference)
	Patients with COVID-19	46,900 (17.51)	3.4	0.88 (0.73 to 1.07)	0.88 (0.73 to 1.07)
**Strain type (delta)**					
	None	467,436 (82.24)	2.9	1.00 (reference)	1.00 (reference)
	Patients with COVID-19	100,924 (17.76)	2.1	0.73 (0.51 to 1.06)	0.73 (0.50 to 1.06)

BMI, body mass index; CCI, Charlson comorbidity index; CI, confidence interval; HR, hazard ratio.

* Incidence rate is expressed as per 1,000 person-years.

‡ We considered the original SARS-CoV-2 if the initial infection was before July 31, 2021, and the delta variant was from August 1, 2021.

The data in bold indicate significant differences ($P < 0.05$).

[a] **Model 1**: Adjusted for age (20–39, 40–59, and ≥60 years) and sex.

[b] **Model 2**: Adjusted for age (20–39, 40–59, and ≥60 years); sex; household income (low income, middle income, and high income); region of residence (urban and rural); Charlson comorbidity index (0, 1, and ≥2); BMI (underweight [<18.5 kg/m^2], normal [18.5–23.0 kg/m^2], overweight [23.0–25.0 kg/m^2], obese [≥25.0 kg/m^2], and unknown); blood pressure (systolic blood pressure <140 mmHg and diastolic blood pressure <90 mmHg, systolic blood pressure ≥140 mmHg or diastolic blood pressure ≥90 mmHg, and unknown); fasting blood glucose (<100, ≥100 mg/dL, and unknown); serum total cholesterol (<200, 200–240, ≥240 mg/dL, and unknown); glomerular filtration rate (<60, 60–90, ≥90 mL/min/1.73 m^2, and unknown); smoking status (non-, ex-, current smoker, and unknown); alcoholic drinks (<1, 1–2, 3–4, ≥5 days per week, and unknown); aerobic physical activity (sufficient, insufficient, and unknown); previous history of cardiovascular disease, chronic kidney disease, and chronic obstructive pulmonary disease; history of medication use for diabetes mellitus, dyslipidemia, and hypertension; and missing indicators (BMI missing indicator [yes or no], blood pressure missing indicator [yes or no], fasting blood glucose missing indicator

[yes or no], serum total cholesterol missing indicator [yes or no], glomerular filtration rate missing indicator [yes or no], smoking status missing indicator [yes or no], alcoholic drinks missing indicator [yes or no], and aerobic physical activity missing indicator [yes or no]).

Table S16. Stratification analysis for the long-term sequelae risk of **incident allergic diseases** following COVID-19 diagnosis in the propensity score-matched replication cohort A (Japan; n=2,541,021).

Parameter	Exposure	n (%)	Incidence rate*	HR (95% CI)	
				Model 1 [a]	Model 2 [b]
**Total**					
	None	1,998,524 (78.65)	27.4	1.00 (reference)	1.00 (reference)
	Patients with COVID-19	542,497 (21.35)	75.1	**2.65 (2.61 to 2.69)**	**2.56 (2.52 to 2.59)**
**Male**					
	None	1,352,980 (79.00)	23.8	1.00 (reference)	1.00 (reference)
	Patients with COVID-19	359,753 (21.00)	66.7	**2.74 (2.69 to 2.80)**	**2.63 (2.58 to 2.68)**
**Female**					
	None	645,544 (77.94)	35.0	1.00 (reference)	1.00 (reference)
	Patients with COVID-19	182,744 (22.06)	91.9	**2.52 (2.46 to 2.58)**	**2.45 (2.39 to 2.50)**
**Age 20–39 years**					
	None	489,756 (72.28)	30.9	1.00 (reference)	1.00 (reference)
	Patients with COVID-19	187,828 (27.72)	85.9	**2.71 (2.64 to 2.78)**	**2.67 (2.60 to 2.74)**
**Age 40–59 years**					
	None	1,238,373 (81.05)	25.3	1.00 (reference)	1.00 (reference)
	Patients with COVID-19	289,616 (18.95)	69.5	**2.72 (2.67 to 2.77)**	**2.60 (2.54 to 2.65)**
**Age ≥60 years**					
	None	270,395 (80.61)	30.4	1.00 (reference)	1.00 (reference)
	Patients with COVID-19	65,053 (19.39)	68.7	**2.26 (2.17 to 2.35)**	**2.12 (2.03 to 2.21)**
**CCI, 0 score**					
	None	1,976,142 (79.13)	27.3	1.00 (reference)	1.00 (reference)

	Patients with COVID-19	521,111 (20.87)	74.7	**2.64 (2.60 to 2.68)**	**2.56 (2.52 to 2.60)**
**CCI, 1 scores**					
	None	7,141 (52.03)	36.6	1.00 (reference)	1.00 (reference)
	Patients with COVID-19	6,583 (47.97)	83.7	**2.40 (2.05 to 2.80)**	**2.39 (2.04 to 2.80)**
**CCI, ≥2 scores**					
	None	15,241 (50.73)	32.8	1.00 (reference)	1.00 (reference)
	Patients with COVID-19	14,803 (49.27)	85.0	**2.56 (2.30 to 2.85)**	**2.40 (2.15 to 2.67)**
**BMI, <18.5 kg/m^2**					
	None	146,377 (77.43)	29.3	1.00 (reference)	1.00 (reference)
	Patients with COVID-19	42,662 (22.57)	83.3	**2.72 (2.59 to 2.86)**	**2.63 (2.50 to 2.77)**
**BMI, 18.5-23.0 kg/m^2**					
	None	864,919 (78.40)	28.0	1.00 (reference)	1.00 (reference)
	Patients with COVID-19	238,313 (21.60)	76.6	**2.61 (2.56 to 2.67)**	**2.54 (2.48 to 2.60)**
**BMI, 23.0-25.0 kg/m^2**					
	None	396,921 (79.36)	26.8	1.00 (reference)	1.00 (reference)
	Patients with COVID-19	103,239 (20.64)	72.6	**2.64 (2.55 to 2.73)**	**2.55 (2.46 to 2.64)**
**BMI, ≥25.0 kg/m^2**					
	None	580,305 (78.80)	26.9	1.00 (reference)	1.00 (reference)
	Patients with COVID-19	156,101 (21.20)	72.2	**2.65 (2.58 to 2.72)**	**2.53 (2.46 to 2.60)**
**Non-drinker**					
	None	774,477 (78.85)	28.2	1.00 (reference)	1.00 (reference)
	Patients with COVID-19	207,717 (21.15)	81.4	**2.80 (2.73 to 2.86)**	**2.70 (2.63 to 2.76)**

**Drinker**					
	None	1,113,296 (78.72)	27.1	1.00 (reference)	1.00 (reference)
	Patients with COVID-19	300,882 (21.28)	71.0	**2.54 (2.49 to 2.59)**	**2.45 (2.40 to 2.50)**
**Sufficient physical activity**					
	None	435,493 (79.73)	26.8	1.00 (reference)	1.00 (reference)
	Patients with COVID-19	110,731 (20.27)	68.3	**2.50 (2.42 to 2.58)**	**2.41 (2.33 to 2.50)**
**Insufficient physical activity**					
	None	1,430,215 (78.51)	27.8	1.00 (reference)	1.00 (reference)
	Patients with COVID-19	391,479 (21.49)	77.1	**2.68 (2.63 to 2.72)**	**2.58 (2.54 to 2.63)**

BMI, body mass index; CCI, Charlson comorbidity index; CI, confidence interval; HR, hazard ratio.

* Incidence rate is expressed as per 1,000 person-years.

The data in bold indicate significant differences ($P < 0.05$).

[a] **Model 1**: Adjusted for age (20–39, 40–59, and ≥60 years) and sex.

[b] **Model 2**: Adjusted for age (20–39, 40–59, and ≥60 years); sex; Charlson comorbidity index (0, 1, and ≥ 2); BMI (underweight [<18.5 kg/m^2], normal [18.5−23.0 kg/m^2], overweight [23.0–25.0 kg/m^2], obese [≥25.0 kg/m^2], and unknown); blood pressure (systolic blood pressure <140 mmHg and diastolic blood pressure <90 mmHg, systolic blood pressure ≥140 mmHg or diastolic blood pressure ≥90 mmHg, and unknown); fasting blood glucose (<100, ≥100 mg/dL, and unknown); serum total cholesterol (<200, 200–240, ≥240 mg/dL, and unknown); glomerular filtration rate (<60, 60−90, ≥90 mL/min/1.73 m^2, and unknown); smoking status (non- and current smoker, and unknown); alcoholic drinks (<1, 1–2, 3–4, ≥5 days per week, and unknown); aerobic physical activity (sufficient, insufficient, and unknown); previous history of cardiovascular disease, chronic kidney disease, and chronic obstructive pulmonary disease; history of medication use for diabetes mellitus, dyslipidemia, and hypertension; and missing indicators (BMI missing indicator [yes or no], blood pressure missing indicator [yes or no], fasting blood glucose missing indicator [yes or no], serum total cholesterol missing indicator [yes or no], glomerular filtration rate missing indicator [yes or no], smoking status missing indicator [yes or no], alcoholic drinks missing indicator [yes or no], and aerobic physical activity missing indicator [yes or no]).

Table S17. Stratification analysis for the long-term sequelae risk of **incident asthma** following COVID-19 diagnosis in the propensity score-matched replication cohort A (Japan; n=2,541,021).

Parameter	Exposure	n (%)	Incidence rate*	HR (95% CI)	
				Model 1 [a]	Model 2 [b]
**Total**					
	None	1,998,524 (78.65)	2.7	1.00 (reference)	1.00 (reference)
	Patients with COVID-19	542,497 (21.35)	7.6	**2.77 (2.65 to 2.90)**	**2.63 (2.51 to 2.75)**
**Male**					
	None	1,352,980 (79.00)	2.3	1.00 (reference)	1.00 (reference)
	Patients with COVID-19	359,753 (21.00)	6.7	**2.81 (2.65 to 2.98)**	**2.66 (2.51 to 2.83)**
**Female**					
	None	645,544 (77.94)	3.4	1.00 (reference)	1.00 (reference)
	Patients with COVID-19	182,744 (22.06)	9.5	**2.70 (2.52 to 2.90)**	**2.57 (2.39 to 2.76)**
**Age 20–39 years**					
	None	489,756 (72.28)	3.0	1.00 (reference)	1.00 (reference)
	Patients with COVID-19	187,828 (27.72)	9.1	**2.96 (2.74 to 3.20)**	**2.91 (2.70 to 3.15)**
**Age 40–59 years**					
	None	1,238,373 (81.05)	2.6	1.00 (reference)	1.00 (reference)
	Patients with COVID-19	289,616 (18.95)	7.1	**2.75 (2.58 to 2.92)**	**2.58 (2.42 to 2.74)**
**Age ≥60 years**					
	None	270,395 (80.61)	2.6	1.00 (reference)	1.00 (reference)
	Patients with COVID-19	65,053 (19.39)	6.1	**2.31 (2.02 to 2.64)**	**2.07 (1.80 to 2.38)**
**CCI, 0 score**					
	None	1,976,142 (79.13)	2.7	1.00 (reference)	1.00 (reference)
	Patients with COVID-19	521,111 (20.87)	7.6	**2.74 (2.62 to 2.87)**	**2.62 (2.51 to 2.75)**
**CCI, 1 scores**					

	None	7,141 (52.03)	3.8	1.00 (reference)	1.00 (reference)
	Patients with COVID-19	6,583 (47.97)	10.7	**3.07 (1.93 to 4.87)**	**2.93 (1.84 to 4.67)**
**CCI, ≥2 scores**					
	None	15,241 (50.73)	2.6	1.00 (reference)	1.00 (reference)
	Patients with COVID-19	14,803 (49.27)	7.8	**3.00 (2.07 to 4.34)**	**2.73 (1.87 to 3.98)**
**BMI, <18.5 kg/m^2**					
	None	146,377 (77.43)	2.9	1.00 (reference)	1.00 (reference)
	Patients with COVID-19	42,662 (22.57)	8.6	**2.88 (2.47 to 3.36)**	**2.72 (2.33 to 3.18)**
**BMI, 18.5-23.0 kg/m^2**					
	None	864,919 (78.40)	2.5	1.00 (reference)	1.00 (reference)
	Patients with COVID-19	238,313 (21.60)	7.7	**2.95 (2.76 to 3.17)**	**2.83 (2.64 to 3.04)**
**BMI, 23.0-25.0 kg/m^2**					
	None	396,921 (79.36)	2.6	1.00 (reference)	1.00 (reference)
	Patients with COVID-19	103,239 (20.64)	7.0	**2.60 (2.34 to 2.89)**	**2.49 (2.24 to 2.77)**
**BMI, ≥25.0 kg/m^2**					
	None	580,305 (78.80)	2.9	1.00 (reference)	1.00 (reference)
	Patients with COVID-19	156,101 (21.20)	7.7	**2.56 (2.36 to 2.78)**	**2.41 (2.21 to 2.61)**
**Non-drinker**					
	None	774,477 (78.85)	2.9	1.00 (reference)	1.00 (reference)
	Patients with COVID-19	207,717 (21.15)	8.8	**2.95 (2.76 to 3.16)**	**2.80 (2.61 to 3.00)**
**Drinker**					
	None	1,113,296 (78.72)	2.6	1.00 (reference)	1.00 (reference)
	Patients with COVID-19	300,882 (21.28)	6.8	**2.61 (2.45 to 2.78)**	**2.47 (2.32 to 2.63)**
**Sufficient physical activity**					
	None	435,493 (79.73)	2.4	1.00 (reference)	1.00 (reference)

	Patients with COVID-19	110,731 (20.27)	5.8	**2.42 (2.16 to 2.70)**	**2.29 (2.04 to 2.56)**
**Insufficient physical activity**					
	None	1,430,215 (78.51)	2.8	1.00 (reference)	1.00 (reference)
	Patients with COVID-19	391,479 (21.49)	8.1	**2.82 (2.68 to 2.97)**	**2.68 (2.54 to 2.83)**

BMI, body mass index; CCI, Charlson comorbidity index; CI, confidence interval; HR, hazard ratio.

* Incidence rate is expressed as per 1,000 person-years.

The data in bold indicate significant differences ($P < 0.05$).

[a] **Model 1**: Adjusted for age (20–39, 40–59, and ≥60 years) and sex.

[b] **Model 2**: Adjusted for age (20–39, 40–59, and ≥60 years); sex; Charlson comorbidity index (0, 1, and ≥ 2); BMI (underweight [<18.5 kg/m^2], normal [18.5–23.0 kg/m^2], overweight [23.0–25.0 kg/m^2], obese [≥25.0 kg/m^2], and unknown); blood pressure (systolic blood pressure <140 mmHg and diastolic blood pressure <90 mmHg, systolic blood pressure ≥140 mmHg or diastolic blood pressure ≥90 mmHg, and unknown); fasting blood glucose (<100, ≥100 mg/dL, and unknown); serum total cholesterol (<200, 200–240, ≥240 mg/dL, and unknown); glomerular filtration rate (<60, 60–90, ≥90 mL/min/1.73 m^2, and unknown); smoking status (non- and current smoker, and unknown); alcoholic drinks (<1, 1–2, 3–4, ≥5 days per week, and unknown); aerobic physical activity (sufficient, insufficient, and unknown); previous history of cardiovascular disease, chronic kidney disease, and chronic obstructive pulmonary disease; history of medication use for diabetes mellitus, dyslipidemia, and hypertension; and missing indicators (BMI missing indicator [yes or no], blood pressure missing indicator [yes or no], fasting blood glucose missing indicator [yes or no], serum total cholesterol missing indicator [yes or no], glomerular filtration rate missing indicator [yes or no], smoking status missing indicator [yes or no], alcoholic drinks missing indicator [yes or no], and aerobic physical activity missing indicator [yes or no]).

Table S18. Stratification analysis for the long-term sequelae risk of **incident allergic rhinitis** following COVID-19 diagnosis in the propensity score-matched replication cohort A (Japan; n=2,541,021).

Parameter	Exposure	n (%)	Incidence rate*	HR (95% CI)	
				Model 1 [a]	Model 2 [b]
**Total**					
	None	1,998,524 (78.65)	20.4	1.00 (reference)	1.00 (reference)
	Patients with COVID-19	542,497 (21.35)	62.6	**2.98 (2.93 to 3.03)**	**2.88 (2.83 to 2.93)**
**Male**					
	None	1,352,980 (79.00)	17.9	1.00 (reference)	1.00 (reference)
	Patients with COVID-19	359,753 (21.00)	55.7	**3.05 (2.99 to 3.12)**	**2.94 (2.88 to 3.01)**
**Female**					
	None	645,544 (77.94)	25.6	1.00 (reference)	1.00 (reference)
	Patients with COVID-19	182,744 (22.06)	76.1	**2.86 (2.79 to 2.94)**	**2.79 (2.72 to 2.86)**
**Age 20–39 years**					
	None	489,756 (72.28)	21.9	1.00 (reference)	1.00 (reference)
	Patients with COVID-19	187,828 (27.72)	72.2	**3.20 (3.11 to 3.29)**	**3.16 (3.07 to 3.25)**
**Age 40–59 years**					
	None	1,238,373 (81.05)	18.9	1.00 (reference)	1.00 (reference)
	Patients with COVID-19	289,616 (18.95)	57.7	**3.02 (2.95 to 3.09)**	**2.90 (2.83 to 2.96)**
**Age ≥60 years**					
	None	270,395 (80.61)	24.2	1.00 (reference)	1.00 (reference)
	Patients with COVID-19	65,053 (19.39)	56.5	**2.33 (2.23 to 2.44)**	**2.22 (2.12 to 2.32)**
**CCI, 0 score**					
	None	1,976,142 (79.13)	20.4	1.00 (reference)	1.00 (reference)
	Patients with COVID-19	521,111 (20.87)	62.5	**2.97 (2.92 to 3.02)**	**2.89 (2.84 to 2.94)**

**CCI, 1 scores**					
	None	7,141 (52.03)	27.6	1.00 (reference)	1.00 (reference)
	Patients with COVID-19	6,583 (47.97)	67.2	**2.55 (2.14 to 3.04)**	**2.56 (2.14 to 3.05)**
**CCI, ≥2 scores**					
	None	15,241 (50.73)	23.3	1.00 (reference)	1.00 (reference)
	Patients with COVID-19	14,803 (49.27)	63.5	**2.69 (2.37 to 3.05)**	**2.53 (2.22 to 2.88)**
**BMI, <18.5 kg/m^2**					
	None	146,377 (77.43)	20.1	1.00 (reference)	1.00 (reference)
	Patients with COVID-19	42,662 (22.57)	68.0	**3.24 (3.06 to 3.43)**	**3.13 (2.96 to 3.32)**
**BMI, 18.5-23.0 kg/m^2**					
	None	864,919 (78.40)	20.5	1.00 (reference)	1.00 (reference)
	Patients with COVID-19	238,313 (21.60)	63.7	**2.98 (2.91 to 3.05)**	**2.91 (2.83 to 2.98)**
**BMI, 23.0-25.0 kg/m^2**					
	None	396,921 (79.36)	20.3	1.00 (reference)	1.00 (reference)
	Patients with COVID-19	103,239 (20.64)	61.1	**2.94 (2.83 to 3.05)**	**2.86 (2.75 to 2.97)**
**BMI, ≥25.0 kg/m^2**					
	None	580,305 (78.80)	20.8	1.00 (reference)	1.00 (reference)
	Patients with COVID-19	156,101 (21.20)	60.3	**2.87 (2.78 to 2.95)**	**2.76 (2.67 to 2.84)**
**Non-drinker**					
	None	774,477 (78.85)	20.5	1.00 (reference)	1.00 (reference)
	Patients with COVID-19	207,717 (21.15)	67.7	**3.20 (3.12 to 3.28)**	**3.10 (3.02 to 3.18)**
**Drinker**					
	None	1,113,296 (78.72)	20.6	1.00 (reference)	1.00 (reference)
	Patients with COVID-19	300,882 (21.28)	59.1	**2.80 (2.74 to 2.86)**	**2.71 (2.65 to 2.77)**

**Sufficient physical activity**					
	None	435,493 (79.73)	20.4	1.00 (reference)	1.00 (reference)
	Patients with COVID-19	110,731 (20.27)	57.2	**2.76 (2.66 to 2.86)**	**2.68 (2.58 to 2.78)**
**Insufficient physical activity**					
	None	1,430,215 (78.51)	20.6	1.00 (reference)	1.00 (reference)
	Patients with COVID-19	391,479 (21.49)	64.2	**3.01 (2.95 to 3.07)**	**2.91 (2.86 to 2.97)**

BMI, body mass index; CCI, Charlson comorbidity index; CI, confidence interval; HR, hazard ratio.

* Incidence rate is expressed as per 1,000 person-years.

The data in bold indicate significant differences ($P < 0.05$).

[a] **Model 1**: Adjusted for age (20–39, 40–59, and ≥60 years) and sex.

[b] **Model 2**: Adjusted for age (20–39, 40–59, and ≥60 years); sex; Charlson comorbidity index (0, 1, and ≥ 2); BMI (underweight [<18.5 kg/m^2], normal [18.5–23.0 kg/m^2], overweight [23.0–25.0 kg/m^2], obese [≥25.0 kg/m^2], and unknown); blood pressure (systolic blood pressure <140 mmHg and diastolic blood pressure <90 mmHg, systolic blood pressure ≥140 mmHg or diastolic blood pressure ≥90 mmHg, and unknown); fasting blood glucose (<100, ≥100 mg/dL, and unknown); serum total cholesterol (<200, 200–240, ≥240 mg/dL, and unknown); glomerular filtration rate (<60, 60–90, ≥90 mL/min/1.73 m^2, and unknown); smoking status (non- and current smoker, and unknown); alcoholic drinks (<1, 1–2, 3–4, ≥5 days per week, and unknown); aerobic physical activity (sufficient, insufficient, and unknown); previous history of cardiovascular disease, chronic kidney disease, and chronic obstructive pulmonary disease; history of medication use for diabetes mellitus, dyslipidemia, and hypertension; and missing indicators (BMI missing indicator [yes or no], blood pressure missing indicator [yes or no], fasting blood glucose missing indicator [yes or no], serum total cholesterol missing indicator [yes or no], glomerular filtration rate missing indicator [yes or no], smoking status missing indicator [yes or no], alcoholic drinks missing indicator [yes or no], and aerobic physical activity missing indicator [yes or no]).

Table S19. Stratification analysis for the long-term sequelae risk of **incident atopic dermatitis** following COVID-19 diagnosis in the propensity score-matched replication cohort A (Japan; n=2,541,021).

Parameter	Exposure	n (%)	Incidence rate*	HR (95% CI)	
				Model 1 [a]	Model 2 [b]
**Total**					
	None	1,998,524 (78.65)	5.2	1.00 (reference)	1.00 (reference)
	Patients with COVID-19	542,497 (21.35)	6.8	**1.25 (1.20 to 1.30)**	**1.21 (1.16 to 1.26)**
**Male**					
	None	1,352,980 (79.00)	4.2	1.00 (reference)	1.00 (reference)
	Patients with COVID-19	359,753 (21.00)	5.7	**1.31 (1.24 to 1.38)**	**1.26 (1.19 to 1.33)**
**Female**					
	None	645,544 (77.94)	7.2	1.00 (reference)	1.00 (reference)
	Patients with COVID-19	182,744 (22.06)	9.0	**1.18 (1.11 to 1.26)**	**1.16 (1.09 to 1.23)**
**Age 20–39 years**					
	None	489,756 (72.28)	7.5	1.00 (reference)	1.00 (reference)
	Patients with COVID-19	187,828 (27.72)	8.3	**1.09 (1.02 to 1.16)**	**1.08 (1.01 to 1.15)**
**Age 40–59 years**					
	None	1,238,373 (81.05)	4.6	1.00 (reference)	1.00 (reference)
	Patients with COVID-19	289,616 (18.95)	6.2	**1.35 (1.27 to 1.44)**	**1.30 (1.23 to 1.38)**
**Age ≥60 years**					
	None	270,395 (80.61)	3.7	1.00 (reference)	1.00 (reference)
	Patients with COVID-19	65,053 (19.39)	5.4	**1.50 (1.31 to 1.71)**	**1.41 (1.23 to 1.62)**
**CCI, 0 score**					
	None	1,976,142 (79.13)	5.2	1.00 (reference)	1.00 (reference)
	Patients with COVID-19	521,111 (20.87)	6.7	**1.22 (1.17 to 1.27)**	**1.20 (1.15 to 1.25)**

**CCI, 1 scores**					
	None	7,141 (52.03)	4.5	1.00 (reference)	1.00 (reference)
	Patients with COVID-19	6,583 (47.97)	7.1	**1.70 (1.07 to 2.71)**	**1.66 (1.04 to 2.66)**
**CCI, ≥2 scores**					
	None	15,241 (50.73)	6.0	1.00 (reference)	1.00 (reference)
	Patients with COVID-19	14,803 (49.27)	9.6	**1.59 (1.21 to 2.08)**	**1.44 (1.10 to 1.90)**
**BMI, <18.5 kg/m^2**					
	None	146,377 (77.43)	7.4	1.00 (reference)	1.00 (reference)
	Patients with COVID-19	42,662 (22.57)	9.2	**1.18 (1.03 to 1.34)**	**1.16 (1.02 to 1.33)**
**BMI, 18.5-23.0 kg/m^2**					
	None	864,919 (78.40)	5.8	1.00 (reference)	1.00 (reference)
	Patients with COVID-19	238,313 (21.60)	7.4	**1.19 (1.12 to 1.26)**	**1.16 (1.09 to 1.24)**
**BMI, 23.0-25.0 kg/m^2**					
	None	396,921 (79.36)	4.7	1.00 (reference)	1.00 (reference)
	Patients with COVID-19	103,239 (20.64)	6.5	**1.30 (1.18 to 1.44)**	**1.26 (1.14 to 1.39)**
**BMI, ≥25.0 kg/m^2**					
	None	580,305 (78.80)	4.0	1.00 (reference)	1.00 (reference)
	Patients with COVID-19	156,101 (21.20)	5.7	**1.36 (1.25 to 1.48)**	**1.30 (1.19 to 1.41)**
**Non-drinker**					
	None	774,477 (78.85)	5.6	1.00 (reference)	1.00 (reference)
	Patients with COVID-19	207,717 (21.15)	7.3	**1.22 (1.15 to 1.31)**	**1.19 (1.12 to 1.27)**
**Drinker**					
	None	1,113,296 (78.72)	4.9	1.00 (reference)	1.00 (reference)
	Patients with COVID-19	300,882 (21.28)	6.6	**1.27 (1.20 to 1.35)**	**1.24 (1.17 to 1.31)**

**Sufficient physical activity**					
	None	435,493 (79.73)	4.7	1.00 (reference)	1.00 (reference)
	Patients with COVID-19	110,731 (20.27)	6.1	**1.23 (1.12 to 1.36)**	**1.20 (1.09 to 1.33)**
**Insufficient physical activity**					
	None	1,430,215 (78.51)	5.3	1.00 (reference)	1.00 (reference)
	Patients with COVID-19	391,479 (21.49)	7.1	**1.26 (1.20 to 1.32)**	**1.22 (1.17 to 1.29)**

BMI, body mass index; CCI, Charlson comorbidity index; CI, confidence interval; HR, hazard ratio.

* Incidence rate is expressed as per 1,000 person-years.

The data in bold indicate significant differences ($P < 0.05$).

[a] **Model 1**: Adjusted for age (20–39, 40–59, and ≥60 years) and sex.

[b] **Model 2**: Adjusted for age (20–39, 40–59, and ≥60 years); sex; Charlson comorbidity index (0, 1, and ≥ 2); BMI (underweight [<18.5 kg/m^2], normal [18.5–23.0 kg/m^2], overweight [23.0–25.0 kg/m^2], obese [≥25.0 kg/m^2], and unknown); blood pressure (systolic blood pressure <140 mmHg and diastolic blood pressure <90 mmHg, systolic blood pressure ≥140 mmHg or diastolic blood pressure ≥90 mmHg, and unknown); fasting blood glucose (<100, ≥100 mg/dL, and unknown); serum total cholesterol (<200, 200–240, ≥240 mg/dL, and unknown); glomerular filtration rate (<60, 60–90, ≥90 mL/min/1.73 m^2, and unknown); smoking status (non- and current smoker, and unknown); alcoholic drinks (<1, 1–2, 3–4, ≥5 days per week, and unknown); aerobic physical activity (sufficient, insufficient, and unknown); previous history of cardiovascular disease, chronic kidney disease, and chronic obstructive pulmonary disease; history of medication use for diabetes mellitus, dyslipidemia, and hypertension; and missing indicators (BMI missing indicator [yes or no], blood pressure missing indicator [yes or no], fasting blood glucose missing indicator [yes or no], serum total cholesterol missing indicator [yes or no], glomerular filtration rate missing indicator [yes or no], smoking status missing indicator [yes or no], alcoholic drinks missing indicator [yes or no], and aerobic physical activity missing indicator [yes or no]).

Table S20. Stratification analysis for the long-term sequelae risk of **incident food allergy** following COVID-19 diagnosis in the propensity score-matched replication cohort A (Japan; n=2,541,021).

Parameter	Exposure	n (%)	Incidence rate*	HR (95% CI)	
				Model 1 [a]	Model 2 [b]
**Total**					
	None	1,998,524 (78.65)	1.3	1.00 (reference)	1.00 (reference)
	Patients with COVID-19	542,497 (21.35)	2.7	**2.10 (1.95 to 2.25)**	**1.84 (1.71 to 1.98)**
**Male**					
	None	1,352,980 (79.00)	1.0	1.00 (reference)	1.00 (reference)
	Patients with COVID-19	359,753 (21.00)	2.5	**2.46 (2.24 to 2.70)**	**2.09 (1.89 to 2.30)**
**Female**					
	None	645,544 (77.94)	1.9	1.00 (reference)	1.00 (reference)
	Patients with COVID-19	182,744 (22.06)	3.2	**1.70 (1.52 to 1.90)**	**1.56 (1.40 to 1.75)**
**Age 20–39 years**					
	None	489,756 (72.28)	1.3	1.00 (reference)	1.00 (reference)
	Patients with COVID-19	187,828 (27.72)	2.3	**1.74 (1.52 to 1.99)**	**1.65 (1.44 to 1.88)**
**Age 40–59 years**					
	None	1,238,373 (81.05)	1.2	1.00 (reference)	1.00 (reference)
	Patients with COVID-19	289,616 (18.95)	2.7	**2.22 (2.02 to 2.45)**	**1.94 (1.76 to 2.14)**
**Age ≥60 years**					
	None	270,395 (80.61)	1.6	1.00 (reference)	1.00 (reference)
	Patients with COVID-19	65,053 (19.39)	3.8	**2.37 (2.00 to 2.81)**	**1.90 (1.59 to 2.28)**
**CCI, 0 score**					
	None	1,976,142 (79.13)	1.3	1.00 (reference)	1.00 (reference)
	Patients with COVID-19	521,111 (20.87)	2.5	**1.95 (1.80 to 2.10)**	**1.80 (1.67 to 1.95)**
**CCI, 1 scores**					
	None	7,141 (52.03)	3.1	1.00 (reference)	1.00 (reference)
	Patients with COVID-19	6,583 (47.97)	4.7	**1.54 (0.88 to 2.70)**	**1.48 (0.84 to 2.61)**
**CCI, ≥2 scores**					

	None	15,241 (50.73)	2.9	1.00 (reference)	1.00 (reference)
	Patients with COVID-19	14,803 (49.27)	8.9	**3.04 (2.15 to 4.31)**	**2.83 (1.99 to 4.03)**
**BMI, <18.5 kg/m²**					
	None	146,377 (77.43)	1.7	1.00 (reference)	1.00 (reference)
	Patients with COVID-19	42,662 (22.57)	3.5	**2.13 (1.70 to 2.66)**	**1.86 (1.48 to 2.33)**
**BMI, 18.5-23.0 kg/m²**					
	None	864,919 (78.40)	1.4	1.00 (reference)	1.00 (reference)
	Patients with COVID-19	238,313 (21.60)	2.6	**1.84 (1.65 to 2.04)**	**1.63 (1.46 to 1.82)**
**BMI, 23.0-25.0 kg/m²**					
	None	396,921 (79.36)	1.2	1.00 (reference)	1.00 (reference)
	Patients with COVID-19	103,239 (20.64)	2.5	**2.13 (1.80 to 2.53)**	**1.88 (1.58 to 2.24)**
**BMI, ≥25.0 kg/m²**					
	None	580,305 (78.80)	1.1	1.00 (reference)	1.00 (reference)
	Patients with COVID-19	156,101 (21.20)	2.7	**2.51 (2.19 to 2.87)**	**2.17 (1.89 to 2.49)**
**Non-drinker**					
	None	774,477 (78.85)	1.5	1.00 (reference)	1.00 (reference)
	Patients with COVID-19	207,717 (21.15)	2.9	**1.97 (1.77 to 2.20)**	**1.69 (1.51 to 1.89)**
**Drinker**					
	None	1,113,296 (78.72)	1.2	1.00 (reference)	1.00 (reference)
	Patients with COVID-19	300,882 (21.28)	2.5	**2.16 (1.96 to 2.39)**	**1.92 (1.74 to 2.13)**
**Sufficient physical activity**					
	None	435,493 (79.73)	1.3	1.00 (reference)	1.00 (reference)
	Patients with COVID-19	110,731 (20.27)	2.8	**2.23 (1.90 to 2.60)**	**1.90 (1.62 to 2.23)**
**Insufficient physical activity**					
	None	1,430,215 (78.51)	1.3	1.00 (reference)	1.00 (reference)
	Patients with COVID-19	391,479 (21.49)	2.7	**2.02 (1.86 to 2.20)**	**1.78 (1.64 to 1.94)**

BMI, body mass index; CCI, Charlson comorbidity index; CI, confidence interval; HR, hazard ratio.

* Incidence rate is expressed as per 1,000 person-years.

The data in bold indicate significant differences ($P < 0.05$).

[a] **Model 1**: Adjusted for age (20–39, 40–59, and ≥60 years) and sex.

[b] **Model 2**: Adjusted for age (20–39, 40–59, and ≥60 years); sex; Charlson comorbidity index (0, 1, and ≥ 2); BMI (underweight [<18.5 kg/m^2], normal [18.5−23.0 kg/m^2], overweight [23.0–25.0 kg/m^2], obese [≥25.0 kg/m^2], and unknown); blood pressure (systolic blood pressure <140 mmHg and diastolic blood pressure <90 mmHg, systolic blood pressure ≥140 mmHg or diastolic blood pressure ≥90 mmHg, and unknown); fasting blood glucose (<100, ≥100 mg/dL, and unknown); serum total cholesterol (<200, 200–240, ≥240 mg/dL, and unknown); glomerular filtration rate (<60, 60−90, ≥90 mL/min/1.73 m^2, and unknown); smoking status (non- and current smoker, and unknown); alcoholic drinks (<1, 1–2, 3–4, ≥5 days per week, and unknown); aerobic physical activity (sufficient, insufficient, and unknown); previous history of cardiovascular disease, chronic kidney disease, and chronic obstructive pulmonary disease; history of medication use for diabetes mellitus, dyslipidemia, and hypertension; and missing indicators (BMI missing indicator [yes or no], blood pressure missing indicator [yes or no], fasting blood glucose missing indicator [yes or no], serum total cholesterol missing indicator [yes or no], glomerular filtration rate missing indicator [yes or no], smoking status missing indicator [yes or no], alcoholic drinks missing indicator [yes or no], and aerobic physical activity missing indicator [yes or no]).

Table S21. The HR with 95% CI for the long-term sequelae risk of **incident allergic diseases following mild COVID-19 diagnosis** in the propensity score-matched main cohort (South Korea), replication cohort A (Japan), and replication cohort B (UK).

Cohort	Exposure	Incidence rate*	HR (95% CI)	
			Model 1 [a]	Model 2 [b]
**Allergic diseases**				
Main cohort	Non-infected	23.3	1.00 (reference)	1.00 (reference)
Main cohort	Patients with COVID-19	26.8	**1.15 (1.08 to 1.23)**	**1.15 (1.08 to 1.23)**
Replication cohort A	Non-infected	27.4	1.00 (reference)	1.00 (reference)
Replication cohort A	Patients with COVID-19	75.0	**2.64 (2.61 to 2.68)**	**2.56 (2.52 to 2.59)**
Replication cohort B	Non-infected	7.2	1.00 (reference)	1.00 (reference)
Replication cohort B	Patients with COVID-19	8.2	**1.13 (1.05 to 1.21)**	**1.11 (1.04 to 1.19)**
**Asthma**				
Main cohort	Non-infected	0.9	1.00 (reference)	1.00 (reference)
Main cohort	Patients with COVID-19	1.9	**2.17 (1.67 to 2.83)**	**2.18 (1.67 to 2.84)**
Replication cohort A	Non-infected	2.7	1.00 (reference)	1.00 (reference)
Replication cohort A	Patients with COVID-19	7.7	**2.81 (2.68 to 2.94)**	**2.67 (2.55 to 2.80)**
Replication cohort B	Non-infected	3.3	1.00 (reference)	1.00 (reference)
Replication cohort B	Patients with COVID-19	3.8	**1.15 (1.04 to 1.28)**	**1.13 (1.01 to 1.25)**
**Allergic rhinitis**				
Main cohort	Non-infected	16.9	1.00 (reference)	1.00 (reference)
Main cohort	Patients with COVID-19	20.1	**1.19 (1.10 to 1.28)**	**1.19 (1.11 to 1.29)**
Replication cohort A	Non-infected	20.4	1.00 (reference)	1.00 (reference)
Replication cohort A	Patients with COVID-19	62.4	**2.97 (2.92 to 3.02)**	**2.88 (2.83 to 2.93)**
Replication cohort B	Non-infected	1.1	1.00 (reference)	1.00 (reference)
Replication cohort B	Patients with COVID-19	1.4	**1.22 (1.03 to 1.46)**	**1.21 (1.02 to 1.44)**
**Atopic dermatitis**				
Main cohort	Non-infected	2.5	1.00 (reference)	1.00 (reference)
Main cohort	Patients with COVID-19	2.9	1.16 (0.95 to 1.41)	1.16 (0.95 to 1.41)

Replication cohort A	Non-infected	5.2	1.00 (reference)	1.00 (reference)
Replication cohort A	Patients with COVID-19	6.8	**1.24 (1.19 to 1.29)**	**1.21 (1.16 to 1.26)**
Replication cohort B	Non-infected	0.04	1.00 (reference)	1.00 (reference)
Replication cohort B	Patients with COVID-19	0.05	1.18 (0.47 to 2.99)	1.17 (0.46 to 2.98)
**Food allergy**				
Main cohort	Non-infected	3.4	1.00 (reference)	1.00 (reference)
Main cohort	Patients with COVID-19	2.6	**0.76 (0.63 to 0.93)**	**0.76 (0.63 to 0.93)**
Replication cohort A	Non-infected	1.3	1.00 (reference)	1.00 (reference)
Replication cohort A	Patients with COVID-19	2.6	**2.05 (1.90 to 2.20)**	**1.82 (1.69 to 1.96)**
Replication cohort B	Non-infected	3.1	1.00 (reference)	1.00 (reference)
Replication cohort B	Patients with COVID-19	3.4	1.07 (0.96 to 1.20)	1.06 (0.95 to 1.18)

BMI, body mass index; CI, confidence interval; HR, hazard ratio.

* Incidence rate is expressed as per 1,000 person-years.

The data in bold indicate significant differences ($P < 0.05$).

[a] **Model 1**: Adjusted for age (20–39, 40–59, and ≥60 years) and sex.

[b] **Model 2 (main cohort)**: Adjusted for age (20–39, 40–59, and ≥60 years); sex; household income (low income, middle income, and high income); region of residence (urban and rural); Charlson comorbidity index (0, 1, and ≥2); BMI (underweight [<18.5 kg/m^2], normal [18.5–23.0 kg/m^2], overweight [23.0–25.0 kg/m^2], obese [≥25.0 kg/m^2], and unknown); blood pressure (systolic blood pressure <140 mmHg and diastolic blood pressure <90 mmHg, systolic blood pressure ≥140 mmHg or diastolic blood pressure ≥90 mmHg, and unknown); fasting blood glucose (<100, ≥100 mg/dL, and unknown); serum total cholesterol (<200, 200–240, ≥240 mg/dL, and unknown); glomerular filtration rate (<60, 60−90, ≥90 mL/min/1.73 m^2, and unknown); smoking status (non-, ex-, current smoker, and unknown); alcoholic drinks (<1, 1–2, 3–4, ≥5 days per week, and unknown); aerobic physical activity (sufficient, insufficient, and unknown); previous history of cardiovascular disease, chronic kidney disease, and chronic obstructive pulmonary disease; history of medication use for diabetes mellitus, dyslipidemia, and hypertension; and missing indicators (BMI missing indicator [yes or no], blood pressure missing indicator [yes or no], fasting blood glucose missing indicator [yes or no], serum total cholesterol missing indicator [yes or no], glomerular filtration rate missing indicator [yes or no], smoking status missing indicator [yes or no], alcoholic drinks missing indicator [yes or no], and aerobic physical activity missing indicator [yes or no]).

[b] **Model 2 (replication cohort A):** Adjusted for age (20–39, 40–59, and ≥60 years); sex; Charlson comorbidity index (0, 1, and ≥ 2); BMI (underweight [<18.5 kg/m^2], normal [18.5‒23.0 kg/m^2], overweight [23.0–25.0 kg/m^2], obese [≥25.0 kg/m^2], and unknown); blood pressure (systolic blood pressure <140 mmHg and diastolic blood pressure <90 mmHg, systolic blood pressure ≥140 mmHg or diastolic blood pressure ≥90 mmHg, and unknown); fasting blood glucose (<100, ≥100 mg/dL, and unknown); serum total cholesterol (<200, 200–240, ≥240 mg/dL, and unknown); glomerular filtration rate (<60, 60‒90, ≥90 mL/min/1.73 m^2, and unknown); smoking status (non- and current smoker, and unknown); alcoholic drinks (<1, 1–2, 3–4, ≥5 days per week, and unknown); aerobic physical activity (sufficient, insufficient, and unknown); previous history of cardiovascular disease, chronic kidney disease, and chronic obstructive pulmonary disease; history of medication use for diabetes mellitus, dyslipidemia, and hypertension; and missing indicators (BMI missing indicator [yes or no], blood pressure missing indicator [yes or no], fasting blood glucose missing indicator [yes or no], serum total cholesterol missing indicator [yes or no], glomerular filtration rate missing indicator [yes or no], smoking status missing indicator [yes or no], alcoholic drinks missing indicator [yes or no], and aerobic physical activity missing indicator [yes or no]).

[b] **Model 2 (replication cohort B):** Adjusted for age (20–39, 40–59, and ≥ 60 years); sex; household income (<£18,000, £18,000–£30,999, £31,000–£51,999, £52,000–£100,000, >£100,000), and unknown); region of residence (urban and rural); townsend deprivation index (T1 [last deprived], T2, T3 [most deprived], and unknown); race (white, mixed, Asian, black, others, and unknown); Charlson comorbidity index (0, 1, and ≥ 2); BMI (normal [<25.0 kg/m^2], overweight [25.0–30.0 kg/m^2], obese [≥ 30.0 kg/m^2], and unknown); education levels (≤10, 11-12, >12, and unknown); blood pressure (systolic blood pressure < 140 mmHg and diastolic blood pressure < 90 mmHg, systolic blood pressure ≥ 140 mmHg or diastolic blood pressure ≥ 90 mmHg, and unknown); fasting blood glucose (<100, ≥100 mg/dL, and unknown); smoking status (non- and current smoker, and unknown); alcohol consumption (every day, sometimes, rarely days per week, and unknown); aerobic physical activity (low, moderate, high, and unknown); previous history of cardiovascular disease, chronic kidney disease, and chronic obstructive pulmonary disease; history of medication use for diabetes mellitus, dyslipidemia, and hypertension; and missing indicators (household income missing indicator [yes or no], townsend deprivation index missing indicator [yes or no], race missing indicator [yes or no], education levels [yes or no], obesity missing indicator [yes or no], blood pressure missing indicator [yes or no], fasting blood glucose missing indicator [yes or no], serum total cholesterol missing indicator [yes or no], glomerular filtration rate missing indicator [yes or no], smoking status missing indicator [yes or no], alcoholic drinks missing indicator [yes or no], and aerobic physical activity missing indicator [yes or no]).

Table S22. The HR with 95% CI for the long-term sequelae risk of **incident allergic diseases following moderate to severe COVID-19 diagnosis** in the propensity score-matched main cohort (South Korea), replication cohort A (Japan), and replication cohort B (UK).

Cohort	Exposure	Incidence rate*	HR (95% CI)	
			Model 1 [a]	Model 2 [b]
**Allergic diseases**				
Main cohort	Non-infected	27.8	1.00 (reference)	1.00 (reference)
Main cohort	Patients with COVID-19	39.3	**1.41 (1.24 to 1.61)**	**1.42 (1.24 to 1.61)**
Replication cohort A	Non-infected	27.6	1.00 (reference)	1.00 (reference)
Replication cohort A	Patients with COVID-19	81.0	**2.93 (2.68 to 3.20)**	**2.75 (2.49 to 3.03)**
Replication cohort B	Non-infected	5.8	1.00 (reference)	1.00 (reference)
Replication cohort B	Patients with COVID-19	12.2	**2.09 (1.04 to 4.20)**	**1.97 (0.95 to 4.06)**
**Asthma**				
Main cohort	Non-infected	1.5	1.00 (reference)	1.00 (reference)
Main cohort	Patients with COVID-19	4.0	**2.59 (1.66 to 4.03)**	**2.47 (1.58 to 3.85)**
Replication cohort A	Non-infected	3.1	1.00 (reference)	1.00 (reference)
Replication cohort A	Patients with COVID-19	4.4	**1.43 (1.03 to 1.99)**	1.19 (0.83 to 1.71)
Replication cohort B	Non-infected	1.7	1.00 (reference)	1.00 (reference)
Replication cohort B	Patients with COVID-19	5.6	**3.30 (1.06 to 10.25)**	**3.33 (1.05 to 10.60)**
**Allergic rhinitis**				
Main cohort	Non-infected	19.4	1.00 (reference)	1.00 (reference)
Main cohort	Patients with COVID-19	27.7	**1.42 (1.22 to 1.66)**	**1.43 (1.22 to 1.67)**
Replication cohort A	Non-infected	21.0	1.00 (reference)	1.00 (reference)
Replication cohort A	Patients with COVID-19	67.2	**3.19 (2.89 to 3.53)**	**3.09 (2.77 to 3.45)**
Replication cohort B	Non-infected	1.2	1.00 (reference)	1.00 (reference)
Replication cohort B	Patients with COVID-19	0	NA	NA
**Atopic dermatitis**				
Main cohort	Non-infected	2.8	1.00 (reference)	1.00 (reference)
Main cohort	Patients with COVID-19	3.0	1.10 (0.70 to 1.73)	1.11 (0.70 to 1.74)

Replication cohort A	Non-infected	4.2	1.00 (reference)	1.00 (reference)
Replication cohort A	Patients with COVID-19	6.8	**1.64 (1.25 to 2.15)**	**1.57 (1.16 to 2.11)**
Replication cohort B	Non-infected	0	1.00 (reference)	1.00 (reference)
Replication cohort B	Patients with COVID-19	0	NA	NA
**Food allergy**				
Main cohort	Non-infected	4.6	1.00 (reference)	1.00 (reference)
Main cohort	Patients with COVID-19	5.4	1.18 (0.84 to 1.67)	1.18 (0.84 to 1.66)
Replication cohort A	Non-infected	1.5	1.00 (reference)	1.00 (reference)
Replication cohort A	Patients with COVID-19	5.4	**3.77 (2.64 to 5.39)**	**2.53 (1.70 to 3.77)**
Replication cohort B	Non-infected	2.9	1.00 (reference)	1.00 (reference)
Replication cohort B	Patients with COVID-19	7.5	2.47 (0.97 to 6.28)	1.99 (0.74 to 5.39)

BMI, body mass index; CI, confidence interval; HR, hazard ratio.

* Incidence rate is expressed as per 1,000 person-years.

The data in bold indicate significant differences ($P < 0.05$).

[a] **Model 1**: Adjusted for age (20–39, 40–59, and ≥60 years) and sex.

[b] **Model 2 (main cohort)**: Adjusted for age (20–39, 40–59, and ≥60 years); sex; household income (low income, middle income, and high income); region of residence (urban and rural); Charlson comorbidity index (0, 1, and ≥2); BMI (underweight [<18.5 kg/m^2], normal [18.5–23.0 kg/m^2], overweight [23.0–25.0 kg/m^2], obese [≥25.0 kg/m^2], and unknown); blood pressure (systolic blood pressure <140 mmHg and diastolic blood pressure <90 mmHg, systolic blood pressure ≥140 mmHg or diastolic blood pressure ≥90 mmHg, and unknown); fasting blood glucose (<100, ≥100 mg/dL, and unknown); serum total cholesterol (<200, 200–240, ≥240 mg/dL, and unknown); glomerular filtration rate (<60, 60−90, ≥90 mL/min/1.73 m^2, and unknown); smoking status (non-, ex-, current smoker, and unknown); alcoholic drinks (<1, 1–2, 3–4, ≥5 days per week, and unknown); aerobic physical activity (sufficient, insufficient, and unknown); previous history of cardiovascular disease, chronic kidney disease, and chronic obstructive pulmonary disease; history of medication use for diabetes mellitus, dyslipidemia, and hypertension; and missing indicators (BMI missing indicator [yes or no], blood pressure missing indicator [yes or no], fasting blood glucose missing indicator [yes or no], serum total cholesterol missing indicator [yes or no], glomerular filtration rate missing indicator [yes or no], smoking status missing indicator [yes or no], alcoholic drinks missing indicator [yes or no], and aerobic physical activity missing indicator [yes or no]).

[b] **Model 2 (replication cohort A):** Adjusted for age (20–39, 40–59, and ≥60 years); sex; Charlson comorbidity index (0, 1, and ≥ 2); BMI (underweight [<18.5 kg/m^2], normal [18.5–23.0 kg/m^2], overweight [23.0–25.0 kg/m^2], obese [≥25.0 kg/m^2], and unknown); blood pressure (systolic blood pressure <140 mmHg and diastolic blood pressure <90 mmHg, systolic blood pressure ≥140 mmHg or diastolic blood pressure ≥90 mmHg, and unknown); fasting blood glucose (<100, ≥100 mg/dL, and unknown); serum total cholesterol (<200, 200–240, ≥240 mg/dL, and unknown); glomerular filtration rate (<60, 60–90, ≥90 mL/min/1.73 m^2, and unknown); smoking status (non- and current smoker, and unknown); alcoholic drinks (<1, 1–2, 3–4, ≥5 days per week, and unknown); aerobic physical activity (sufficient, insufficient, and unknown); previous history of cardiovascular disease, chronic kidney disease, and chronic obstructive pulmonary disease; history of medication use for diabetes mellitus, dyslipidemia, and hypertension; and missing indicators (BMI missing indicator [yes or no], blood pressure missing indicator [yes or no], fasting blood glucose missing indicator [yes or no], serum total cholesterol missing indicator [yes or no], glomerular filtration rate missing indicator [yes or no], smoking status missing indicator [yes or no], alcoholic drinks missing indicator [yes or no], and aerobic physical activity missing indicator [yes or no]).

[b] **Model 2 (replication cohort B):** Adjusted for age (20–39, 40–59, and ≥ 60 years); sex; household income (<£18,000, £18,000–£30,999, £31,000–£51,999, £52,000–£100,000, >£100,000), and unknown); region of residence (urban and rural); townsend deprivation index (T1 [last deprived], T2, T3 [most deprived], and unknown); race (white, mixed, Asian, black, others, and unknown); Charlson comorbidity index (0, 1, and ≥ 2); BMI (normal [<25.0 kg/m^2], overweight [25.0–30.0 kg/m^2], obese [≥ 30.0 kg/m^2], and unknown); education levels (≤10, 11-12, >12, and unknown); blood pressure (systolic blood pressure < 140 mmHg and diastolic blood pressure < 90 mmHg, systolic blood pressure ≥ 140 mmHg or diastolic blood pressure ≥ 90 mmHg, and unknown); fasting blood glucose (<100, ≥100 mg/dL, and unknown); smoking status (non- and current smoker, and unknown); alcohol consumption (every day, sometimes, rarely days per week, and unknown); aerobic physical activity (low, moderate, high, and unknown); previous history of cardiovascular disease, chronic kidney disease, and chronic obstructive pulmonary disease; history of medication use for diabetes mellitus, dyslipidemia, and hypertension; and missing indicators (household income missing indicator [yes or no], townsend deprivation index missing indicator [yes or no], race missing indicator [yes or no], education levels [yes or no], obesity missing indicator [yes or no], blood pressure missing indicator [yes or no], fasting blood glucose missing indicator [yes or no], serum total cholesterol missing indicator [yes or no], glomerular filtration rate missing indicator [yes or no], smoking status missing indicator [yes or no], alcoholic drinks missing indicator [yes or no], and aerobic physical activity missing indicator [yes or no]).

Table S23. The HR with 95% CI for the long-term sequelae risk of incident allergic diseases following COVID-19 diagnosis of patients in the propensity score-matched cohorts in main cohort (South Korea), replication cohort A (Japan), and replication cohort B (UK), presenting the *P* value with a two-sided test from Table 2.

Cohort	Model 1 [a]		Model 2 [b]	
	HR (95% CI)	P value	HR (95% CI)	P value
**Allergic diseases**				
Main cohort	**1.20 (1.13 to 1.27)**	**<0.001**	**1.20 (1.13 to 1.27)**	**<0.001**
Replication cohort A	**2.65 (2.61 to 2.69)**	**<0.001**	**2.56 (2.52 to 2.59)**	**<0.001**
Replication cohort B	**1.14 (1.06 to 1.22)**	**<0.001**	**1.12 (1.04 to 1.20)**	**0.002**
**Asthma**				
Main cohort	**2.27 (1.81 to 2.85)**	**<0.001**	**2.25 (1.80 to 2.83)**	**<0.001**
Replication cohort A	**2.77 (2.65 to 2.90)**	**<0.001**	**2.63 (2.51 to 2.75)**	**<0.001**
Replication cohort B	**1.16 (1.05 to 1.29)**	**0.005**	**1.14 (1.03 to 1.27)**	**0.014**
**Allergic rhinitis**				
Main cohort	**1.23 (1.15 to 1.32)**	**<0.001**	**1.23 (1.15 to 1.32)**	**<0.001**
Replication cohort A	**2.98 (2.93 to 3.03)**	**<0.001**	**2.88 (2.83 to 2.93)**	**<0.001**
Replication cohort B	**1.21 (1.02 to 1.44)**	**0.030**	**1.20 (1.01 to 1.43)**	**0.043**
**Atopic dermatitis**				
Main cohort	1.15 (0.96 to 1.37)	0.132	1.15 (0.96 to 1.37)	0.138
Replication cohort A	**1.25 (1.20 to 1.30)**	**<0.001**	**1.21 (1.16 to 1.26)**	**<0.001**
Replication cohort B	1.18 (0.46 to 2.99)	0.730	1.19 (0.47 to 3.01)	0.721
**Food allergy**				
Main cohort	0.85 (0.71 to 1.01)	0.056	0.85 (0.71 to 1.00)	0.055
Replication cohort A	**2.10 (1.95 to 2.25)**	**<0.001**	**1.84 (1.71 to 1.98)**	**<0.001**
Replication cohort B	1.09 (0.97 to 1.21)	0.137	1.07 (0.96 to 1.20)	0.218

BMI, body mass index; CI, confidence interval; HR, hazard ratio.

* Incidence rate is expressed as per 1,000 person-years.

The data in bold indicate significant differences (*P* < 0.05).

[a] **Model 1**: Adjusted for age (20–39, 40–59, and ≥60 years) and sex.

[b] **Model 2 (main cohort)**: Adjusted for age (20–39, 40–59, and ≥60 years); sex; household income (low income, middle income, and high income); region of residence (urban and rural); Charlson comorbidity index (0, 1, and ≥2); BMI (underweight [<18.5 kg/m^2], normal [18.5–23.0 kg/m^2], overweight [23.0–25.0 kg/m^2], obese [≥25.0 kg/m^2], and unknown); blood pressure (systolic blood pressure <140 mmHg and diastolic blood pressure <90 mmHg, systolic blood pressure ≥140 mmHg or diastolic blood pressure ≥90 mmHg, and unknown); fasting blood glucose (<100, ≥100 mg/dL, and unknown); serum total cholesterol (<200, 200–240, ≥240 mg/dL, and unknown); glomerular filtration rate (<60, 60−90, ≥90 mL/min/1.73 m^2, and unknown); smoking status (non-, ex-, current smoker, and unknown); alcoholic drinks (<1, 1–2, 3−4, ≥5 days per week, and unknown); aerobic physical activity (sufficient, insufficient, and unknown); previous history of cardiovascular disease, chronic kidney disease, and chronic obstructive pulmonary disease; history of medication use for diabetes mellitus, dyslipidemia, and hypertension; and missing indicators (BMI missing indicator [yes or no], blood pressure missing indicator [yes or no], fasting blood glucose missing indicator [yes or no], serum total cholesterol missing indicator [yes or no], glomerular filtration rate missing indicator [yes or no], smoking status missing indicator [yes or no], alcoholic drinks missing indicator [yes or no], and aerobic physical activity missing indicator [yes or no]).

[b] **Model 2 (replication cohort A):** Adjusted for age (20–39, 40–59, and ≥60 years); sex; Charlson comorbidity index (0, 1, and ≥ 2); BMI (underweight [<18.5 kg/m^2], normal [18.5−23.0 kg/m^2], overweight [23.0–25.0 kg/m^2], obese [≥25.0 kg/m^2], and unknown); blood pressure (systolic blood pressure <140 mmHg and diastolic blood pressure <90 mmHg, systolic blood pressure ≥140 mmHg or diastolic blood pressure ≥90 mmHg, and unknown); fasting blood glucose (<100, ≥100 mg/dL, and unknown); serum total cholesterol (<200, 200–240, ≥240 mg/dL, and unknown); glomerular filtration rate (<60, 60−90, ≥90 mL/min/1.73 m^2, and unknown); smoking status (non- and current smoker, and unknown); alcoholic drinks (<1, 1–2, 3–4, ≥5 days per week, and unknown); aerobic physical activity (sufficient, insufficient, and unknown); previous history of cardiovascular disease, chronic kidney disease, and chronic obstructive pulmonary disease; history of medication use for diabetes mellitus, dyslipidemia, and hypertension; and missing indicators (BMI missing indicator [yes or no], blood pressure missing indicator [yes or no], fasting blood glucose missing indicator [yes or no], serum total cholesterol missing indicator [yes or no], glomerular filtration rate missing indicator [yes or no], smoking status missing indicator [yes or no], alcoholic drinks missing indicator [yes or no], and aerobic physical activity missing indicator [yes or no]).

[b] **Model 2 (replication cohort B):** Adjusted for age (20–39, 40–59, and ≥ 60 years); sex; household income (<£18,000, £18,000–£30,999, £31,000–£51,999, £52,000–£100,000, >£100,000), and unknown); region of residence (urban and rural); townsend deprivation index (T1 [last deprived], T2, T3 [most deprived], and unknown); ethnicity (white, mixed, Asian, black, others, and unknown); Charlson comorbidity index (0, 1, and ≥ 2); BMI (normal [<25.0 kg/m^2], overweight [25.0–30.0 kg/m^2], obese [≥ 30.0 kg/m^2], and unknown); education levels (≤10, 11-12, >12, and unknown); blood pressure (systolic blood pressure < 140 mmHg and diastolic blood pressure < 90 mmHg, systolic blood pressure ≥ 140 mmHg or diastolic blood pressure ≥ 90 mmHg, and unknown); fasting blood glucose (<100, ≥100 mg/dL, and unknown); smoking status (non- and current smoker, and unknown); alcohol consumption (every day, sometimes, rarely days per week, and unknown); aerobic physical activity (low, moderate, high, and unknown); previous history of cardiovascular disease, chronic kidney disease, and chronic

obstructive pulmonary disease; history of medication use for diabetes mellitus, dyslipidemia, and hypertension; and missing indicators (household income missing indicator [yes or no], townsend deprivation index missing indicator [yes or no], ethnicity missing indicator [yes or no], education levels [yes or no], obesity missing indicator [yes or no], blood pressure missing indicator [yes or no], fasting blood glucose missing indicator [yes or no], serum total cholesterol missing indicator [yes or no], glomerular filtration rate missing indicator [yes or no], smoking status missing indicator [yes or no], alcoholic drinks missing indicator [yes or no], and aerobic physical activity missing indicator [yes or no]).

Table S24. Time attenuation effect on the development of allergic diseases after SARS-CoV-2 infection (model 2; adjusted HR with 95% CI), presenting the *P* value with a two-sided test from Table 3.

	HR (95% CI)			
	Main cohort*	P value	Replication cohort‡	P value
Allergic diseases				
Within 6 months	**1.42 (1.29 to 1.56)**	**<0.001**	**3.30 (3.24 to 3.36)**	**<0.001**
Between 6 to 12 months	**1.14 (1.01 to 1.29)**	**0.036**	**1.77 (1.70 to 1.84)**	**<0.001**
After 12 months	1.00 (0.91 to 1.11)	0.981	**1.61 (1.56 to 1.67)**	**<0.001**

CI, confidence interval; HR, hazard ratio; SARS-CoV-2, severe acute respiratory syndrome coronavirus.

The data in bold indicate significant differences ($P < 0.05$).

Model 2 (main cohort): Adjusted for age (20–39, 40–59, and ≥60 years); sex; household income (low income, middle income, and high income); region of residence (urban and rural); Charlson comorbidity index (0, 1, and ≥2); BMI (underweight [<18.5 kg/m^2], normal [18.5–23.0 kg/m^2], overweight [23.0–25.0 kg/m^2], obese [≥25.0 kg/m^2], and unknown); blood pressure (systolic blood pressure <140 mmHg and diastolic blood pressure <90 mmHg, systolic blood pressure ≥140 mmHg or diastolic blood pressure ≥90 mmHg, and unknown); fasting blood glucose (<100, ≥100 mg/dL, and unknown); serum total cholesterol (<200, 200–240, ≥240 mg/dL, and unknown); glomerular filtration rate (<60, 60−90, ≥90 mL/min/1.73 m^2, and unknown); smoking status (non-, ex-, current smoker, and unknown); alcoholic drinks (<1, 1–2, 3–4, ≥5 days per week, and unknown); aerobic physical activity (sufficient, insufficient, and unknown); previous history of cardiovascular disease, chronic kidney disease, and chronic obstructive pulmonary disease; history of medication use for diabetes mellitus, dyslipidemia, and hypertension; and missing indicators (BMI missing indicator [yes or no], blood pressure missing indicator [yes or no], fasting blood glucose missing indicator [yes or no], serum total cholesterol missing indicator [yes or no], glomerular filtration rate missing indicator [yes or no], smoking status missing indicator [yes or no], alcoholic drinks missing indicator [yes or no], and aerobic physical activity missing indicator [yes or no]).

Model 2 (replication cohort A): Adjusted for age (20–39, 40–59, and ≥60 years); sex; Charlson comorbidity index (0, 1, and ≥ 2); BMI (underweight [<18.5 kg/m^2], normal [18.5–23.0 kg/m^2], overweight [23.0–25.0 kg/m^2], obese [≥25.0 kg/m^2], and unknown); blood pressure (systolic blood pressure <140 mmHg and diastolic blood pressure <90 mmHg, systolic blood pressure ≥140 mmHg or diastolic blood pressure ≥90 mmHg, and unknown); fasting blood glucose (<100, ≥100 mg/dL, and unknown); serum total cholesterol (<200, 200–240, ≥240 mg/dL, and unknown); glomerular filtration rate (<60, 60–90, ≥90 mL/min/1.73 m^2, and unknown); smoking status (non- and current smoker, and unknown); alcoholic drinks (<1, 1–2, 3–4, ≥5 days per week, and unknown); aerobic physical activity (sufficient, insufficient, and unknown); previous history of cardiovascular disease, chronic kidney disease, and chronic obstructive pulmonary disease; history of medication use for diabetes mellitus, dyslipidemia, and hypertension; and missing indicators (BMI missing indicator [yes or no], blood pressure missing indicator [yes or no], fasting blood glucose missing indicator [yes or no], serum total cholesterol missing indicator [yes or no], glomerular filtration rate missing indicator [yes or no], smoking status missing indicator [yes or no], alcoholic drinks missing indicator [yes or no], and aerobic physical activity missing indicator [yes or no]).

Table 25. Propensity-score-matched subgroup analysis of HR (95% CI) of allergic diseases following COVID-19 diagnosis stratified by COVID-19 severity, SARS-CoV-2 strain type, and number of vaccinations in main cohort (South Korea), presenting the *P* value with a two-sided test from Table 4.

	HR (95% CI)			
Group	Model 1 [a]	P value	Model 2 [b]	P value
COVID-19 severity				
Mild COVID-19	**1.13 (1.06 to 1.20)**	**<0.001**	**1.14 (1.07 to 1.21)**	**<0.001**
Moderate to severe COVID-19	**1.55 (1.38 to 1.74)**	**<0.001**	**1.48 (1.31 to 1.66)**	**<0.001**
SARS-CoV-2 vaccination dosage				
COVID-19 without SARS-CoV-2 vaccination	**1.24 (1.16 to 1.32)**	**<0.001**	**1.24 (1.16 to 1.32)**	**<0.001**
COVID-19 after SARS-CoV-2 vaccination received once	**1.43 (1.22 to 1.69)**	**<0.001**	**1.44 (1.22 to 1.69)**	**<0.001**
COVID-19 after SARS-CoV-2 vaccination received twice or more	**0.81 (0.68 to 0.96)**	**0.014**	**0.81 (0.68 to 0.96)**	**0.014**
Strain type (original)				
Original COVID-19	**1.20 (1.12 to 1.29)**	**<0.001**	**1.20 (1.12 to 1.29)**	**<0.001**
Strain type (delta)				
Delta COVID-19	**1.18 (1.05 to 1.32)**	**<0.001**	**1.18 (1.05 to 1.32)**	**<0.001**

HR, hazard ratio; CI, confidence interval; SARS-CoV-2, severe acute respiratory syndrome coronavirus.

The data in bold indicate significant differences ($P < 0.05$).

[a] **Model 1**: Adjusted for age (20–39, 40–59, and ≥60 years) and sex.

[b] **Model 2 (main cohort)**: Adjusted for age (20–39, 40–59, and ≥60 years); sex; household income (low income, middle income, and high income); region of

residence (urban and rural); Charlson comorbidity index (0, 1, and ≥2); BMI (underweight [<18.5 kg/m^2], normal [18.5–23.0 kg/m^2], overweight [23.0–25.0 kg/m^2], obese [≥25.0 kg/m^2], and unknown); blood pressure (systolic blood pressure <140 mmHg and diastolic blood pressure <90 mmHg, systolic blood pressure ≥140 mmHg or diastolic blood pressure ≥90 mmHg, and unknown); fasting blood glucose (<100, ≥100 mg/dL, and unknown); serum total cholesterol (<200, 200–240, ≥240 mg/dL, and unknown); glomerular filtration rate (<60, 60−90, ≥90 mL/min/1.73 m^2, and unknown); smoking status (non-, ex-, current smoker, and unknown); alcoholic drinks (<1, 1–2, 3–4, ≥5 days per week, and unknown); aerobic physical activity (sufficient, insufficient, and unknown); previous history of cardiovascular disease, chronic kidney disease, and chronic obstructive pulmonary disease; history of medication use for diabetes mellitus, dyslipidemia, and hypertension; and missing indicators (BMI missing indicator [yes or no], blood pressure missing indicator [yes or no], fasting blood glucose missing indicator [yes or no], serum total cholesterol missing indicator [yes or no], glomerular filtration rate missing indicator [yes or no], smoking status missing indicator [yes or no], alcoholic drinks missing indicator [yes or no], and aerobic physical activity missing indicator [yes or no]).

‡ Comparators defined only 1:5 matched comparators in each patient group at the same index date to reduce immortal bias.

Table S26. The HR with 95% CI for the long-term sequelae risk of incident allergic disease in **negative control analysis** using non-COVID-19 disease (**tympanic membrane perforation**) in the propensity score-matched main cohort (South Korea), replication cohort A (Japan), and replication cohort B (UK).

Cohort	Exposure	Incidence rate*	HR (95% CI)	
			Model 1 [a]	Model 2 [b]
Main cohort	None	0.5	1.00 (reference)	1.00 (reference)
Main cohort	Patients with COVID-19	0.4	0.85 (0.60 to 1.20)	0.85 (0.60 to 1.20)
Replication cohort A	None	0.0	1.00 (reference)	1.00 (reference)
Replication cohort A	Patients with COVID-19	0.1	1.09 (0.68 to 1.74)	0.81 (0.39 to 1.68)
Replication cohort B	None	0.39	1.00 (reference)	1.00 (reference)
Replication cohort B	Patients with COVID-19	0.38	0.97 (0.72 to 1.30)	0.98 (0.73 to 1.32)

BMI, body mass index; CI, confidence interval; HR, hazard ratio.

* Incidence rate is expressed as per 1,000 person-years.

[a] **Model 1**: Adjusted for age (20–39, 40–59, and ≥60 years) and sex.

[b] **Model 2 (main cohort)**: Adjusted for age (20–39, 40–59, and ≥60 years); sex; household income (low income, middle income, and high income); region of residence (urban and rural); Charlson comorbidity index (0, 1, and ≥2); BMI (underweight [<18.5 kg/m^2], normal [18.5–23.0 kg/m^2], overweight [23.0–25.0 kg/m^2], obese [≥25.0 kg/m^2], and unknown); blood pressure (systolic blood pressure <140 mmHg and diastolic blood pressure <90 mmHg, systolic blood pressure ≥140 mmHg or diastolic blood pressure ≥90 mmHg, and unknown); fasting blood glucose (<100, ≥100 mg/dL, and unknown); serum total cholesterol (<200, 200–240, ≥240 mg/dL, and unknown); glomerular filtration rate (<60, 60−90, ≥90 mL/min/1.73 m^2, and unknown); smoking status (non-, ex-, current smoker, and unknown); alcoholic drinks (<1, 1–2, 3−4, ≥5 days per week, and unknown); aerobic physical activity (sufficient, insufficient, and unknown); previous history of cardiovascular disease, chronic kidney disease, and chronic obstructive pulmonary disease; history of medication use for diabetes mellitus, dyslipidemia, and hypertension; and missing indicators (BMI missing indicator [yes or no], blood pressure missing indicator [yes or no], fasting blood glucose missing indicator [yes or no], serum total cholesterol missing indicator [yes or no], glomerular filtration rate missing indicator [yes or no], smoking status missing indicator [yes or no], alcoholic drinks missing indicator [yes or no], and aerobic physical activity missing indicator [yes or no]).

[b] **Model 2 (replication cohort A):** Adjusted for age (20–39, 40–59, and ≥60 years); sex; Charlson comorbidity index (0, 1, and ≥ 2); BMI (underweight [<18.5 kg/m^2], normal [18.5–23.0 kg/m^2], overweight [23.0–25.0 kg/m^2], obese [≥25.0 kg/m^2], and unknown); blood pressure (systolic blood pressure <140 mmHg and diastolic blood pressure <90 mmHg, systolic blood pressure ≥140 mmHg or diastolic blood pressure ≥90 mmHg, and unknown); fasting blood glucose (<100, ≥100 mg/dL, and unknown); serum total cholesterol (<200, 200–240, ≥240 mg/dL, and unknown); glomerular filtration rate (<60, 60–90, ≥90 mL/min/1.73 m^2, and unknown); smoking status (non- and current smoker, and unknown); alcoholic drinks (<1, 1–2, 3–4, ≥5 days per week, and unknown); aerobic physical activity (sufficient, insufficient, and unknown); previous history of cardiovascular disease, chronic kidney disease, and chronic obstructive pulmonary disease; history of medication use for diabetes mellitus, dyslipidemia, and hypertension; and missing indicators (BMI missing indicator [yes or no], blood pressure missing indicator [yes or no], fasting blood glucose missing indicator [yes or no], serum total cholesterol missing indicator [yes or no], glomerular filtration rate missing indicator [yes or no], smoking status missing indicator [yes or no], alcoholic drinks missing indicator [yes or no], and aerobic physical activity missing indicator [yes or no]).

[b] **Model 2 (replication cohort B):** Adjusted for age (20–39, 40–59, and ≥ 60 years); sex; household income (<£18,000, £18,000–£30,999, £31,000–£51,999, £52,000–£100,000, >£100,000), and unknown); region of residence (urban and rural); townsend deprivation index (T1 [last deprived], T2, T3 [most deprived], and unknown); race (white, mixed, Asian, black, others, and unknown); Charlson comorbidity index (0, 1, and ≥ 2); BMI (normal [<25.0 kg/m^2], overweight [25.0–30.0 kg/m^2], obese [≥ 30.0 kg/m^2], and unknown); education levels (≤10, 11-12, >12, and unknown); blood pressure (systolic blood pressure < 140 mmHg and diastolic blood pressure < 90 mmHg, systolic blood pressure ≥ 140 mmHg or diastolic blood pressure ≥ 90 mmHg, and unknown); fasting blood glucose (<100, ≥100 mg/dL, and unknown); smoking status (non- and current smoker, and unknown); alcohol consumption (every day, sometimes, rarely days per week, and unknown); aerobic physical activity (low, moderate, high, and unknown); previous history of cardiovascular disease, chronic kidney disease, and chronic obstructive pulmonary disease; history of medication use for diabetes mellitus, dyslipidemia, and hypertension; and missing indicators (household income missing indicator [yes or no], townsend deprivation index missing indicator [yes or no], race missing indicator [yes or no], education levels [yes or no], obesity missing indicator [yes or no], blood pressure missing indicator [yes or no], fasting blood glucose missing indicator [yes or no], serum total cholesterol missing indicator [yes or no], glomerular filtration rate missing indicator [yes or no], smoking status missing indicator [yes or no], alcoholic drinks missing indicator [yes or no], and aerobic physical activity missing indicator [yes or no]).

Table S27. The HR with 95% CI for the long-term sequelae risk of **incident asthma without dyspnea** following COVID-19 diagnosis of patients in the propensity score-matched main cohort (South Korea), replication cohort A (Japan), and replication cohort B (UK).

Cohort	Exposure	n (%)	Incidence rate*	HR (95% CI)	
				Model 1 [a]	Model 2 [b]
**Allergic diseases**					
Main cohort	None	675,760 (82.48)	23.7	1.00 (reference)	1.00 (reference)
Main cohort	Patients with COVID-19	143,563 (17.52)	27.6	**1.16 (1.10 to 1.23)**	**1.16 (1.10 to 1.24)**
Replication cohort A	None	1,989,799 (78.94)	27.3	1.00 (reference)	1.00 (reference)
Replication cohort A	Patients with COVID-19	530,924 (21.06)	74.5	**2.64 (2.60 to 2.68)**	**2.54 (2.51 to 2.58)**
Replication cohort B	None	248,936 (79.40)	7.2	1.00 (reference)	1.00 (reference)
Replication cohort B	Patients with COVID-19	76,886 (23.60)	8.2	**1.13 (1.06 to 1.22)**	**1.12 (1.04 to 1.20)**
**Asthma**					
Main cohort	None	675,760 (82.48)	0.8	1.00 (reference)	1.00 (reference)
Main cohort	Patients with COVID-19	143,563 (17.52)	1.8	**2.13 (1.65 to 2.75)**	**2.12 (1.65 to 2.74)**
Replication cohort A	None	1,989,799 (78.94)	2.6	1.00 (reference)	1.00 (reference)
Replication cohort A	Patients with COVID-19	530,924 (21.06)	7.5	**2.74 (2.62 to 2.87)**	**2.61 (2.49 to 2.74)**
Replication cohort B	None	248,936 (79.40)	2.7	1.00 (reference)	1.00 (reference)
Replication cohort B	Patients with COVID-19	76,886 (23.60)	3.7	**1.15 (1.04 to 1.28)**	**1.13 (1.02 to 1.26)**

BMI, body mass index; CI, confidence interval; HR, hazard ratio.

* Incidence rate is expressed as per 1,000 person-years.

The data in bold indicate significant differences (P < 0.05).

[a] **Model 1**: Adjusted for age (20–39, 40–59, and ≥60 years) and sex.

[b] **Model 2 (main cohort)**: Adjusted for age (20–39, 40–59, and ≥60 years); sex; household income (low income, middle income, and high income); region of residence (urban and rural); Charlson comorbidity index (0, 1, and ≥2); BMI (underweight [<18.5 kg/m^2], normal [18.5–23.0 kg/m^2], overweight [23.0–25.0 kg/m^2], obese [≥25.0 kg/m^2], and unknown); blood pressure (systolic blood pressure <140 mmHg and diastolic blood pressure <90 mmHg, systolic blood pressure ≥140 mmHg or diastolic blood pressure ≥90 mmHg, and unknown); fasting blood glucose (<100, ≥100 mg/dL, and unknown); serum total cholesterol (<200,

200–240, ≥240 mg/dL, and unknown); glomerular filtration rate (<60, 60−90, ≥90 mL/min/1.73 m^2, and unknown); smoking status (non-, ex-, current smoker, and unknown); alcoholic drinks (<1, 1–2, 3–4, ≥5 days per week, and unknown); aerobic physical activity (sufficient, insufficient, and unknown); previous history of cardiovascular disease, chronic kidney disease, and chronic obstructive pulmonary disease; history of medication use for diabetes mellitus, dyslipidemia, and hypertension; and missing indicators (BMI missing indicator [yes or no], blood pressure missing indicator [yes or no], fasting blood glucose missing indicator [yes or no], serum total cholesterol missing indicator [yes or no], glomerular filtration rate missing indicator [yes or no], smoking status missing indicator [yes or no], alcoholic drinks missing indicator [yes or no], and aerobic physical activity missing indicator [yes or no]).

[b] **Model 2 (replication cohort A):** Adjusted for age (20–39, 40–59, and ≥60 years); sex; Charlson comorbidity index (0, 1, and ≥ 2); BMI (underweight [<18.5 kg/m^2], normal [18.5−23.0 kg/m^2], overweight [23.0–25.0 kg/m^2], obese [≥25.0 kg/m^2], and unknown); blood pressure (systolic blood pressure <140 mmHg and diastolic blood pressure <90 mmHg, systolic blood pressure ≥140 mmHg or diastolic blood pressure ≥90 mmHg, and unknown); fasting blood glucose (<100, ≥100 mg/dL, and unknown); serum total cholesterol (<200, 200–240, ≥240 mg/dL, and unknown); glomerular filtration rate (<60, 60−90, ≥90 mL/min/1.73 m^2, and unknown); smoking status (non- and current smoker, and unknown); alcoholic drinks (<1, 1–2, 3–4, ≥5 days per week, and unknown); aerobic physical activity (sufficient, insufficient, and unknown); previous history of cardiovascular disease, chronic kidney disease, and chronic obstructive pulmonary disease; history of medication use for diabetes mellitus, dyslipidemia, and hypertension; and missing indicators (BMI missing indicator [yes or no], blood pressure missing indicator [yes or no], fasting blood glucose missing indicator [yes or no], serum total cholesterol missing indicator [yes or no], glomerular filtration rate missing indicator [yes or no], smoking status missing indicator [yes or no], alcoholic drinks missing indicator [yes or no], and aerobic physical activity missing indicator [yes or no]).

[b] **Model 2 (replication cohort B):** Adjusted for age (20–39, 40–59, and ≥ 60 years); sex; household income (<£18,000, £18,000–£30,999, £31,000–£51,999, £52,000–£100,000, >£100,000), and unknown); region of residence (urban and rural); townsend deprivation index (T1 [last deprived], T2, T3 [most deprived], and unknown); race (white, mixed, Asian, black, others, and unknown); Charlson comorbidity index (0, 1, and ≥ 2); BMI (normal [<25.0 kg/m^2], overweight [25.0–30.0 kg/m^2], obese [≥ 30.0 kg/m^2], and unknown); education levels (≤10, 11-12, >12, and unknown); blood pressure (systolic blood pressure < 140 mmHg and diastolic blood pressure < 90 mmHg, systolic blood pressure ≥ 140 mmHg or diastolic blood pressure ≥ 90 mmHg, and unknown); fasting blood glucose (<100, ≥100 mg/dL, and unknown); smoking status (non- and current smoker, and unknown); alcohol consumption (every day, sometimes, rarely days per week, and unknown); aerobic physical activity (low, moderate, high, and unknown); previous history of cardiovascular disease, chronic kidney disease, and chronic obstructive pulmonary disease; history of medication use for diabetes mellitus, dyslipidemia, and hypertension; and missing indicators (household income missing indicator [yes or no], townsend deprivation index missing indicator [yes or no], race missing indicator [yes or no], education levels [yes or no], obesity missing indicator [yes or no], blood pressure missing indicator [yes or no], fasting blood glucose missing indicator [yes or no], serum total cholesterol missing indicator [yes or no], glomerular filtration rate missing indicator [yes or no], smoking status missing indicator [yes or no], alcoholic drinks missing indicator [yes or no], and aerobic physical activity missing indicator [yes or no]).

Table S28. The HR with 95% CI for the long-term sequelae risk of **incident asthma with EDHO** following COVID-19 diagnosis of patients in the propensity score-matched main cohort (South Korea).

Exposure	n (%)	Incidence rate*	HR (95% CI)	
			Model 1 [a]	Model 2 [b]
**Allergic diseases**				
None	688,340 (82.32)	24.0	1.00 (reference)	1.00 (reference)
Patients with COVID-19	147,824 (17.68)	28.7	**1.20 (1.13 to 1.27)**	**1.20 (1.13 to 1.27)**
**Asthma**				
None	688,340 (82.32)	0.4	1.00 (reference)	1.00 (reference)
Patients with COVID-19	147,824 (17.68)	1.2	**2.86 (2.08 to 3.94)**	**2.74 (1.99 to 3.78)**

BMI, body mass index; CI, confidence interval; EDHO, history of emergency department visits or hospitalizations; HR, hazard ratio.

* Incidence rate is expressed as per 1,000 person-years.

The data in bold indicate significant differences ($P < 0.05$).

[a] **Model 1**: Adjusted for age (20–39, 40–59, and ≥60 years) and sex.

[b] **Model 2 (South Korea)**: Adjusted for age (20–39, 40–59, and ≥60 years); sex; household income (low income, middle income, and high income); region of residence (urban and rural); Charlson comorbidity index (0, 1, and ≥2); BMI (underweight [<18.5 kg/m^2], normal [18.5–23.0 kg/m^2], overweight [23.0–25.0 kg/m^2], obese [≥25.0 kg/m^2], and unknown); blood pressure (systolic blood pressure <140 mmHg and diastolic blood pressure <90 mmHg, systolic blood pressure ≥140 mmHg or diastolic blood pressure ≥90 mmHg, and unknown); fasting blood glucose (<100, ≥100 mg/dL, and unknown); serum total cholesterol (<200, 200–240, ≥240 mg/dL, and unknown); glomerular filtration rate (<60, 60−90, ≥90 mL/min/1.73 m^2, and unknown); smoking status (non-, ex-, current smoker, and unknown); alcoholic drinks (<1, 1–2, 3–4, ≥5 days per week, and unknown); aerobic physical activity (sufficient, insufficient, and unknown); previous history of cardiovascular disease, chronic kidney disease, and chronic obstructive pulmonary disease; history of medication use for diabetes mellitus, dyslipidemia, and hypertension; and missing indicators (BMI missing indicator [yes or no], blood pressure missing indicator [yes or no], fasting blood glucose missing indicator [yes or no], serum total cholesterol missing indicator [yes or no], glomerular filtration rate missing indicator [yes or no], smoking status missing indicator [yes or no], alcoholic drinks missing indicator [yes or no], and aerobic physical activity missing indicator [yes or no]).

Table S29. The HR with 95% CI for the long-term sequelae risk of **incident asthma phenotype (allergic asthma and non-allergic asthma)** following COVID-19 diagnosis in the propensity score-matched main cohort (South Korea), replication cohort A (Japan), and replication cohort B (UK).

Cohort	Exposure	Incidence rate*	HR (95% CI)	
			Model 1 [a]	Model 2 [b]
**Allergic asthma**				
Main cohort	Non-infected	0.3	1.00 (reference)	1.00 (reference)
Main cohort	Patients with COVID-19	0.5	**1.95 (1.24 to 3.04)**	**1.93 (1.23 to 3.01)**
Replication cohort A	Non-infected	1.3	1.00 (reference)	1.00 (reference)
Replication cohort A	Patients with COVID-19	3.8	**2.78 (2.61 to 2.97)**	**2.66 (2.50 to 2.84)**
Replication cohort B	Non-infected	0.2	1.00 (reference)	1.00 (reference)
Replication cohort B	Patients with COVID-19	0.2	1.00 (0.64 to 1.55)	0.97 (0.62 to 1.50)
**Non-allergic asthma**				
Main cohort	Non-infected	0.7	1.00 (reference)	1.00 (reference)
Main cohort	Patients with COVID-19	1.7	**2.40 (1.84 to 3.13)**	**2.38 (1.83 to 3.11)**
Replication cohort A	Non-infected	1.4	1.00 (reference)	1.00 (reference)
Replication cohort A	Patients with COVID-19	3.8	**2.76 (2.59 to 2.94)**	**2.60 (2.44 to 2.78)**
Replication cohort B	Non-infected	3.0	1.00 (reference)	1.00 (reference)
Replication cohort B	Patients with COVID-19	3.6	**1.17 (1.05 to 1.30)**	**1.15 (1.04 to 1.28)**

BMI, body mass index; CI, confidence interval; HR, hazard ratio.

* Incidence rate is expressed as per 1,000 person-years.

The data in bold indicate significant differences ($P < 0.05$).

[a] **Model 1**: Adjusted for age (20–39, 40–59, and ≥60 years) and sex.

[b] **Model 2 (main cohort)**: Adjusted for age (20–39, 40–59, and ≥60 years); sex; household income (low income, middle income, and high income); region of

residence (urban and rural); Charlson comorbidity index (0, 1, and ≥2); BMI (underweight [<18.5 kg/m^2], normal [18.5–23.0 kg/m^2], overweight [23.0–25.0 kg/m^2], obese [≥25.0 kg/m^2], and unknown); blood pressure (systolic blood pressure <140 mmHg and diastolic blood pressure <90 mmHg, systolic blood pressure ≥140 mmHg or diastolic blood pressure ≥90 mmHg, and unknown); fasting blood glucose (<100, ≥100 mg/dL, and unknown); serum total cholesterol (<200, 200–240, ≥240 mg/dL, and unknown); glomerular filtration rate (<60, 60−90, ≥90 mL/min/1.73 m^2, and unknown); smoking status (non-, ex-, current smoker, and unknown); alcoholic drinks (<1, 1–2, 3–4, ≥5 days per week, and unknown); aerobic physical activity (sufficient, insufficient, and unknown); previous history of cardiovascular disease, chronic kidney disease, and chronic obstructive pulmonary disease; history of medication use for diabetes mellitus, dyslipidemia, and hypertension; and missing indicators (BMI missing indicator [yes or no], blood pressure missing indicator [yes or no], fasting blood glucose missing indicator [yes or no], serum total cholesterol missing indicator [yes or no], glomerular filtration rate missing indicator [yes or no], smoking status missing indicator [yes or no], alcoholic drinks missing indicator [yes or no], and aerobic physical activity missing indicator [yes or no]).

[b] **Model 2 (replication cohort A):** Adjusted for age (20–39, 40–59, and ≥60 years); sex; Charlson comorbidity index (0, 1, and ≥ 2); BMI (underweight [<18.5 kg/m^2], normal [18.5−23.0 kg/m^2], overweight [23.0–25.0 kg/m^2], obese [≥25.0 kg/m^2], and unknown); blood pressure (systolic blood pressure <140 mmHg and diastolic blood pressure <90 mmHg, systolic blood pressure ≥140 mmHg or diastolic blood pressure ≥90 mmHg, and unknown); fasting blood glucose (<100, ≥100 mg/dL, and unknown); serum total cholesterol (<200, 200–240, ≥240 mg/dL, and unknown); glomerular filtration rate (<60, 60−90, ≥90 mL/min/1.73 m^2, and unknown); smoking status (non- and current smoker, and unknown); alcoholic drinks (<1, 1–2, 3–4, ≥5 days per week, and unknown); aerobic physical activity (sufficient, insufficient, and unknown); previous history of cardiovascular disease, chronic kidney disease, and chronic obstructive pulmonary disease; history of medication use for diabetes mellitus, dyslipidemia, and hypertension; and missing indicators (BMI missing indicator [yes or no], blood pressure missing indicator [yes or no], fasting blood glucose missing indicator [yes or no], serum total cholesterol missing indicator [yes or no], glomerular filtration rate missing indicator [yes or no], smoking status missing indicator [yes or no], alcoholic drinks missing indicator [yes or no], and aerobic physical activity missing indicator [yes or no]).

[b] **Model 2 (replication cohort B):** Adjusted for age (20–39, 40–59, and ≥ 60 years); sex; household income (<£18,000, £18,000–£30,999, £31,000–£51,999, £52,000–£100,000, >£100,000), and unknown); region of residence (urban and rural); townsend deprivation index (T1 [last deprived], T2, T3 [most deprived], and unknown); race (white, mixed, Asian, black, others, and unknown); Charlson comorbidity index (0, 1, and ≥ 2); BMI (normal [<25.0 kg/m^2], overweight [25.0–30.0 kg/m^2], obese [≥ 30.0 kg/m^2], and unknown); education levels (≤10, 11-12, >12, and unknown); blood pressure (systolic blood pressure < 140 mmHg and diastolic blood pressure < 90 mmHg, systolic blood pressure ≥ 140 mmHg or diastolic blood pressure ≥ 90 mmHg, and unknown); fasting blood glucose (<100, ≥100 mg/dL, and unknown); smoking status (non- and current smoker, and unknown); alcohol consumption (every day, sometimes, rarely days per week, and unknown); aerobic physical activity (low, moderate, high, and unknown); previous history of cardiovascular disease, chronic kidney disease, and chronic obstructive pulmonary disease; history of medication use for diabetes mellitus, dyslipidemia, and hypertension; and missing indicators (household income missing

indicator [yes or no], townsend deprivation index missing indicator [yes or no], race missing indicator [yes or no], education levels [yes or no], obesity missing indicator [yes or no], blood pressure missing indicator [yes or no], fasting blood glucose missing indicator [yes or no], serum total cholesterol missing indicator [yes or no], glomerular filtration rate missing indicator [yes or no], smoking status missing indicator [yes or no], alcoholic drinks missing indicator [yes or no], and aerobic physical activity missing indicator [yes or no]).

Table S30. The HR with 95% CI for the long-term sequelae risk of **incident allergic disease by vaccine dose** following COVID-19 diagnosis in the propensity score-matched main cohort (South Korea).

Factors	Group	Incidence rate*	HR (95% CI)	
			Model 1 [a]	Model 2 [b]
**Allergic disease**				
Number of SARS-CoV-2 vaccinations	Non-infected control without vaccination	15.5	1.00 (reference)	1.00 (reference)
	Patients infected COVID-19 without vaccination	29.7	**1.92 (1.66 to 2.22)**	**1.94 (1.67 to 2.24)**
	Non-infected control with vaccination 1 time	29.8	1.00 (reference)	1.00 (reference)
	Patients infected COVID-19 with vaccination 1 time	35.6	1.14 (0.82 to 1.59)	1.16 (0.83 to 1.62)
	Non-infected control with vaccination ≥2 times	24.5	1.00 (reference)	1.00 (reference)
	Patients infected COVID-19 with vaccination ≥2 times	21.4	**0.80 (0.64 to 1.00)**	**0.80 (0.64 to 1.00)**
**Asthma**				
Number of SARS-CoV-2 vaccinations	Non-infected control without vaccination	0.6	1.00 (reference)	1.00 (reference)
	Patients infected COVID-19 without vaccination	2.1	**3.18 (1.57 to 6.45)**	**3.40 (1.68 to 6.91)**
	Non-infected control with vaccination 1 time	4.1	1.00 (reference)	1.00 (reference)
	Patients infected COVID-19 with vaccination 1 time	3.3	0.69 (0.20 to 2.32)	0.77 (0.23 to 2.60)
	Non-infected control with vaccination ≥2 times	1.0	1.00 (reference)	1.00 (reference)
	Patients infected COVID-19 with vaccination ≥2 times	1.8	1.63 (0.57 to 4.64)	1.62 (0.56 to 4.62)
**Allergic rhinitis**				
Number of SARS-CoV-2 vaccinations	Non-infected control without vaccination	10.5	1.00 (reference)	1.00 (reference)
	Patients infected COVID-19 without vaccination	22.0	**2.14 (1.79 to 2.55)**	**2.15 (1.80 to 2.56)**

	Non-infected control with vaccination 1 time	20.0	1.00 (reference)	1.00 (reference)
	Patients infected COVID-19 with vaccination 1 time	25.2	1.22 (0.82 to 1.82)	1.24 (0.83 to 1.85)
	Non-infected control with vaccination ≥2 times	17.7	1.00 (reference)	1.00 (reference)
	Patients infected COVID-19 with vaccination ≥2 times	16.4	0.86 (0.66 to 1.12)	0.85 (0.65 to 1.12)
**Atopic dermatitis**				
Number of SARS-CoV-2 vaccinations	Non-infected control without vaccination	2.3	1.00 (reference)	1.00 (reference)
	Patients infected COVID-19 without vaccination	3.2	**1.47 (1.00 to 2.16)**	**1.49 (1.01 to 2.20)**
	Non-infected control with vaccination 1 time	1.8	1.00 (reference)	1.00 (reference)
	Patients infected COVID-19 with vaccination 1 time	3.3	1.94 (0.61 to 6.17)	2.04 (0.64 to 6.45)
	Non-infected control with vaccination ≥2 times	2.6	1.00 (reference)	1.00 (reference)
	Patients infected COVID-19 with vaccination ≥2 times	1.7	0.68 (0.36 to 1.28)	0.67 (0.36 to 1.27)
**Food allergy**				
Number of SARS-CoV-2 vaccinations	Non-infected control without vaccination	2.4	1.00 (reference)	1.00 (reference)
	Patients infected COVID-19 without vaccination	3.3	1.25 (0.86 to 1.82)	1.26 (0.86 to 1.83)
	Non-infected control with vaccination 1 time	5.1	1.00 (reference)	1.00 (reference)
	Patients infected COVID-19 with vaccination 1 time	4.1	0.69 (0.30 to 1.59)	0.69 (0.30 to 1.60)
	Non-infected control with vaccination ≥2 times	3.7	1.00 (reference)	1.00 (reference)
	Patients infected COVID-19 with vaccination ≥2 times	1.6	**0.38 (0.20 to 0.71)**	**0.38 (0.20 to 0.71)**

BMI, body mass index; CI, confidence interval; HR, hazard ratio.

* Incidence rate is expressed as per 1,000 person-years.

[a] **Model 1**: Adjusted for age (20–39, 40–59, and ≥60 years) and sex.

[b] **Model 2 (South Korea)**: Adjusted for age (20–39, 40–59, and ≥60 years); sex; household income (low income, middle income, and high income); region of residence (urban and rural); Charlson comorbidity index (0, 1, and ≥2); BMI (underweight [<18.5 kg/m^2], normal [18.5–23.0 kg/m^2], overweight [23.0–25.0 kg/m^2], obese [≥25.0 kg/m^2], and unknown); blood pressure (systolic blood pressure <140 mmHg and diastolic blood pressure <90 mmHg, systolic blood pressure ≥140 mmHg or diastolic blood pressure ≥90 mmHg, and unknown); fasting blood glucose (<100, ≥100 mg/dL, and unknown); serum total cholesterol (<200, 200–240, ≥240 mg/dL, and unknown); glomerular filtration rate (<60, 60−90, ≥90 mL/min/1.73 m^2, and unknown); smoking status (non-, ex-, current smoker, and unknown); alcoholic drinks (<1, 1–2, 3‒4, ≥5 days per week, and unknown); aerobic physical activity (sufficient, insufficient, and unknown); previous history of cardiovascular disease, chronic kidney disease, and chronic obstructive pulmonary disease; history of medication use for diabetes mellitus, dyslipidemia, and hypertension; and missing indicators (BMI missing indicator [yes or no], blood pressure missing indicator [yes or no], fasting blood glucose missing indicator [yes or no], serum total cholesterol missing indicator [yes or no], glomerular filtration rate missing indicator [yes or no], smoking status missing indicator [yes or no], alcoholic drinks missing indicator [yes or no], and aerobic physical activity missing indicator [yes or no]).

Table S31. The HR with 95% CI for the long-term sequelae risk of **incident allergic disease by mRNA vaccine dose** over time following COVID-19 diagnosis in the propensity score-matched main cohort (South Korea).

Factors	Group	Incidence rate *	HR (95% CI)	
			Model 1 [a]	Model 2 [b]
**Allergic disease**				
Number of SARS-CoV-2 vaccinations	Non-infected control	19.5	1.00 (reference)	1.00 (reference)
Vaccination 1 time	Patients infected with COVID-19 within 30 days	31.8	**1.70 (1.30 to 2.23)**	**1.69 (1.29 to 2.22)**
	Patients infected with COVID-19 after 30 days	23.9	1.24 (0.71 to 2.16)	1.23 (0.71 to 2.14)
Vaccination ≥2 times	Patients infected with COVID-19 within 30 days	20.6	1.01 (0.59 to 1.72)	0.97 (0.57 to 1.67)
	Patients infected with COVID-19 after 30 days	25.9	1.27 (0.87 to 1.86)	1.26 (0.86 to 1.84)
**Asthma**				
Number of SARS-CoV-2 vaccinations	Non-infected control	0.7	1.00 (reference)	1.00 (reference)
Vaccination 1 time	Patients infected with COVID-19 within 30 days	2.5	**4.35 (1.51 to 12.53)**	**3.94 (1.35 to 11.51)**
	Patients infected with COVID-19 after 30 days	1.8	3.17 (0.41 to 24.71)	3.20 (0.41 to 25.23)
Vaccination ≥2 times	Patients infected with COVID-19 within 30 days	1.5	1.85 (0.24 to 14.34)	1.71 (0.22 to 13.37)
	Patients infected with COVID-19 after 30 days	0.9	1.36 (0.18 to 10.52)	1.39 (0.18 to 10.79)
**Allergic rhinitis**				
Number of SARS-CoV-2 vaccinations	Non-infected control	14.6	1.00 (reference)	1.00 (reference)
Vaccination 1 time	Patients infected with COVID-19 within 30 days	24.2	**1.72 (1.26 to 2.34)**	**1.72 (1.26 to 2.35)**
	Patients infected with COVID-19 after 30 days	20.3	1.37 (0.75 to 2.51)	1.36 (0.75 to 2.50)

Vaccination ≥2 times	Patients infected with COVID-19 within 30 days	19.1	1.27 (0.73 to 2.22)	1.24 (0.71 to 2.17)
	Patients infected with COVID-19 after 30 days	21.4	1.40 (0.92 to 2.12)	1.37 (0.90 to 2.09)
**Atopic dermatitis**				
Number of SARS-CoV-2 vaccinations	Non-infected control	2.2	1.00 (reference)	1.00 (reference)
Vaccination 1 time	Patients infected with COVID-19 within 30 days	2.0	1.01 (0.36 to 2.84)	1.01 (0.36 to 2.84)
	Patients infected with COVID-19 after 30 days	0.0	NA	NA
Vaccination ≥2 times	Patients infected with COVID-19 within 30 days	0.0	NA	NA
	Patients infected with COVID-19 after 30 days	1.8	0.81 (0.19 to 3.35)	0.80 (0.19 to 3.34)
**Food allergy**				
Number of SARS-CoV-2 vaccinations	Non-infected control	2.4	1.00 (reference)	1.00 (reference)
Vaccination 1 time	Patients infected with COVID-19 within 30 days	3.5	1.52 (0.68 to 3.39)	1.40 (0.63 to 3.13)
	Patients infected with COVID-19 after 30 days	1.8	0.77 (0.11 to 5.60)	0.74 (0.10 to 5.37)
Vaccination ≥2 times	Patients infected with COVID-19 within 30 days	0.0	NA	NA
	Patients infected with COVID-19 after 30 days	1.8	0.74 (0.18 to 3.04)	0.73 (0.18 to 3.01)

BMI, body mass index; CI, confidence interval; HR, hazard ratio.

* Incidence rate is expressed as per 1,000 person-years.

The data in bold indicate significant differences ($P < 0.05$).

[a] **Model 1**: Adjusted for age (20–39, 40–59, and ≥60 years) and sex.

[b] **Model 2**: Adjusted for age (20–39, 40–59, and ≥60 years); sex; household income (low income, middle income, and high income); region of residence (urban and rural); Charlson comorbidity index (0, 1, and ≥2); BMI (underweight [<18.5 kg/m^2], normal [18.5–23.0 kg/m^2], overweight [23.0–25.0 kg/m^2], obese [≥25.0 kg/m^2], and unknown); blood pressure (systolic blood pressure <140 mmHg and diastolic blood pressure <90 mmHg, systolic blood pressure ≥140 mmHg or

diastolic blood pressure ≥90 mmHg, and unknown); fasting blood glucose (<100, ≥100 mg/dL, and unknown); serum total cholesterol (<200, 200–240, ≥240 mg/dL, and unknown); glomerular filtration rate (<60, 60−90, ≥90 mL/min/1.73 m^2, and unknown); smoking status (non-, ex-, current smoker, and unknown); alcoholic drinks (<1, 1–2, 3–4, ≥5 days per week, and unknown); aerobic physical activity (sufficient, insufficient, and unknown); previous history of cardiovascular disease, chronic kidney disease, and chronic obstructive pulmonary disease; history of medication use for diabetes mellitus, dyslipidemia, and hypertension; and missing indicators (BMI missing indicator [yes or no], blood pressure missing indicator [yes or no], fasting blood glucose missing indicator [yes or no], serum total cholesterol missing indicator [yes or no], glomerular filtration rate missing indicator [yes or no], smoking status missing indicator [yes or no], alcoholic drinks missing indicator [yes or no], and aerobic physical activity missing indicator [yes or no]).

Supplement material. Explanation of replication cohorts (Japan and the UK).

JMDC (replication cohort A)

Data source

Japan has health insurance provided by the universal insurance system.[1] The JMDC has contracts with over 60 insurance providers and includes health insurance claims records data of insured individuals who are primarily employees of relatively large companies in Japan.[2] JMDC has created a database, using data collected from medical institutions in Japan, consisting of patient-level data (unique identifier, family identifiers, relationship to the insured individual, age, sex) and claims for inpatient and outpatient treatment (disease class according to International Classification of Diseases [ICD]-10 code, prescribed drugs based on Anatomical Therapeutic Chemical class, and drug dosage form), diagnosis or therapeutic procedure, institutional information (hospital character), and health checkup (i.e., body mass index, blood pressure, clinical laboratory test, medication status, and self-administered questionnaire such as smoking status, alcohol consumption, and physical activity).[3]

For more information, please see this webpage (https://www.jmdc.co.jp/en/jmdc-claims-database/).

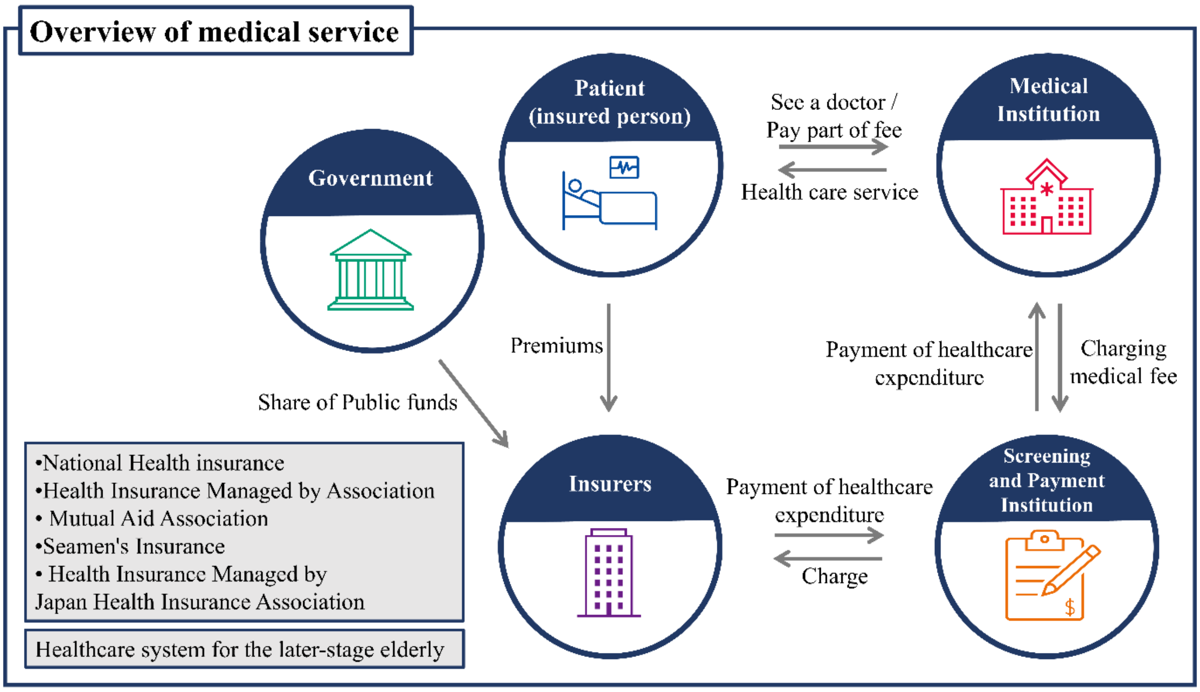

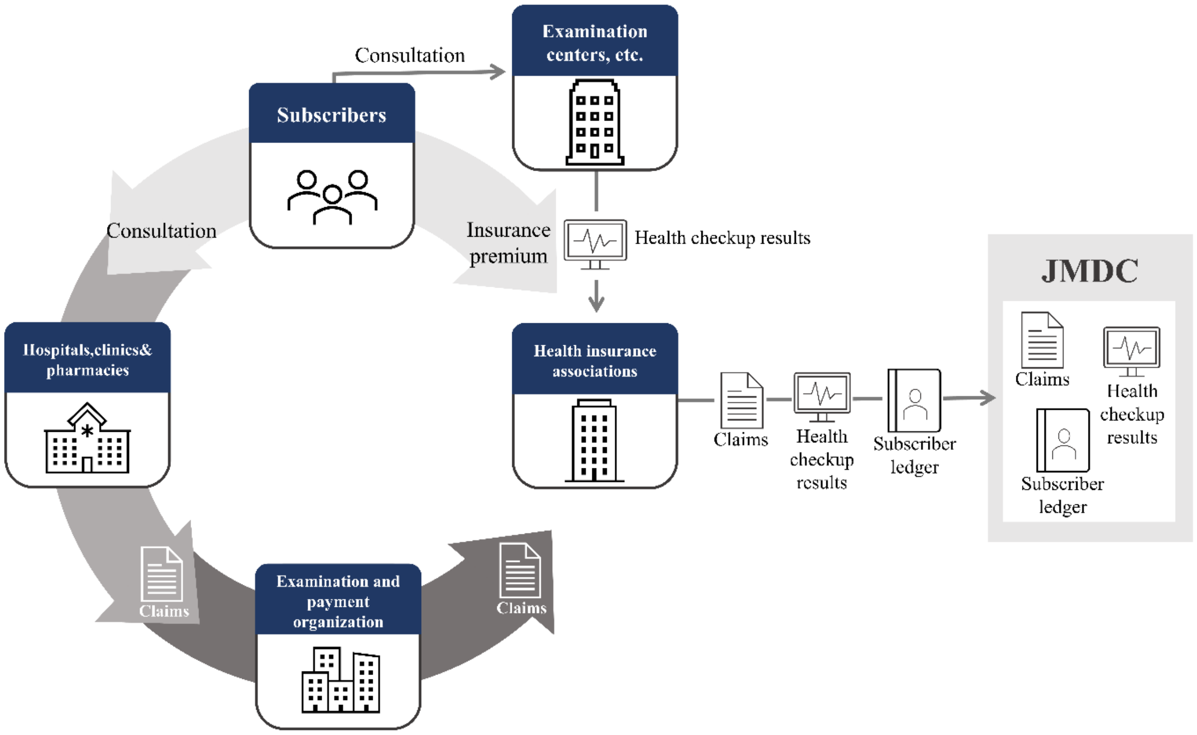

Covariates

The demographic characteristics of the participants were obtained from the health JMDC database as followings: age (20–39, 40–59, and ≥60 years), sex, Charlson comorbidity index (0, 1, and ≥ 2), BMI (underweight [<18.5 kg/m^2], normal [18.5–23.0 kg/m^2], overweight [23.0–25.0 kg/m^2], obese [≥25.0 kg/m^2], and unknown), blood pressure (systolic blood pressure <140 mmHg and diastolic blood pressure <90 mmHg, systolic blood pressure ≥140 mmHg or diastolic blood pressure ≥90 mmHg, and unknown), fasting blood glucose (<100, ≥100 mg/dL, and unknown), serum total cholesterol (<200, 200–240, ≥240 mg/dL, and unknown), glomerular filtration rate (<60, 60–90, ≥90 mL/min/1.73 m^2, and unknown), smoking status (non- and current smoker, and unknown), alcoholic drinks (<1, 1–2, 3–4, ≥5 days per week, and unknown), aerobic physical activity (sufficient, insufficient, and unknown), previous history of cardiovascular disease, chronic kidney disease, and chronic obstructive pulmonary disease, history of medication use for diabetes mellitus, dyslipidemia, and hypertension, and missing indicators (BMI missing indicator [yes or no], blood pressure missing indicator [yes or no], fasting blood glucose missing indicator [yes or no], serum total cholesterol missing indicator [yes or no], glomerular filtration rate missing indicator [yes or no], smoking status missing

indicator [yes or no], alcoholic drinks missing indicator [yes or no], and aerobic physical activity missing indicator [yes or no]).

UK Biobank (replication cohort B)

Data source

The UK Biobank, which is one of the datasets that rely on voluntary participation, is a globally recognized institution providing unique and extensive data for large-scale research through biomedical samples and health information.[4] Launched in 2006 in the UK, this project has engaged numerous researchers who utilize participant health data and biological samples for diverse medical studies.[5] With over 500,000 participants recruited nationwide, the cohort represents rich diversity in terms of age, race, gender, and geographical location, ensuring the applicability of research outcomes to diverse populations.[6] Participants have provided a range of health-related data, including health surveys, physical measurements, blood pressure readings, and the provision of blood and urine samples. This invaluable data is employed in research across various fields, such as genomic analysis, cardiovascular and metabolic diseases, and cancer research. Genomic data collected from participants contribute to studying genetic traits and variations, providing crucial information for understanding genetic contributions and exploring interactions between genes and environmental factors.[7]

For more information, please see this webpage (https://www.ukbiobank.ac.uk/learn-more-about-uk-biobank).

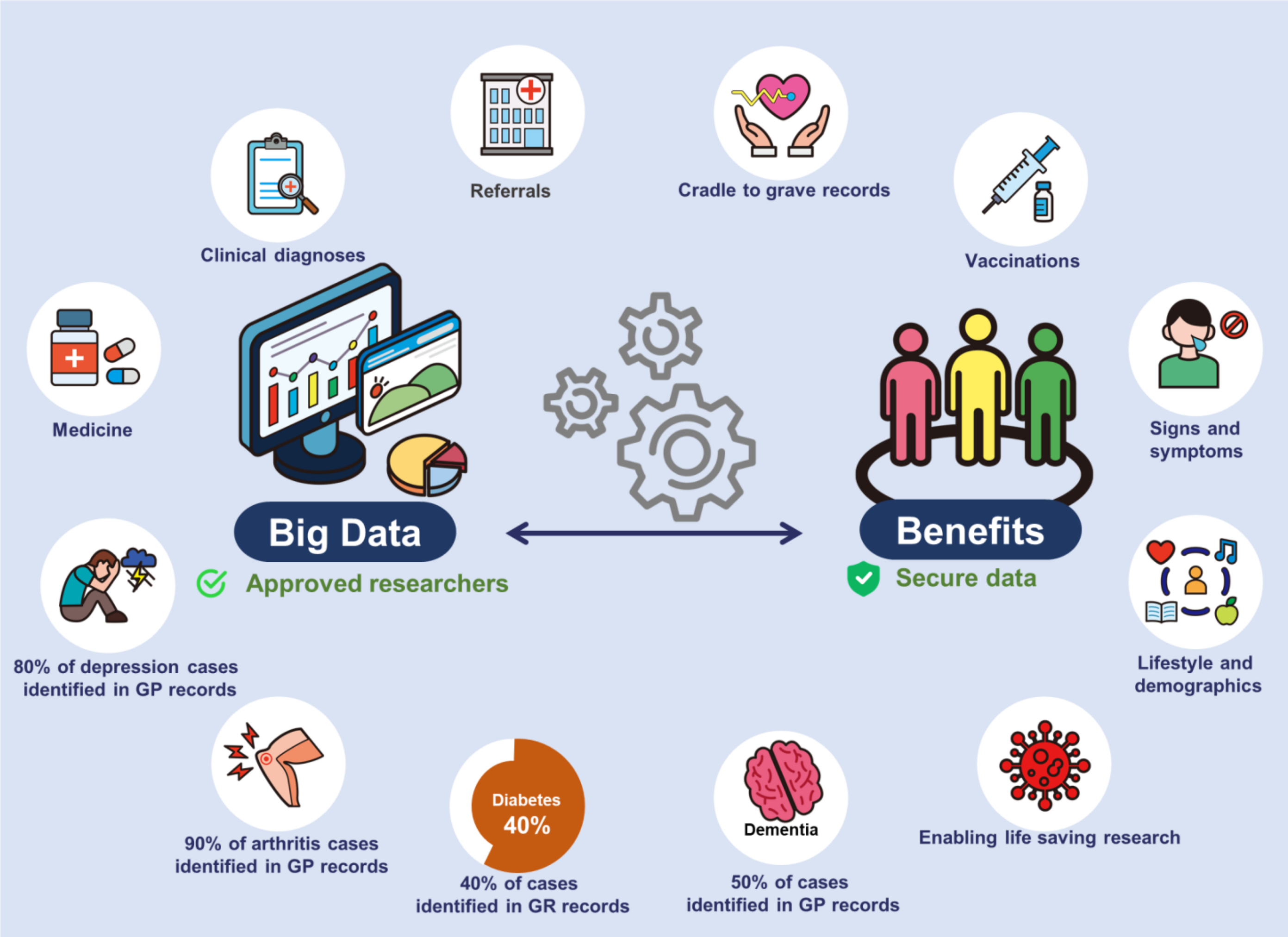

Covariates

The demographic characteristics of the participants were obtained from the health UK biobank database as followings: age (20–39, 40–59, and ≥ 60 years), sex, household income (<£18,000, £18,000–£30,999, £31,000–£51,999, £52,000–£100,000, >£100,000), and unknown), region of residence (urban and rural), townsend deprivation index (T1 [last deprived], T2, T3 [most deprived], and unknown), race (white, mixed, Asian, black, others, and unknown), Charlson comorbidity index (0, 1, and ≥ 2), BMI (normal [<25.0 kg/m^2], overweight [25.0–30.0 kg/m^2], obese [≥ 30.0 kg/m^2], and unknown), education levels (≤10, 11-12, >12, and unknown), blood pressure (systolic blood pressure < 140 mmHg and diastolic blood pressure < 90 mmHg, systolic blood pressure ≥ 140 mmHg or diastolic blood pressure ≥ 90 mmHg, and unknown), fasting blood glucose (<100, ≥100 mg/dL, and unknown), smoking status (non- and current smoker, and unknown), alcohol

consumption (every day, sometimes, rarely days per week, and unknown), aerobic physical activity (low, moderate, high, and unknown), previous history of cardiovascular disease, chronic kidney disease, and chronic obstructive pulmonary disease, history of medication use for diabetes mellitus, dyslipidemia, and hypertension, and missing indicators (household income missing indicator [yes or no], townsend deprivation index missing indicator [yes or no], race missing indicator [yes or no], education levels [yes or no], obesity missing indicator [yes or no], blood pressure missing indicator [yes or no], fasting blood glucose missing indicator [yes or no], serum total cholesterol missing indicator [yes or no], glomerular filtration rate missing indicator [yes or no], smoking status missing indicator [yes or no], alcoholic drinks missing indicator [yes or no], and aerobic physical activity missing indicator [yes or no]).

Supplement references

1. Reich MR, Ikegami N, Shibuya K, Takemi K. 50 years of pursuing a healthy society in Japan. *Lancet* 378, 1051-1053 (2011).

2. Setogawa N, Ohbe H, Isogai T, Matsui H, Yasunaga H. Characteristics and short-term outcomes of outpatient and inpatient cardiac catheterizations: A descriptive study using a nationwide claim database in Japan. *J Cardiol* 82, 201-206 (2023).

3. Kaneko H*, et al.* Medication-Naïve Blood Pressure and Incident Cancers: Analysis of 2 Nationwide Population-Based Databases. *Am J Hypertens* 35, 731-739 (2022).

4. Keyes KM, Westreich D. UK Biobank, big data, and the consequences of non-representativeness. *Lancet (London, England)* 393, 1297 (2019).

5. Backman JD*, et al.* Exome sequencing and analysis of 454,787 UK Biobank participants. *Nature* 599, 628-634 (2021).

6. Bycroft C*, et al.* The UK Biobank resource with deep phenotyping and genomic data. *Nature* 562, 203-209 (2018).

7. Ganna A, Ingelsson E. 5 year mortality predictors in 498,103 UK Biobank participants: a prospective population-based study. *Lancet (London, England)* 386, 533-540 (2015).
