# Supplementary material for: Incident allergic diseases in post-COVID-19 condition: multinational cohort studies from South Korea, Japan and the UK
Source: Nat Commun. 2024 Apr 2;15:2830. doi: 10.1038/s41467-024-47176-w (PMC10987608; doi:10.1038/s41467-024-47176-w)
Supplement: Supplementary file 3 — Reporting Summary [file 41467_2024_47176_MOESM3_ESM.pdf]

nature portfolio

Corresponding author(s): Dong Keon Yon

Last updated by author(s): Jan 17, 2024

Reporting Summary

Nature Portfolio wishes to improve the reproducibility of the work that we publish. This form provides structure for consistency and transparency in reporting. For further information on Nature Portfolio policies, see our Editorial Policies and the Editorial Policy Checklist.

Statistics

For all statistical analyses, confirm that the following items are present in the figure legend, table legend, main text, or Methods section.

n/a	Confirmed	
☐	☒	The exact sample size (*n*) for each experimental group/condition, given as a discrete number and unit of measurement
☐	☒	A statement on whether measurements were taken from distinct samples or whether the same sample was measured repeatedly
☐	☒	The statistical test(s) used AND whether they are one- or two-sided<br>*Only common tests should be described solely by name; describe more complex techniques in the Methods section.*
☐	☒	A description of all covariates tested
☐	☒	A description of any assumptions or corrections, such as tests of normality and adjustment for multiple comparisons
☐	☒	A full description of the statistical parameters including central tendency (e.g. means) or other basic estimates (e.g. regression coefficient) AND variation (e.g. standard deviation) or associated estimates of uncertainty (e.g. confidence intervals)
☐	☒	For null hypothesis testing, the test statistic (e.g. *F*, *t*, *r*) with confidence intervals, effect sizes, degrees of freedom and *P* value noted<br>*Give P values as exact values whenever suitable.*
☒	☐	For Bayesian analysis, information on the choice of priors and Markov chain Monte Carlo settings
☒	☐	For hierarchical and complex designs, identification of the appropriate level for tests and full reporting of outcomes
☒	☐	Estimates of effect sizes (e.g. Cohen's *d*, Pearson's *r*), indicating how they were calculated

Our web collection on statistics for biologists contains articles on many of the points above.

Software and code

Policy information about availability of computer code

Data collection	Data collection were performed using SAS (version 9.4; SAS Institute Inc., Cary, NC, USA) for big-data analysis.
Data analysis	Statistical analyses were performed using SAS (version 9.4; SAS Institute Inc., Cary, NC, USA) for big-data analysis. Within each cohort, a nearest-neighbor algorithm was used to perform matching between the two groups based on exposure status. This matching involved a random selection without replacement, constrained by specified caliper widths of 0.001 standard deviations. Hazard ratios (HRs) with 95% confidence intervals (CIs) using cox proportional hazard regression models were used for estimation, and were executed using SAS.

For manuscripts utilizing custom algorithms or software that are central to the research but not yet described in published literature, software must be made available to editors and reviewers. We strongly encourage code deposition in a community repository (e.g. GitHub). See the Nature Portfolio guidelines for submitting code & software for further information.

Data

Policy information about availability of data

All manuscripts must include a data availability statement. This statement should provide the following information, where applicable:

- Accession codes, unique identifiers, or web links for publicly available datasets
- A description of any restrictions on data availability
- For clinical datasets or third party data, please ensure that the statement adheres to our policy

The datasets analysed during the current study are available in the National Health Insurance Service and Korea Disease Control and Prevention Agency in South Korea (https://nhiss.nhis.or.kr/bd/ab/bdaba000eng.do.), JMDC in Japan (https://www.jmdc.co.jp/en/), and UK biobank in the UK (http://www.ukbiobank.ac.uk/).

Human research participants

Policy information about studies involving human research participants and Sex and Gender in Research.

Reporting on sex and gender

We selected the data of all individuals aged ≥20 years with COVID-19 and non-infected participants from 1 January 2020 and 31 December 2021 from multiple-national cohorts, including South Korea, Japan, and the UK. We identified "sex" for each individuals determined by the "sex" of the K-CoV-N (Korea), JMDC (Japan), and UK biobank (UK) reported to the government system.

Population characteristics

The dataset was linked and consisted of data on first general health examination results, death records, health insurance data including insurance eligibility data, personal sociodemographic data, inpatient and outpatient healthcare records, and medication records.
- Model (main cohort; South Korea): adjusted for age (20–39, 40–59, and ≥60 years); sex; household income (low income, middle income, and high income); region of residence (urban and rural); Charlson comorbidity index (0, 1, and ≥2); BMI (underweight [<18.5 kg/m2], normal [18.5–23.0 kg/m2], overweight [23.0–25.0 kg/m2], obese [≥25.0 kg/m2], and unknown); blood pressure (systolic blood pressure <140 mmHg and diastolic blood pressure <90 mmHg, systolic blood pressure ≥140 mmHg or diastolic blood pressure ≥90 mmHg, and unknown); fasting blood glucose (<100, ≥100 mg/dL, and unknown); serum total cholesterol (<200, 200–240, ≥240 mg/dL, and unknown); glomerular filtration rate (<60, 60–90, ≥90 mL/min/1.73 m2, and unknown); smoking status (non-, ex-, current smoker, and unknown); alcoholic drinks (<1, 1–2, 3–4, ≥5 days per week, and unknown); aerobic physical activity (sufficient, insufficient, and unknown); previous history of cardiovascular disease, chronic kidney disease, and chronic obstructive pulmonary disease; history of medication use for diabetes mellitus, dyslipidemia, and hypertension; and missing indicators (BMI missing indicator [yes or no], blood pressure missing indicator [yes or no], fasting blood glucose missing indicator [yes or no], serum total cholesterol missing indicator [yes or no], glomerular filtration rate missing indicator [yes or no], smoking status missing indicator [yes or no], alcoholic drinks missing indicator [yes or no], and aerobic physical activity missing indicator [yes or no]).
- Model (replication cohort A; Japan): adjusted for age (20–39, 40–59, and ≥60 years); sex; Charlson comorbidity index (0, 1, and ≥ 2); BMI (underweight [<18.5 kg/m2], normal [18.5–23.0 kg/m2], overweight [23.0–25.0 kg/m2], obese [≥25.0 kg/m2], and unknown); blood pressure (systolic blood pressure <140 mmHg and diastolic blood pressure <90 mmHg, systolic blood pressure ≥140 mmHg or diastolic blood pressure ≥90 mmHg, and unknown); fasting blood glucose (<100, ≥100 mg/dL, and unknown); serum total cholesterol (<200, 200–240, ≥240 mg/dL, and unknown); glomerular filtration rate (<60, 60–90, ≥90 mL/min/1.73 m2, and unknown); smoking status (non- and current smoker, and unknown); alcoholic drinks (drinks; <1, 1–2, 3–4, ≥5 days per week, and unknown); aerobic physical activity (sufficient, insufficient, and unknown); previous history of cardiovascular disease, chronic kidney disease, and chronic obstructive pulmonary disease; history of medication use for diabetes mellitus, dyslipidemia, and hypertension; and missing indicators (BMI missing indicator [yes or no], blood pressure missing indicator [yes or no], fasting blood glucose missing indicator [yes or no], serum total cholesterol missing indicator [yes or no], glomerular filtration rate missing indicator [yes or no], smoking status missing indicator [yes or no], alcoholic drinks missing indicator [yes or no], and aerobic physical activity missing indicator [yes or no]).
- Model (replication cohort B; UK): adjusted for age (20–39, 40–59, and ≥ 60 years); sex; household income (<£18,000, £18,000–£30,999, £31,000–£51,999, £52,000–£100,000, >£100,000), and unknown); region of residence (urban and rural); townsend deprivation index (T1[last deprived], T2, T3 [most deprived], and unknown); ethnicity (white, mixed, Asian, black, others, and unknown); Charlson comorbidity index (0, 1, and ≥ 2); BMI (normal [<25.0 kg/m2], overweight [25.0–30.0 kg/m2], obese [≥ 30.0 kg/m2], and unknown); education levels (≤10, 11-12, >12, and unknown); blood pressure (systolic blood pressure < 140 mmHg and diastolic blood pressure < 90 mmHg, systolic blood pressure ≥ 140 mmHg or diastolic blood pressure ≥ 90 mmHg, and unknown); fasting blood glucose (<100, ≥100 mg/dL, and unknown); smoking status (non- and current smoker, and unknown); alcohol consumption (every day, sometimes, rarely days per week, and unknown); aerobic physical activity (low, moderate, high, and unknown); previous history of cardiovascular disease, chronic kidney disease, and chronic obstructive pulmonary disease; history of medication use for diabetes mellitus, dyslipidemia, and hypertension; and missing indicators (household income missing indicator [yes or no], townsend deprivation index missing indicator [yes or no], ethnicity missing indicator [yes or no], education levels [yes or no], obesity missing indicator [yes or no], blood pressure missing indicator [yes or no], fasting blood glucose missing indicator [yes or no], serum total cholesterol missing indicator [yes or no], glomerular filtration rate missing indicator [yes or no], smoking status missing indicator [yes or no], alcoholic drinks missing indicator [yes or no], and aerobic physical activity missing indicator [yes or no]).

Recruitment

The study was a multinational population-based cohort study that included all adults (aged ≥ 20 years) reported to each agency during COVID-19. We used the South Korean population-based cohort (K-CoV-N; N=10,027,506) as a main cohort and the Japanese claims-based cohort (JMDC, replication cohort A; N=12,218,680) and the UK biobank cohort (UKB, replication cohort B; N=468,617). We assessed the risk of incident allergic disorder after COVID-19 diagnosis compared with contemporary controls who were not infected with SARS-CoV-2. The pre-observation period to determine the previous diagnostic history was from 2018 to 2019, and the follow-up period was from 2020 to 2021. Individuals with missing

	socioeconomic status data, those who died, or those with a history of allergy disease during the pre-observation period were excluded from the analysis. (K-COV-N excluded n=4,335,150; JMDC excluded n=2,012,073; UKB excluded n=66,974) The final sample size was 5,692,356 for K-CoV-N, 10,206,607 for JMDC, and 401,643 for UKB.
Ethics oversight	This study received approvals from Korea Disease Control and Prevention Agency (KDCA), National Health Insurance Service (NHIS; KDCA-NHIS-2022-1-632), JMDC (PHP-00002201-04), UK Biobank (94075), and the Institutional Review Board of Kyung Hee University (KHUH 2022-06-042). Under the terms of the approval, patient consent was not required for use of routine health records for our study.

Note that full information on the approval of the study protocol must also be provided in the manuscript.

Field-specific reporting

Please select the one below that is the best fit for your research. If you are not sure, read the appropriate sections before making your selection.

☒ Life sciences ☐ Behavioural & social sciences ☐ Ecological, evolutionary & environmental sciences

For a reference copy of the document with all sections, see nature.com/documents/nr-reporting-summary-flat.pdf

Life sciences study design

All studies must disclose on these points even when the disclosure is negative.

Sample size	This study utilized large-scale, population-based, multinational cohorts, including a South Korean claims-based nationwide cohort (K-CoV-N; N=10,027,506) as a main cohort and a Japanese claims-based cohort (JMDC, replication cohort A; N=12,218,680) as well as a UK prospective cohort from the UK Biobank (UKB, replication cohort B; N=468,617) as a replication cohort. They are collected over the observation period from January 1, 2018, to December 31, 2021, among individuals aged 20 and above.
Data exclusions	Individuals with missing socioeconomic status data, those who died, or those with a history of allergy disease during the pre-observation period were excluded from the analysis. (K-COV-N excluded n=4,335,150; JMDC excluded n=2,012,073; UKB excluded n=66,974) The final sample size was 5,692,356 for K-CoV-N, 10,206,607 for JMDC, and 401,643 for UKB. The final sample size was 5,692,356 for K-CoV-N, 10,206,607 for JMDC, and 401,643 for UKB.
Replication	Similar findings were reported in the replication cohorts A and B.<br>A Cox proportional hazards regression model with estimates of HRs and 95% CIs was used to explore incident overall and four subtypes (asthma, allergic rhinitis, atopic dermatitis, and food allergy) of allergic diseases associated with long COVID-19. Models were adjusted for following variables:<br>- Model (main cohort; South Korea): adjusted for age (20–39, 40–59, and ≥60 years); sex; household income (low income, middle income, and high income); region of residence (urban and rural); Charlson comorbidity index (0, 1, and ≥2); BMI (underweight [<18.5 kg/m2], normal [18.5–23.0 kg/m2], overweight [23.0–25.0 kg/m2], obese [≥25.0 kg/m2], and unknown); blood pressure (systolic blood pressure <140 mmHg and diastolic blood pressure <90 mmHg, systolic blood pressure ≥140 mmHg or diastolic blood pressure ≥90 mmHg, and unknown); fasting blood glucose (<100, ≥100 mg/dL, and unknown); serum total cholesterol (<200, 200–240, ≥240 mg/dL, and unknown); glomerular filtration rate (<60, 60–90, ≥90 mL/min/1.73 m2, and unknown); smoking status (non-, ex-, current smoker, and unknown); alcoholic drinks (<1, 1–2, 3–4, ≥5 days per week, and unknown); aerobic physical activity (sufficient, insufficient, and unknown); previous history of cardiovascular disease, chronic kidney disease, and chronic obstructive pulmonary disease; history of medication use for diabetes mellitus, dyslipidemia, and hypertension; and missing indicators (BMI missing indicator [yes or no], blood pressure missing indicator [yes or no], fasting blood glucose missing indicator [yes or no], serum total cholesterol missing indicator [yes or no], glomerular filtration rate missing indicator [yes or no], smoking status missing indicator [yes or no], alcoholic drinks missing indicator [yes or no], and aerobic physical activity missing indicator [yes or no]).<br>- Model (replication cohrot A; Japan): adjusted for age (20–39, 40–59, and ≥60 years); sex; Charlson comorbidity index (0, 1, and ≥ 2); BMI (underweight [<18.5 kg/m2], normal [18.5–23.0 kg/m2], overweight [23.0–25.0 kg/m2], obese [≥25.0 kg/m2], and unknown); blood pressure (systolic blood pressure <140 mmHg and diastolic blood pressure <90 mmHg, systolic blood pressure ≥140 mmHg or diastolic blood pressure ≥90 mmHg, and unknown); fasting blood glucose (<100, ≥100 mg/dL, and unknown); serum total cholesterol (<200, 200–240, ≥240 mg/dL, and unknown); glomerular filtration rate (<60, 60–90, ≥90 mL/min/1.73 m2, and unknown); smoking status (non- and current smoker, and unknown); alcoholic drinks (drinks; <1, 1–2, 3–4, ≥5 days per week, and unknown); aerobic physical activity (sufficient, insufficient, and unknown); previous history of cardiovascular disease, chronic kidney disease, and chronic obstructive pulmonary disease; history of medication use for diabetes mellitus, dyslipidemia, and hypertension; and missing indicators (BMI missing indicator [yes or no], blood pressure missing indicator [yes or no], fasting blood glucose missing indicator [yes or no], serum total cholesterol missing indicator [yes or no], glomerular filtration rate missing indicator [yes or no], smoking status missing indicator [yes or no], alcoholic drinks missing indicator [yes or no], and aerobic physical activity missing indicator [yes or no]).<br>- Model (replication cohort B; UK): adjusted for age (20–39, 40–59, and ≥ 60 years); sex; household income (<£18,000, £18,000–£30,999, £31,000–£51,999, £52,000–£100,000, >£100,000), and unknown); region of residence (urban and rural); townsend deprivation index (T1[last deprived], T2, T3 [most deprived], and unknown); ethnicity (white, mixed, Asian, black, others, and unknown); Charlson comorbidity index (0, 1, and ≥ 2); BMI (normal [<25.0 kg/m2], overweight [25.0–30.0 kg/m2], obese [≥ 30.0 kg/m2], and unknown); education levels (≤10, 11-12, >12, and unknown); blood pressure (systolic blood pressure < 140 mmHg and diastolic blood pressure < 90 mmHg, systolic blood pressure ≥ 140 mmHg or diastolic blood pressure ≥ 90 mmHg, and unknown); fasting blood glucose (<100, ≥100 mg/dL, and unknown); smoking status (non- and current smoker, and unknown); alcohol consumption (every day, sometimes, rarely days per week, and unknown); aerobic physical activity (low, moderate, high, and unknown); previous history of cardiovascular disease, chronic kidney disease, and chronic obstructive pulmonary disease; history of medication use for diabetes mellitus, dyslipidemia, and hypertension; and missing indicators (household income missing indicator [yes or no], townsend deprivation index missing indicator [yes or no], ethnicity missing indicator [yes or no], education levels [yes or no], obesity missing indicator [yes or no], blood pressure missing indicator [yes or no], fasting blood glucose missing indicator [yes or

	no], serum total cholesterol missing indicator [yes or no], glomerular filtration rate missing indicator [yes or no], smoking status missing indicator [yes or no], alcoholic drinks missing indicator [yes or no], and aerobic physical activity missing indicator [yes or no]).
Randomization	Not applicable; individuals were recruited in an observational study with convenience samples being collected.
Blinding	Blinding was not relevant, since this is an observational study, where participants were invited based on test status (case or control)

Reporting for specific materials, systems and methods

We require information from authors about some types of materials, experimental systems and methods used in many studies. Here, indicate whether each material, system or method listed is relevant to your study. If you are not sure if a list item applies to your research, read the appropriate section before selecting a response.

Materials & experimental systems

n/a	Involved in the study	
☒	☐	Antibodies
☒	☐	Eukaryotic cell lines
☒	☐	Palaeontology and archaeology
☒	☐	Animals and other organisms
☒	☐	Clinical data
☒	☐	Dual use research of concern

Methods

n/a	Involved in the study	
☒	☐	ChIP-seq
☒	☐	Flow cytometry
☒	☐	MRI-based neuroimaging
